# Supplementary material for: Development of copper-catalyzed deaminative esterification using high-throughput experimentation
Source: Commun Chem. 2022 Jul 19;5:83. doi: 10.1038/s42004-022-00698-0 (PMC9814592; doi:10.1038/s42004-022-00698-0)
Supplement: Supplementary file 1 — Supplementary Material [file 42004_2022_698_MOESM1_ESM.pdf]

Supplementary Information for

“Development of Copper-catalyzed Deaminative Esterification Using High-throughput Experimentation”

**S–1**	:	Table of Contents
**S–2**	:	Supplementary Methods
**S–3**	:	Experimental
**S–23**	:	Characterization
**S–58**	:	Supplementary Note 1: Spectra
**S–128**	:	Supplementary References

Supplementary Methods

All reactions were conducted in oven- or flame-dried glassware under an atmosphere of nitrogen unless stated otherwise. Reactions were set up in an MBraun LABmaster Pro Glove Box (H_2O level <0.1 ppm, O_2 level <0.1 ppm), or using standard Schlenk technique with a glass vacuum manifold connected to an inlet of dry nitrogen gas. Solvents (acetonitrile, tetrahydrofuran) were purified using a MBraun SPS solvent purification system, by purging with nitrogen, and then passing the solvent through a column of activated alumina. Acetone was distilled from anhydrous calcium sulfate. Reagents were purchased from Sigma Aldrich, Alfa Aesar, Oakwood Chemical, or TCI Chemical. All chemicals were used as received, except for liquid anilines, which were passed through a small plug of neutral alumina prior to reaction or recrystallized before reactions (8-aminoquinooline, and *p*-anisidine). Glass1-dram (Fisherbrand™ parts No. 03-339-21B) or 2-dram vials (Fisherbrand™ parts No. 03-339-21D) were used as reaction vessels, fitted with a screwcaps with Teflon-coated silicone septa (CG-4910-02), and magnetic stir bars (Fisher Scientific #14-513-93 or #14-513-65).

Proton nuclear magnetic resonance spectra (^{1}H NMR) were recorded on a Varian MR-500 MHz or Varian MR-700 MHz spectrometer and chemical shifts are reported in parts per million (ppm) using the solvent residual peak as an internal standard ($CDCl_3$ at 7.26 ppm). Data are reported using the abbreviations: app = apparent, s = singlet, d = doublet, t = triplet, q = quartet, m = multiplet, comp = complex, br = broad. Coupling constant(s) are reported in Hz. Proton-decoupled carbon nuclear magnetic resonance spectra (^{13}C NMR) spectra were recorded on a Varian MR-500 MHz or Varian MR-700 MHz spectrometer and chemical shifts are reported in ppm using the solvent as an internal standard ($CDCl_3$ at 77.16 ppm). High resolution mass spectrometry data (HRMS) was obtained on an Agilent 6230 TOF LC/MS equipped with ESI detector in positive mode and on an Agilent 6520 Accurate-Mass Q-TOF LC/MS equipped with APCI (positive ionization mode). Reaction analysis was typically performed by thin-layer chromatography on silica gel or using a Waters I-class ACQUITY UPLC-MS (Waters Corporation, Milford, MA, USA) equipped with in-line photodiode array detector (PDA) and QDa mass detector (ESI positive ionization mode). 0.1 µL sample injections were taken from acetonitrile solutions of reaction mixtures or products (~1 mg/mL). A partial loop injection mode was used with the needle placement at 1.0 mm from bottom of the wells and a 0.2 µL air gap at pre-aspiration and post-aspiration. Column used: Waters Cortecs UPLC C18+ column, 2.1mm × 50 mm with (Waters #186007114) with Waters Cortecs UPLC C18+ VanGuard Pre-column 2.1mm × 5 mm (Waters #186007125), Mobile Phase A: 0.1 % formic acid in Optima LC/MS-grade water, Mobile Phase B: 0.1% formic acid in Optima LC/MS-grade MeCN. Flow rate: 1 mL/min. Column temperature: 45 °C. The PDA sampling rate was 20 points/sec. The QDa detector monitored m/z 150-750 with a scan time of 0.06 seconds and a cone voltage of 30 V. The PDA detector range was between 210 nm – 400 nm with a resolution of 1.2 nm. A two-minute method was used and the method gradients are below: 0 min: 0.8 mL/min, 95% 0.1% formic acid in water/5% 0.1% formic acid in acetonitrile; 1.5 min : 0.8 mL/min, 0.1% 0.1% formic acid in water/99.9% 0.1% formic acid in acetonitrile; 1.91 min : 0.8 mL/min, 95% 0.1% formic acid in water/5% 0.1% formic acid in acetonitrile. UV-Vis absorbance spectra were measured on a BioTek Epoch microplate spectrophotometer using monochromator wavelength scan selection in the Multiscreen 96-well transport receiver plate (cat No. MATRNPS50). In spectra scanning reading mode, with the wavelength range set to 200 – 999 nm with 10 nm scan rate. Data was processed in Gen5 software and csv file was generated as the output for visualization in python.

Flash chromatography was performed on silica gel (230 – 400 Mesh, Grade 60) under a positive pressure of Nitrogen. Thin Layer Chromatography was performed on 25 µm TLC Silica gel 60 F_{254} glass plates purchased from Fisher Scientific (part number: S07876). Visualization was performed using ultraviolet light (254 nm), potassium permanganate ($KMnO_4$) stain, or iodine stain.

Experimental

Data Visualization. Once the desired characterization data of each high-throughput experimentation or ultra-high throughput experimentation was captured, various graphical and statistical methods were used for analysis. Analytical Studio Pro from Virscidian (version 10.8) was used to process the UPLC data files in total wavelength chromatogram (TWC), and to generate python importable reports in .csv files. Code for chemoinformatics and visualization was written in Python (version 3.9.7). All Python dependencies were installed using Conda, version 4.10.3, installed via Miniforge's arm64 distribution. RDKIT (version 2021.9.2) was utilized to calculate physicochemical properties and perform computational reactions. Matplotlib (version 3.4.3) and Seaborn (version 0.11.2) were both used to create plots and graphs. Numpy (version 1.21.4) and Sklearn, installed via scikit-learn (version 1.0.1) were used to calculate the tSNE dimensionality reduction. Openpyxl (version 3.0.9) and Pandas (1.3.4) were used to parse excel files and other data formats. Probabilistic multi-parameter optimization of central nervous system modeling was performed using Merck & Co., Inc's pMPO package (available at https://github.com/Merck/pmpo). Physicochimcal properites used to calculate PMO were polar surface area, hydrogen bond donors, hydrogen bond acceptors, and molecular weight, all of which were calculated by RDKIT after ionization at pH 7.4. LogD calculations and ionization of compounds were achieved utilizing Dassault Système's Pipeline Pilot (2019 version).

High-Throughput Experimentation for the Optimization of Copper-Catalyzed Esterification. High-throughput experiments were performed according to reported methods.[1,2] Stock solutions were prepared according to the recipes generated in phactor™[3] with the color schemes shown below. In an inert atmosphere glovebox, reagents were weighed and dissolved or suspended in anhydrous degassed acetonitrile in flame-dried one-dram or two-dram vials to achieve the stock solution concentrations shown in individual tables below. Stock solutions or suspensions of reagents were stirred until either clear solutions or uniform slurries were achieved. A 96-well aluminum Para-dox® reaction block (Analytical Sales & Services cat. No. 96960) was equipped with oven-dried shell vials (Analytical Sales & Services parts No. 884001), then moved into the glove box. Stock solutions were dosed into the appropriate shell vials according to the recipes shown below manually using single channel micropipettes. A parylene-coated stir dowel (V&P SCIENTIFIC parts No. VP711D-1) was then added to each vial. The reaction block was then sealed according to the manufacturer's directions, removed from the glove box and stirred at room temperature overnight on a tumble stirrer (V&P SCIENTIFIC parts No. VP710E5X) stirring at 800 rpm.

To determine assay yields, a calibration curve was produced by measure the UV response factor of the caffeine internal standard relative to varying concentrations of purified ester product **6**. The ratio of ester product over caffeine internal standard response was determined as follows. Standard solutions of pure ester (**6**, 0.200 M) were prepared in acetonitrile and diluted to 0.15 M, 0.10 M, 0.075 M, 0.050 M, 0.038 M, 0.025 M, 0.013 M, 0.063 M, respectively, by serial dilution. From these, UPLC-MS samples were prepared by adding an aliquot (100 μL) of thus prepared standard solutions to a 9:1 acetonitrile-water solution of caffeine as internal standard (0.100 M, 100 μL), mixing, and diluting an aliquot of the mixed solution (100 μL) with acetonitrile (100 μL) to a total volume of 800 μL. The samples were analyzed by UPLC-MS. Each injection was repeated three times to obtain triplicate data. The UV peak areas of ester **6** over UV peak areas of caffeine were plotted against concentration, of ester over caffeine, to generate the calibration curve, with error bars from the triplicate injection shown on the curve (Figure S1). The ratio of ester over caffeine obtained from the screen was then converted into assay yields according to the curve's linear regression formula: $UV\left(\frac{ester}{caffeine}\right) = 0.842 * concentration\left(\frac{ester}{caffeine}\right) + 0.025$. The reaction well observed no formation of ester in the screen will be assigned with 0.

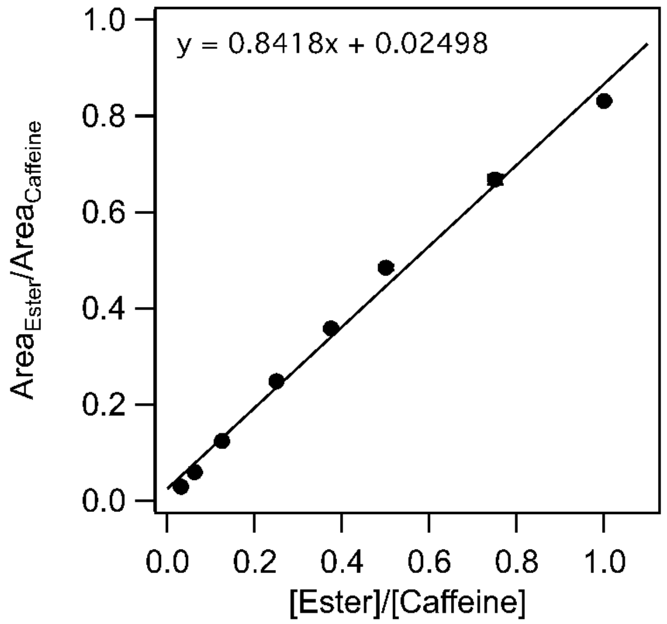

Figure S1 Calibration Curve with Caffeine as the Internal Standard to Determine Assay Yield

YS01208_recipe.csv

- acetonitrile control
- 4,4'-DI-TERT-BUTYL-2,2'-DIPYRIDYL, 98%
- 2,6-BIS[(4S)-(-)-ISOPROPYL-2-OXAZOLIN-2-YL]PYRIDINE, 99%
- 2,2-BIS((4S)-(-)-4-ISOPROPYLOXAZOLINE)PROPANE, 96%
- 2,2':6',2"-TERPYRIDINE, 98%
- 4,7-DIMETHOXY-1,10-PHENANTHROLINE, 97%
- COPPER(I) BROMIDE, 98.0+% (RT)
- COPPER(I) CYANIDE, 99%
- COPPER(I) CHLORIDE, 99.995+%, TRACE METALS BASIS
- COPPER(I) IODIDE 99.995% (TRACE METAL BASIS)
- COPPER(I) OXIDE, 99.99+%, ANHYDROUS, TRACE METALS BASIS
- COPPER(I) THIOCYANATE, 99%
- COPPER(I) THIOPHENE-2-CARBOXYLATE
- COPPER(II) BROMIDE
- COPPER(II) CHLORIDE
- COPPER(II) PHTHALOCYANINE, 99+%
- TETRAKIS(ACETONITRILE)COPPER(I) HEXA-FLUOROPHOSPHATE, 97%
- TETRAKIS(ACETONITRILE)COPPER(I) TETRAFLUOROBORATE, 99%
- COPPER(I) TRIFLUOROMETHANESULFONATE BENZENE COMPLEX, 90%, TECH.
- Copper(II) Trifluoromethanesulfonate
- Copper(II) acetylacetonate 97%
- COPPER(II) ACETATE, 99.999%
- 4-me diazonium
- 2,4,6-Trimethylpyridine
- ORTHO-TOLUIC ACID, 99%

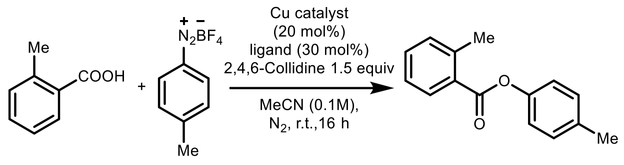

4 5 6

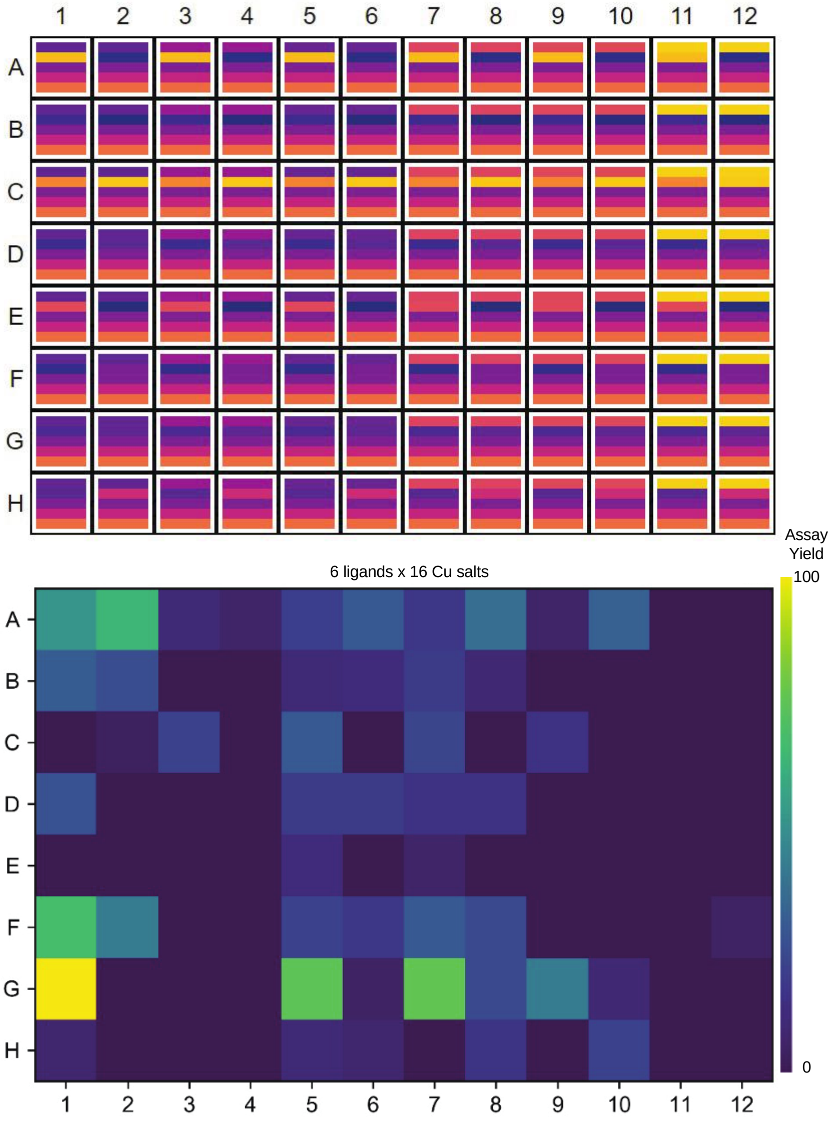

Figure S2 HTE in 96-Wellplate Screening for 16 Catalysts and 6 Ligands

Table S1 Reagents Screened in the 96-Wellplate Screening for 16 Catalysts and 6 Ligands

**Reagents**	**C_{stock} (M)**	**V_{dose} (μL)**	**Location**
Otho-toluic acid	0.50	20.0	All
4-Me diazonium	0.75	20.0	All
2,4,6-Trimethylpyridine	0.75	20.0	All

Acetonitrile control	N.A.	20.0	A1,2–H1,2
4,4'-Di-*tert*-butyl-2,2'-dipyridyl	0.20	20.0	A3,4–H3,4
2,6-Bis[(*4s*)-(-)-isopropyl-2-oxazolin-2-yl]pyridine	0.20	20.0	A5,6–H5,6
2,2-Bis((*4s*)-(-)-4-isoproppyloxazoline)propane	0.20	20.0	A7,8–H7,8
2,2':6',2''-Terpyridine	0.20	20.0	A9,10–H9,10
4,7-Dimethoxy-1,10-phenanthroline	0.20	20.0	A11,12–H11,12
Copper(I) bromide	0.10	20.0	A1,3,5,7,9,11
Copper(I) cyanide	0.10	20.0	A2,4,6,8,10,12
Copper(I) chloride	0.10	20.0	B1,3,5,7,9,11
Copper(I) iodide	0.10	20.0	B2,4,6,8,10,12
Copper(I) oxide	0.10	20.0	C1,3,5,7,9,11
Copper(I) thiocyanate	0.10	20.0	C2,4,6,8,10,12
Copper(I) thiophene-2-carboxylate	0.10	20.0	D1,3,5,7,9,11
Copper(II) bromide	0.10	20.0	D2,4,6,8,10,12
Copper(II) chloride	0.10	20.0	E1,3,5,7,9,11
Copper(II) phthalocyanine	0.10	20.0	E2,4,6,8,10,12
Tetrakis(acetonitrile)copper(I) hexafluorophosphate	0.10	20.0	F1,3,5,7,9,11
Tetrakis(acetonitrile)copper(I) tetrafluoroborate	0.10	20.0	F2,4,6,8,10,12
Copper(I) trifluoromethanesulfonate benzene complex	0.10	20.0	G1,3,5,7,9,11
Copper(II) trifluoromethanesulfonate	0.10	20.0	G2,4,6,8,10,12
Copper(II) acetylacetonate	0.10	20.0	H1,3,5,7,9,11
Copper(II) acetate	0.10	20.0	H2,4,6,8,10,12

The reactions were quenched by removing the sealing screws to open the reaction block, and exposing reactions to air, so that a 9:1 acetonitrile-water solution of caffeine as internal standard (0.100 M, 100 μL) could be added into each reaction vial and mixed evenly by pipetting up and down. Using an eight-channel multi-pipetter, from each reaction vial, an aliquot of the quenched reaction mixture (100 μL) was added into HPLC-grade acetonitrile (700 μL) to a total volume of 800 μL in a 96-well polypropylene collection plate (Analytical Sales & Services cat. No. 17P687Z) with a polypropylene cap mat on top (Analytical Sales & Services cat No. 96057). The reactions were then analyzed by UPLC-MS using the analytical method described in “Methods Summary”. First 16 copper catalysts and 6 ligands were screened as in the Figure S2 and $(CuOTf)_2$•benzene was used in 20 mol% as is (40 mol% Cu loading). The output data from HTE experiments used to generate the heatmap in Figure S2 and S3 is available in Supplementary Data file ‘HTE Data for Amine-Acid Esterification.xslx’. The wells with ligands generally gave lower conversions comparing to the wells without ligand (Figure S2 well **G1**).

YS01230-2_recipe.csv

- LITHIUM HYDROXIDE MONOHYDRATE, 98+%, ACS REAGENT
- TRIPHENYLPHOSPHINE, 99%
- 4-me diazonium
- ORTHO-TOLUIC ACID, 99%
- COPPER(I) TRIFLUOROMETHANESULFONATE BENZENE COMPLEX, 90%, TECH.
- (1R,2S)-(+)-CIS-1-AMINO-2-INDANOL, 99%
- IMes HCl
- 1,10-PHENANTHROLINE, 99+%
- 1,2-Bis(diphenylphosphino)ethane
- 2,6-DIMETHOXYANILINO(OXO)ACETIC ACID
- 4,4'-DI-TERT-BUTYL-2,2'-DIPYRIDYL, 98%
- acetonitrile control
- POTASSIUM CARBONATE, ANHYDROUS, 99.99%
- TRIETHYLAMINE, 99%
- 2-TERT-BUTYL-1,1,3,3-TETRAMETHYLGUANIDINE, 97.0+% (GC)
- 2,4,6-COLLIDINE 99%
- N,N-DIISOPROPYLETHYLAMINE, 99%, REAGENTPLUS
- POTASSIUM TERT-BUTOXIDE, 95%, REAGENT GRADE
- POTASSIUM PHOSPHATE TRIBASIC, 97%, ANHYDROUS
- CESIUM FLUORIDE, 99%
- N,N,N',N'-TETRAMETHYLETHYLENEDIAMINE, 99%, BIOREAGENT
- 1,4-DIAZABICYCLO(2.2.2)OCTANE, 98%
- CESIUM CARBONATE, 99%, REAGENTPLUS

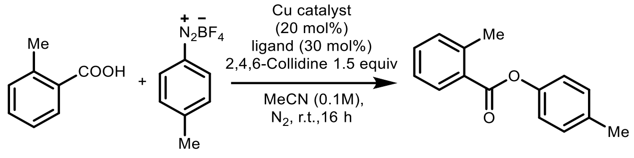

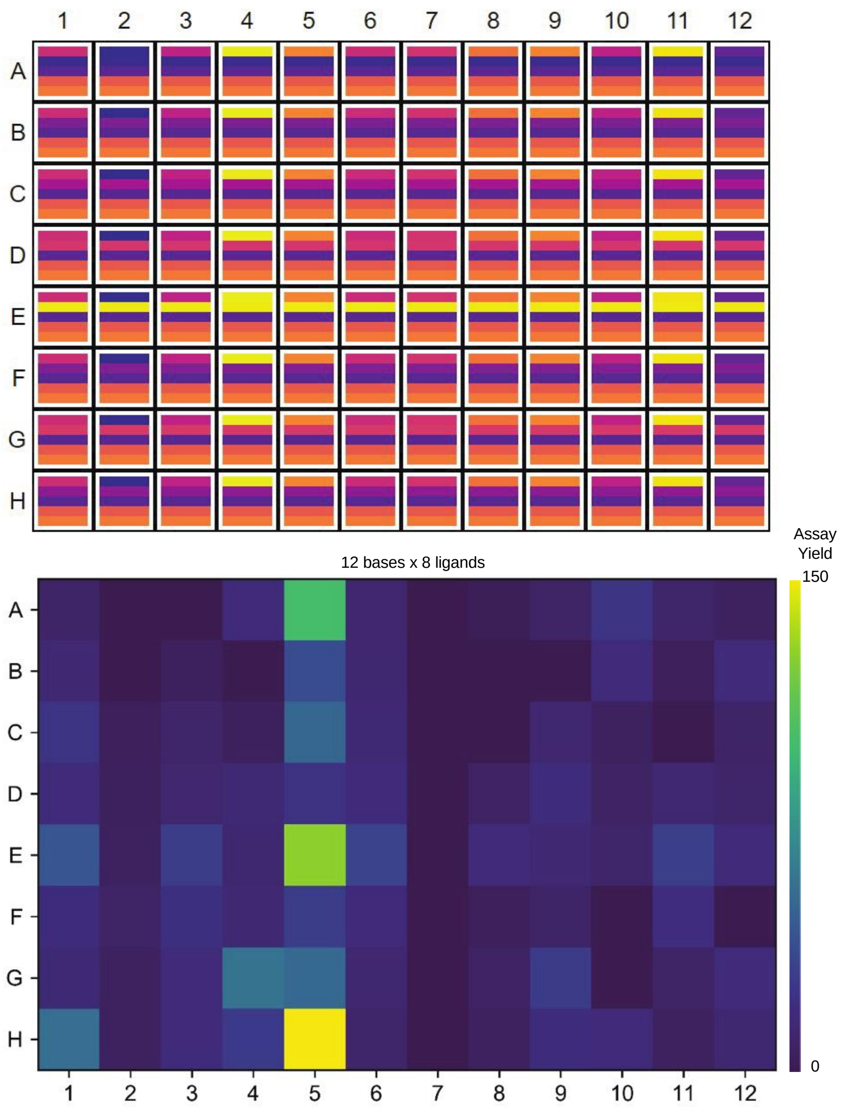

Figure S3 HTE in 96-Wellplate Screening for 12 Bases and 8 Ligands

Table S2 Reagents Screened in the 96-Wellplate Screening for 12 Bases and 8 Ligands

Reagents	C_{stock} (M)	V_{dose} (μL)	Location
Ortho-toluic acid	0.50	20.0	All
4-Me diazonium	0.75	20.0	All
Copper(I) trifluoromethanesulfonate benzene complex	0.075	20.0	All
Lithium hydroxide	0.75	20.0	A1-H1

Potassium carbonate	0.75	20.0	A2-H2
Triethylamine	0.75	20.0	A3-H3
2-*Tert*-butyl-1,1,3,3-tetramethylguanidine	0.75	20.0	A4-H4
2,4,6-collidine	0.75	20.0	A5-H5
N,N-diisopropylethylamine	0.75	20.0	A6-H6
Potassium *tert*-butoxide	0.75	20.0	A7-H7
Potassium phosphate, tribasic	0.75	20.0	A8-H8
Cesium fluoride	0.75	20.0	A9-H9
N,N,N',N'-tetramethylethylenediamine	0.75	20.0	A10-H10
1,4-Diazabicyclo(2,2,2)octane	0.75	20.0	A11-H11
Cesium carbonate	0.75	20.0	A12-H12
Triphenylphosphine	0.15	20.0	A1-12
(*1R,2S*)-(+)-*cis*-1-amino-2-indinol	0.15	20.0	B1-12
1,3-Bis(2,4,6-trimethylphenyl)imidazolinium chloride	0.15	20.0	C1-12
1,10-Phenanthroline	0.15	20.0	D1-12
1,2-Bis(diphenylphospheno)ethane	0.15	20.0	E1-12
2,6-Dimethoxylanilino(oxo)acetic acid	0.15	20.0	F1-12
4,4'-Di-*tert* butyl-2,2'-dipyridiyl	0.15	20.0	G1-12
Acetonitrile control	N.A.	20.0	H1-12

Then a follow-up HTE screen in 96-wellplate was operated to screen 12 bases (1.5 equiv) and 7 ligands (30 mol%) or no ligand on *o*-toluic acid and the diazonium salt, using 15 mol% $(CuOTf)_2$•benzene (Figure S2). The LC ratio of the product **6** was converted to the assay yield according to the calibration curve (Figure S1). The best condition in this HTE screen is with collidine (1.5 equiv) without using a ligand (Figure S3 well **H5**)

NMR Yield Determination for Reaction Condition Optimization. To optimize the catalytic method, experiments were performed wherein 1,3,5-trimethoxybenzene (0.300 equiv) was added as an internal standard prior to aqueous workup, and quantification by ^{1}H-NMR.

Table S3 Stoichiometry of Reactants and Pyridine

Stoichiometry of Pyridine

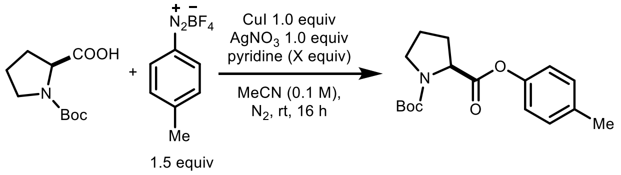

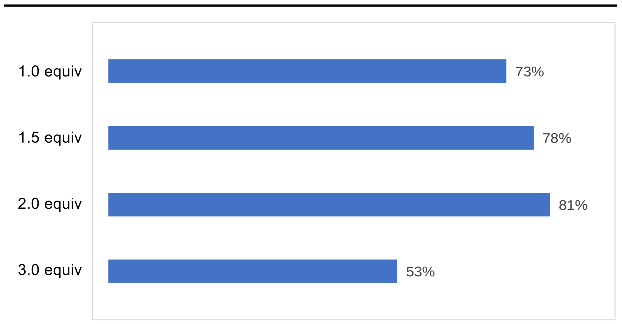

Ratio of acid to diazonium

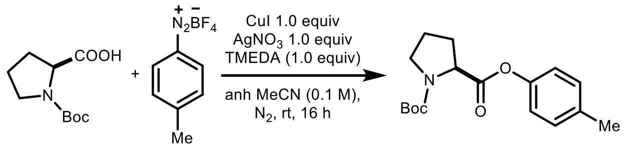

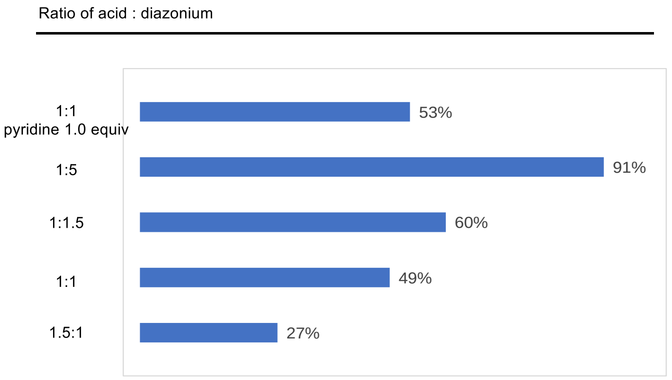

When utilizing catalytic CuI (20 mol%) and $AgNO_3$ (20 mol%), the NMR yield decreased to 12%. HTE campaigns in 96-well plates were subsequently carried out to optimize the conditions using copper catalysts. For subsequent optimization studies, *o*-toluic acid was used to facilitate use of ^{1}H-NMR analysis for yield determination.

Table S4 Reaction Duration and Pyridine Series

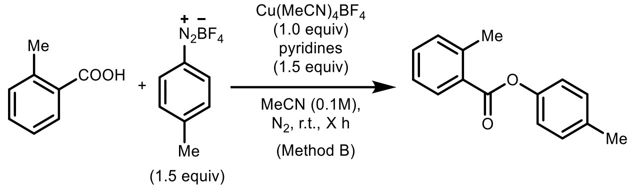

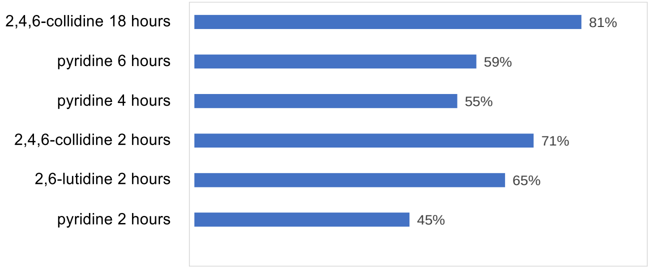

Table S5 Catalytic Loading of Cu(MeCN)4BF4

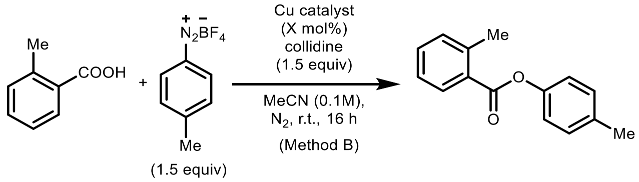

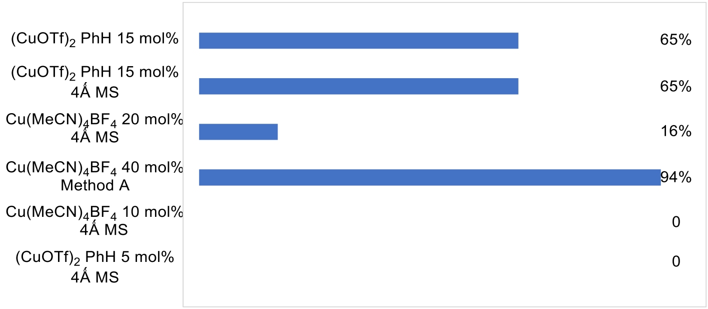

Select reactions identified in HTE screens in 96-well plates (Figure S2) were repeated on larger scale and the results are shown in Table S6 and S7.

Table S6 Ligand Screen

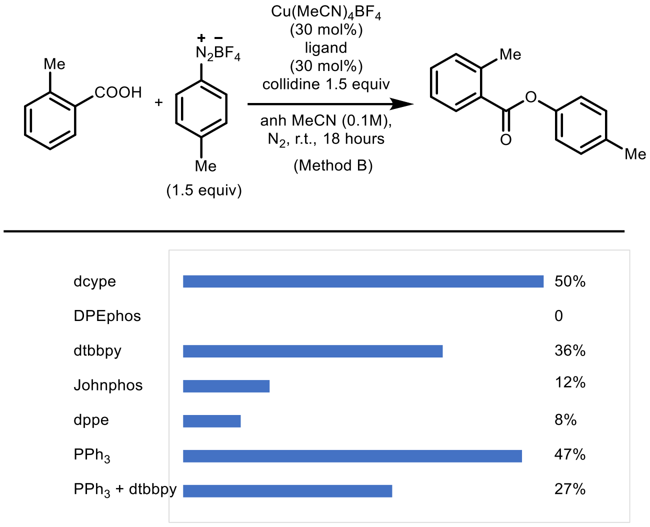

Table S7 Base Screen

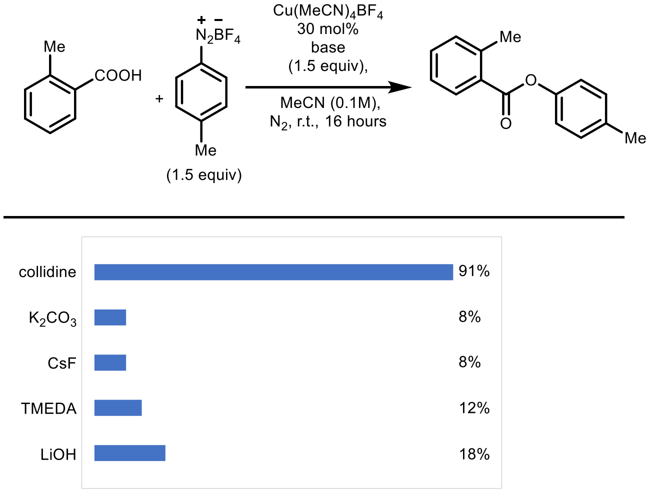

Method A. Under an atmosphere of dry nitrogen, a solution of carboxylic acid (0.100 mmol, 1.0 equiv) with anhydrous 2,4,6-collidine (0.150 mmol, 1.50 equiv) or other bases in anhydrous acetonitrile (0.300 mL) was prepared in a flame-dried 1-dram glass vial. Another solution of $Cu(MeCN)_4BF_4$ (0.030 mmol, 30 mol%), with or without ligands (0.045 mmol, 45 mol%), in anhydrous acetonitrile (0.40 mL) was prepared in another flame-dried 1-dram glass vial. A third solution of diazonium (0.150 mmol, 1.50 equiv) in anhydrous acetonitrile (0.30 mL), in a third flame-dried 1-dram glass vial was prepared. To the first acid and collidine solution was added dropwise via syringe the $Cu(MeCN)_4BF_4$ solution, concurrent with the dropwise addition of diazonium solution, or suspension. (Notes: The two solutions were added at the same time. CAUTION: the addition of diazonium solution generates bubbles and a reaction vessel should be of appropriate size and filled to that the reaction mixture occupies less than 25% of the vessel volume). The reaction mixture was sealed under nitrogen at room temperature and stirred until reaction completion as judged by TLC analysis. The crude reaction mixture was diluted with ethyl acetate (5.0 mL) and to the mixture, 1,3,5-trimethoxybenzene (0.030 mmol, 0.10 M in ethyl acetate stock solution freshly prepared upon the addition) was added as an internal standard, then the organic layer was washed with saturated aqueous sodium

sulfate solution (2 × 2.5 mL). (Notes: use sodium sulfate saturated solution instead of brine eliminated the formation of aryl chloride from excess arenediazonium salts). The organic layer was dried over anhydrous magnesium sulfate, filtered, followed by removal of the volatiles from the filtrate in vacuo. The ^{1}H-NMR of the crude was taken, and the NMR yield was determined according to the internal standard method described above.

Method B. Three solutions were prepared as in **Method A** but the timing of reagent addition was modified. The acid/collidine solution was prepared, then aged for 15 minutes, then the $Cu(MeCN)_4BF_4$ solution was added to the acid/collidine solution and this mixture stirred for 15 minutes. Finally the diazonium solution was added dropwise.

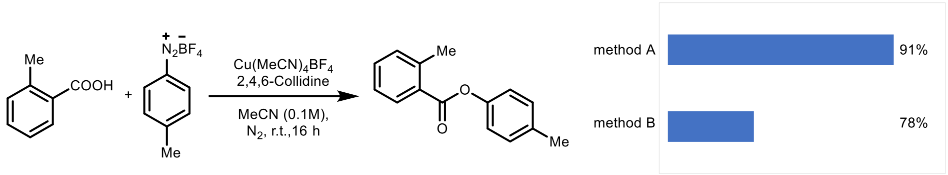

It was noted that applying **Method B** with a waiting time between the addition of reagents produced a decrease in the ^{1}H-NMR yield by 10%. Therefore, **Method A** was applied to the investigation of scale-up substrate studies.

Ultra-High Throughput Experimentation in 1,536-wellplate for the Investigation of Substrate Scope of Copper-catalyzed Esterification.

The reactions were prepared on 1.015 µL scale (15 nL benzonitrile was added between adding different reagents in each well to avoid cross contamination) 96 carboxylic acids (Table S8) were weighted out to oven-dried 1-dram vials and stored over calcium sulfate in a desiccator. The stock solutions of acids (0.408 M) were prepared with 2,4,6-collidine (0.612 M) in anhydrous and degassed benzonitrile in the glovebox with a 1.2× overage for each stock solution. The 96 solutions were generally clear solutions or evenly mixed slurries upon adding 2,4,6-collidine. A solution of $Cu(MeCN)_4BF_4$ in benzonitrile (0.408 M) was prepared and stirred in the glovebox until full solution occurred. Meanwhile, four diazonium stock solutions in benzonitrile (0.306 M) were prepared in oven-dried 2-dram vials. A deep well square 120 µL polypropylene 384-well plate (Analytical Sales parts No. 38120) was prepared as the source plate (Figure S4). The reagents from the source plate were dispensed to the 1,536-well reaction plate (Analytical Sales parts No. 15020) using an SPT Labtech mosquito® liquid handling robot inside the glovebox (Table S9).[1, 4] After dosing and mixing, the reaction plate was sealed with foil sealing tape and centrifuged, and then allowed to age at ambient temperature without further agitation for 20 hours inside the glovebox. After 20 hours, the foil sealing tape was removed, and the reactions were quenched by the addition of 1.015 µL of a 0.102 M DMSO solution of caffeine as internal standard to each reaction well. After mixing three times with the pipette tips using the mosquito®'s mixing function, the plate was then centrifuged again, and 1.000 µL of the quenched mixture from each well was transferred into four 384-well microtiter plates (Fisherbrand parts No. 12566615) containing 60.0 µL HPLC grade DMSO per well (pre-dosed by multi-pipettes) for analysis. The plates were then analyzed by UPLC-MS as described above, the results were processed and visualized as described in the Methods Summary (Figure S5). The output data from these ultra-HTE experiments were used to generate the heatmap in Figure S5 and are available in the Supplementary Data file 'HTE Data for Amine-Acid Esterification.xslx'. It was observed that 4 wells did not receive the dose of caffeine internal standard and are marked in grey color in the heatmap below (Figure S5).

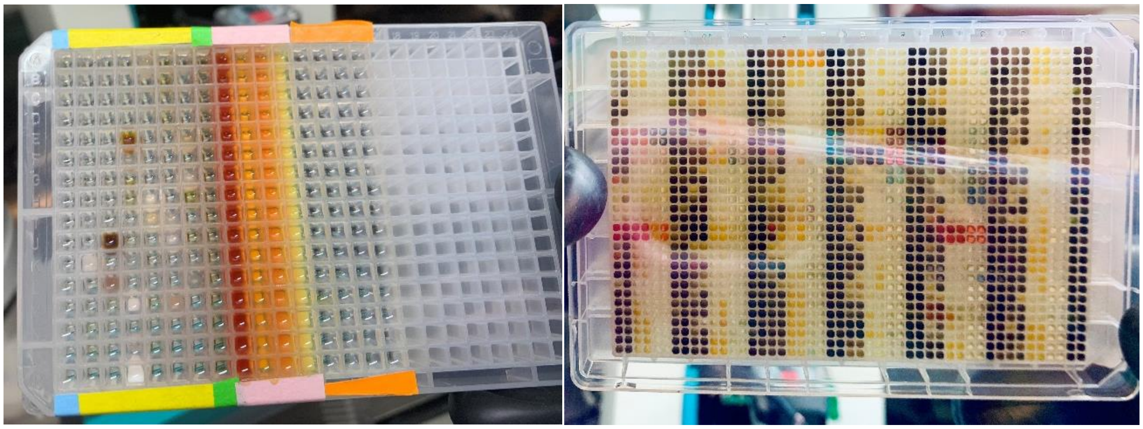

Figure S4 Reagents Dosed into 384-Wellplate as the Source Plate (left), and 1,536-Well Reaction Plate After 20 Hours (right).

Table S8 96 Carboxylic Acids Screened in the 1,536-ultraHTE Screen

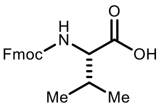
D1

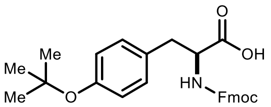
D2

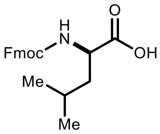
D3

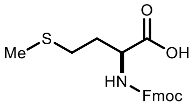
D4

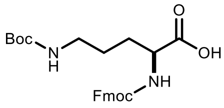
D5

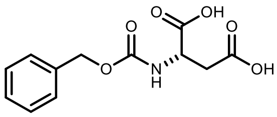
D6

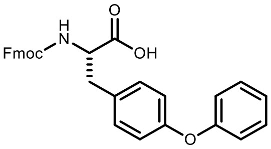
D7

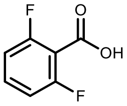
D8

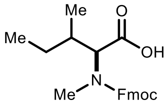
D9

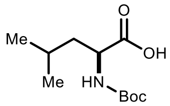
D10

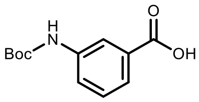
D11

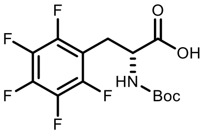
D12

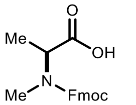
D13

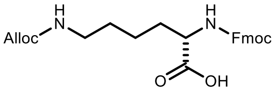
D14

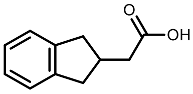
D15

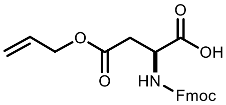
D16

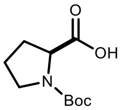
D17

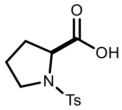
D18

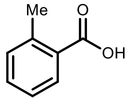
D19

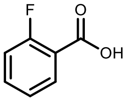
D20

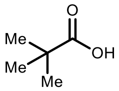
D21

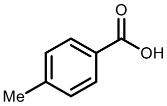
D22

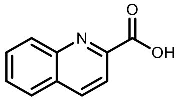
D23

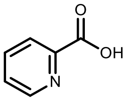
D24

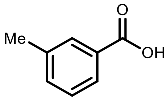
D25

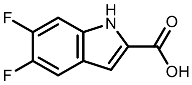
D26

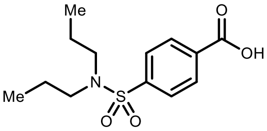
D27

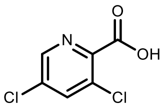
D28

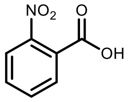
D29

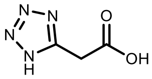
D30

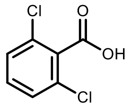
D31

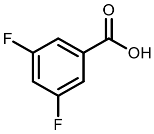
D32

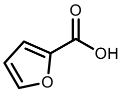
D33

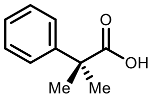
D34

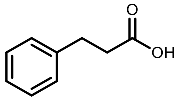
D35

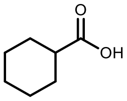
D36

Cont’d

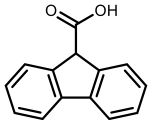
D37

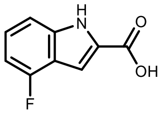
D38

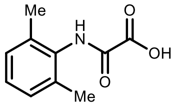
D39

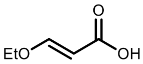
D40

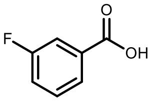
D41

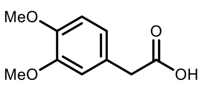
D42

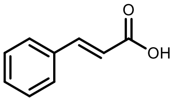
D43

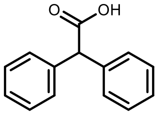
D44

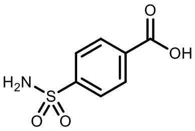
D45

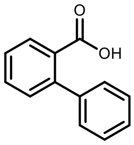
D46

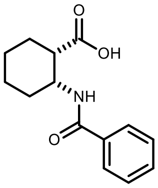
D47

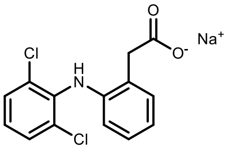
D48

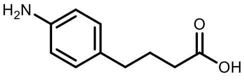
D49

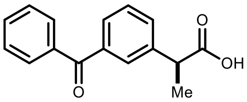
D50

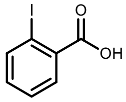
D51

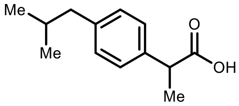
D52

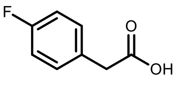
D53

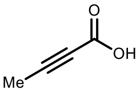
D54

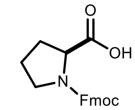
D55

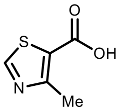
D56

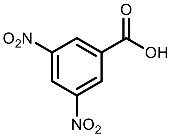
D57

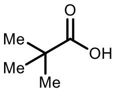
D58

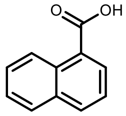
D59

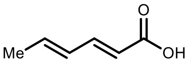
D60

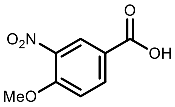
D61

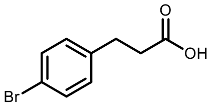
D62

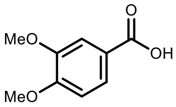
D63

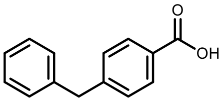
D64

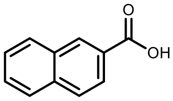
D65

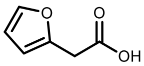
D66

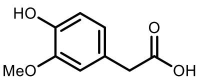
D67

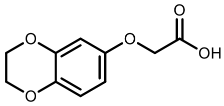
D68

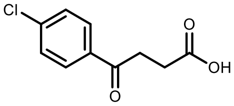
D69

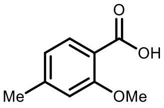
D70

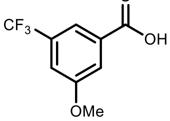
D71

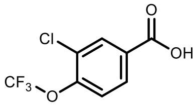
D72

Cont’d

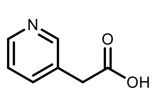

D73

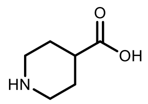

D74

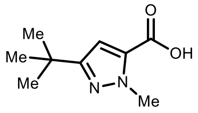

D75

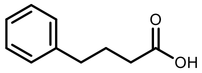

D76

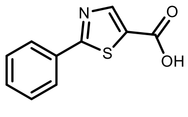

D77

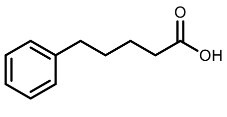

D78

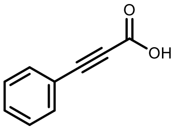

D79

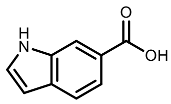

D80

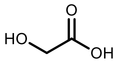

D81

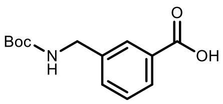

D82

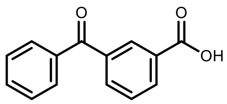

D83

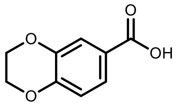

D84

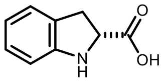

D85

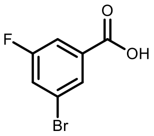

D86

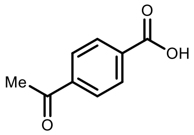

D87

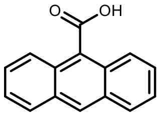

D88

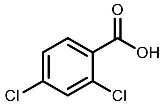

D89

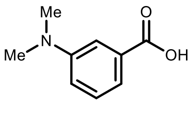

D90

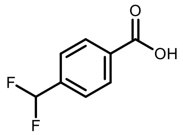

D91

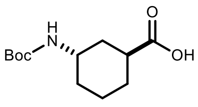

D92

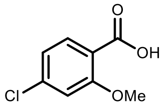

D93

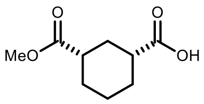

D94

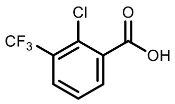

D95

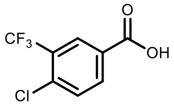

D96

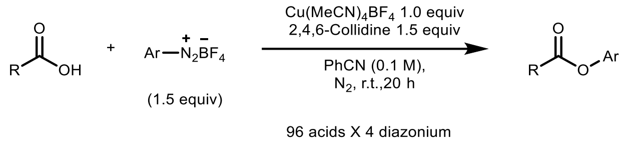

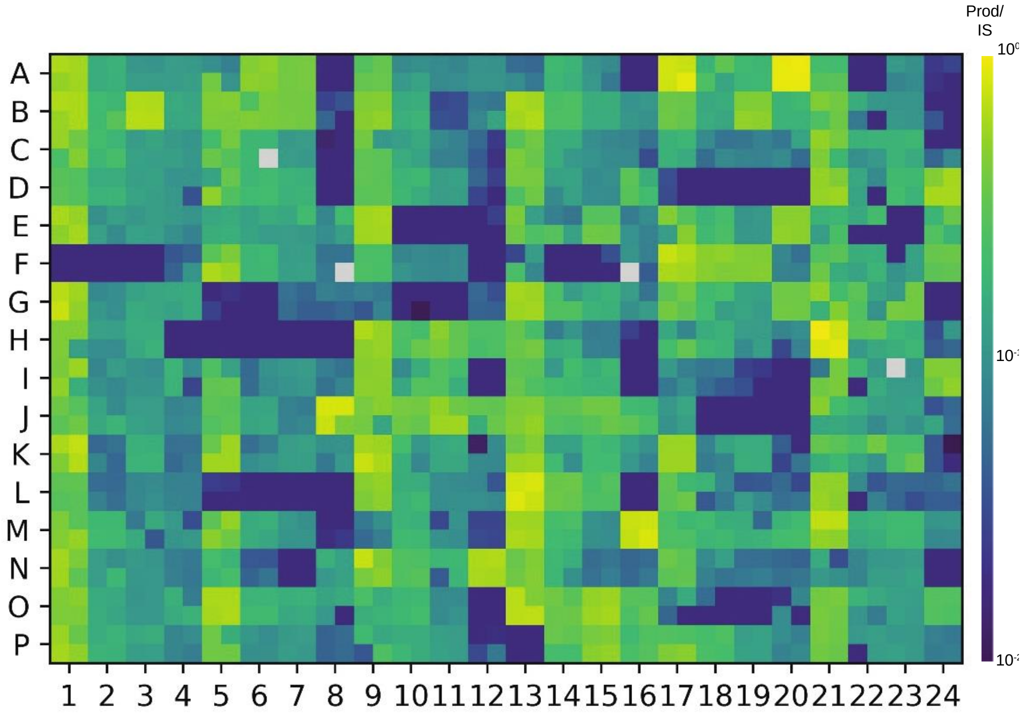

Figure S5 Visualization of ultraHTE in 1,536-Wellplate

Table S9 Reagents Dosed from the Source Plate to the Reaction Plate

**Reagents**	**C_{stock} (M)**	**V_{dose} (nL)**
Acid/collidine solution	0.408/0.612	250
Diazonium salts	0.306	500
$Cu(MeCN)_4BF_4$	0.408	250

We plotted the UV peak area obtained from the total wavelength chromatogram over that of the internal standard (1.0 equiv. caffeine vs. theoretical 100% yield). The peaks were identified by observation of their desired masses in the Virscidian Analytical Studio Pro software package. A threshold ratio was set at 2%, below which products were not reported. Note that a ratio of 2% does not necessarily indicate a low yield, as some products have low peak area due to low molar absorptivity by the UV detection method used. Overall, 322 out of 384 products were observed. The relative standard deviations of quadruplicate data for each substrate pair were calculated in python and the average for the relative standard deviation across the 384 reactions was determined to be 5.5% (Figure S6). Most of products retain later than caffeine in the LC readout, and we have selected representative reactions to show the different retention times. (Figure S7)

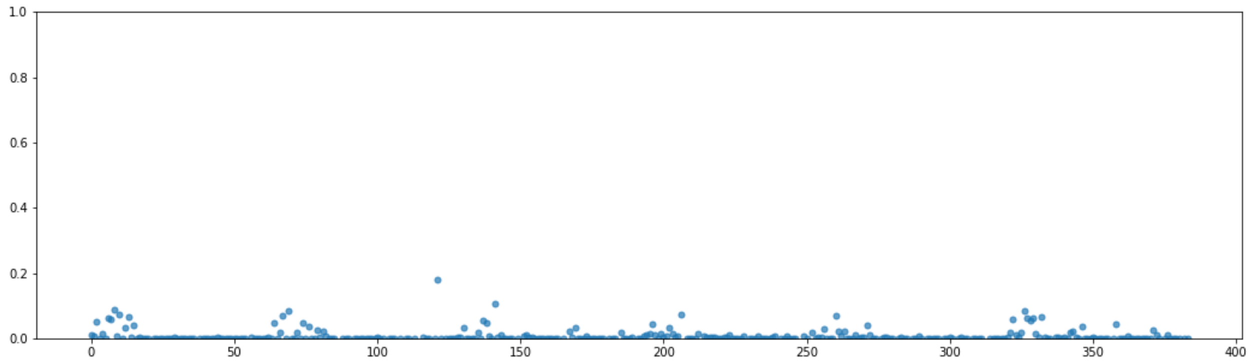

Figure S6 Relative Standard Deviation Plotted for 384 Reactions.

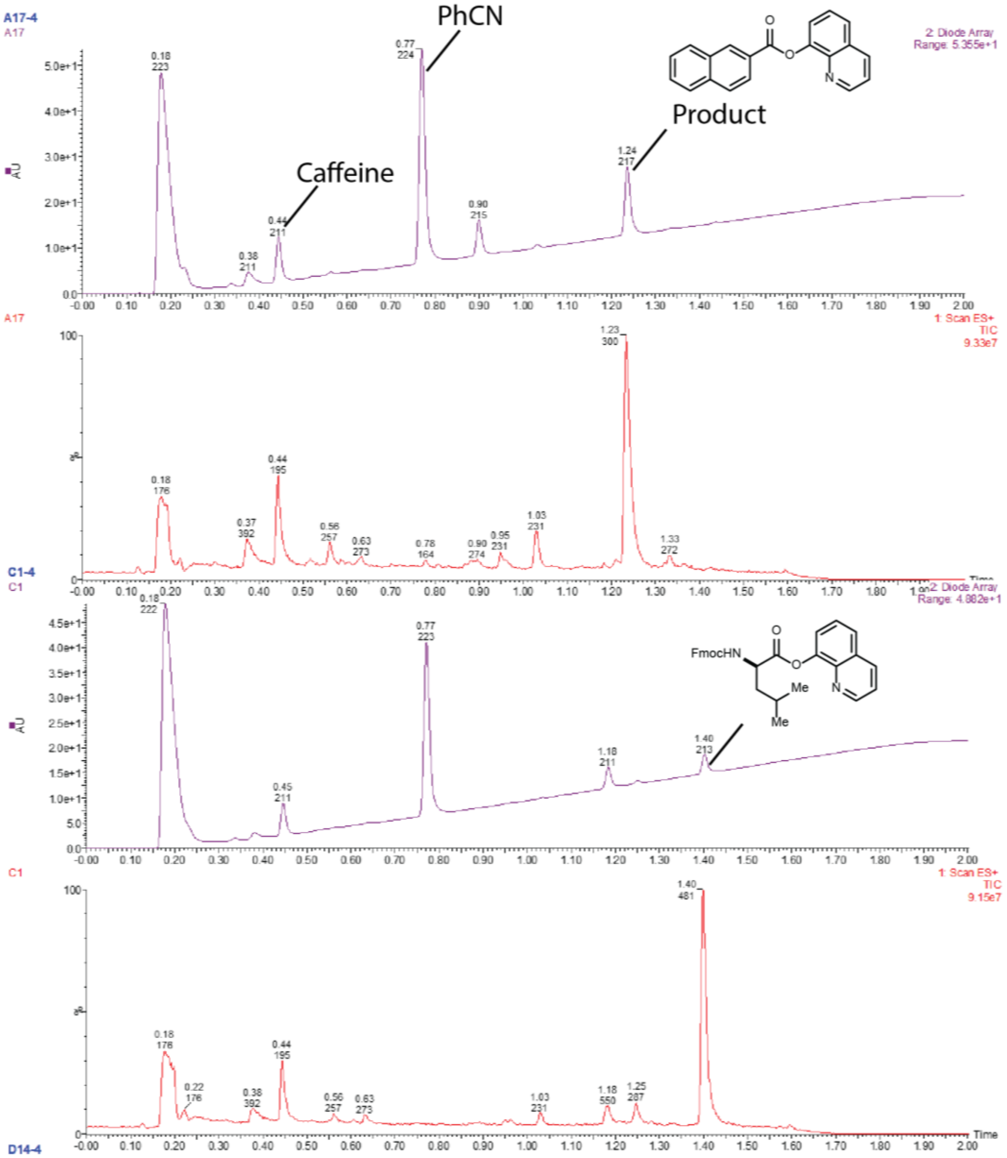

Figure S7 Representative UPLC Traces from the ultaHTE Experiment in a 1,536-Well Plate.

UV-Vis Absorbance Measurements

UV-Vis absorbance spectra were obtained for *o*-toluic acid (**4**, 0.100 M) in acetonitrile, *o*-toluic acid (**4**, 0.100 M) with $Cu(MeCN)_4BF_4$ (0.030 M) in acetonitrile, *o*-toluic acid (**4**, 0.100 M), $Cu(MeCN)_4BF_4$ (0.030 M) with 2,4,6-collidine (0.150 M) in acetonitrile, *o*-toluic acid (**4**, 0.100 M) with 2,4,6-collidine (0.150 M) in acetonitrile, $Cu(MeCN)_4BF_4$ (0.030 M) with 2,4,6-collidine (0.150 M) in acetonitrile, and *o*-toluic acid (**4**, 0.100 M) with diazonium salt (**5**, 0.150 M) in acetonitrile respectively, using the method as described above in Methods Summary. The plots were obtained using the average of three readouts for each solution, the visualization was done using python (Figure S8).

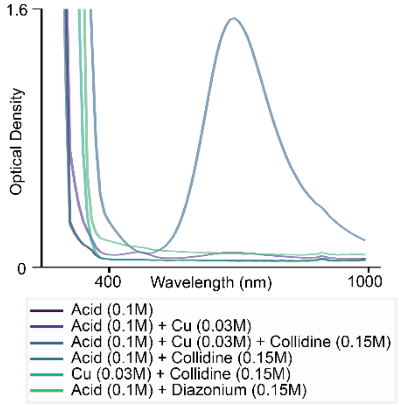

Figure S8 UV-Vis absorbance spectra

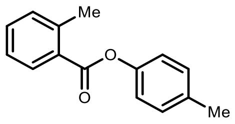

6

p-Tolyl 2-methylbenzoate (6)

The ester **6** was prepared according to **Method A** on a 0.300 mmol scale. After sixteen hours, the reaction mixture was diluted with 10 mL ethyl acetate and washed with saturated sodium sulfate aqueous solution (2 × 10 mL). The organic layer was dried over anhydrous magnesium sulfate, filtered, followed by removal of the volatiles from the filtrate in vacuo.

Purification by flash chromatography on silica gel with gradient of hexanes to 20% dichloromethane in hexanes to give the product **6** (60.3 mg, 89%, 72% isolated following *in situ* protocol).

Colorless oil, R_f = 0.62 (ethyl acetate / hexanes 1:20).

^{1}H NMR (700 MHz, $CDCl_3$): δ 8.16 (dd, *J* = 7.5, 1.9 Hz, 1H), 7.48 (td, *J* = 7.5, 1.5 Hz, 1H), 7.33 (t, *J* = 7.8 Hz, 2H), 7.23 (d, *J* = 8.3 Hz, 2H), 7.13 – 7.04 (m, 2H), 2.68 (s, 3H), 2.38 (s, 3H).

^{13}C NMR (176 MHz, $CDCl_3$): δ 165.80, 148.49, 141.03, 135.26, 132.75, 132.06, 131.26, 130.12, 128.86, 126.02, 121.62, 22.07, 20.73.

HRMS (APCI+): calculated $C_{15}H_{15}O_2$ $[M+H]^+$: 227.1067, found: 227.1065.

in-situ Diazotization Protocol of Esterification Reaction

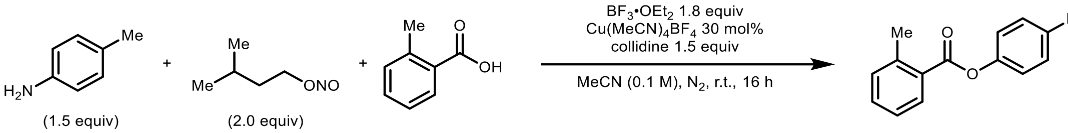

The *in-situ* diazotization procedure was designed based on reported work.[5] A flame-dried 2 dram vial was charged with *p*-toluidine (0.450 mmol, 1.5 equiv) and anhydrous acetonitrile (0.90 mL) under nitrogen. Boron trifluoride etherate (BF_3•OEt_2, 0.540 mmol, 1.8 equiv) was added via micro syringe, followed by isopentyl nitrite (^{i}AmONO, 0.600 mmol, 2.0 equiv). The resulting solution was aged at ambient temperature for 20 min, at which point full consumption of aniline was determined by TLC. *O*-toluic acid (0.300 mmol, 1.0 equiv) with 2,4,6-collidine (0.450 mmol, 1.5 equiv) were then added to a second flame-dried vial under nitrogen with anhydrous acetonitrile (1.0 mL). Meanwhile, a $Cu(MeCN)_4BF_4$ solution in acetonitrile (0.90 mL) was prepared in a third vial (0.090 mmol, 30 mol%). The *in situ*-formed diazonium solution was then added to the reaction vessel dropwise via syringe, concurrent with the addition of the $Cu(MeCN)_4BF_4$ solution. The reaction was then stirred at room temperature for 16 hours. The reaction mixture was then diluted with ethyl acetate (10 mL) and washed with an aqueous solution of saturated sodium sulfate (2 × 10 mL). The organic layer was dried over anhydrous magnesium sulfate, filtered, and volatiles were removed *in vacuo*. Purification of the ester **6** was performed using flash column chromatography on silica gel giving the desired product in 72% isolated yield (48.8 mg).

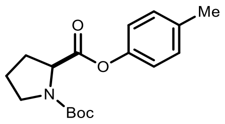

15

1-(*tert*-butyl) 2-(*p*-tolyl) (*S*)-Pyrrolidine-1,2-dicarboxylate (15)

The ester **15** was prepared using method A above in a 0.300 mmol scale. After sixteen hours, the reaction mixture was diluted with 10 mL ethyl acetate and washed with saturated sodium sulfate aqueous solution (2 $\times$ 10 mL). The organic layer was dried over anhydrous magnesium sulfate, filtered, followed by removal of the volatiles from the filtrate in vacuo.

Purification by flash chromatography on silica gel with gradient of 5% to 15% ethyl acetate in hexanes to give the ester **15** (56.0 mg, 61%).

White solid, R_f = 0.39 (20% ethyl acetate in hexanes).

^{1}H NMR (700 MHz, $CDCl_3$): δ 7.17 (d, *J* = 8.0 Hz, 2H, major rotamer), 7.14 (d, *J* = 8.0 Hz, 2H, minor), 6.99 (d, *J* = 8.1 Hz, 2H, minor), 6.96 (d, *J* = 8.1 Hz, 2H, major), 4.51 (dd, *J* = 8.7, 4.1 Hz, minor), 4.43 (dd, *J* = 8.7, 4.2 Hz, 1H, major), 3.64 – 3.60 (m, 1H, major), 3.58 – 3.54 (m, 1H, minor), 3.54 – 3.49 (m, 1H, major), 3.46 – 3.41 (m, 1H, minor), 2.40 – 2.28 (m, 1H, major + minor), 2.34 (s, 3H, major), 2.32 (s, 3H, minor), 2.19 – 2.12 (m, 1H, major + minor), 2.03 (m, 1H, major + minor), 1.97 – 1.89 (m, 1H, major + minor), 1.47 (s, 9H, minor), 1.46 (s, 9H, major).

^{13}C NMR (176 MHz, $CDCl_3$): δ 171.88 (major + minor), 154.54 (minor), 153.89 (major), 148.67 (minor), 148.49 (major), 135.68 (major), 135.54 (minor), 130.08 (major), 129.93 (minor), 121.24 (minor), 120.92 (major), 80.27 (major), 80.00 (minor), 59.30 (major), 59.19 (minor), 46.73 (minor), 46.55 (major), 31.17 (major), 30.13 (minor), 28.54 (major + minor), 24.59 (minor), 23.81 (major), 20.97 (major + minor).

HRMS (ESI+): calculated $C_{17}H_{23}NNaO_4^+$ $[M+Na]^+$: 328.1519, found: 328.1517.

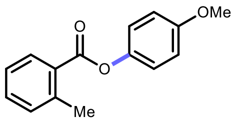

16

4-Methoxyphenyl 2-methylbenzoate (**16**)

The ester **16** was prepared using **method A** above in a 0.300 mmol scale. After sixteen hours, the reaction mixture was diluted with 10 mL ethyl acetate and washed with saturated sodium sulfate aqueous solution (2 × 10 mL). The organic layer was dried over anhydrous magnesium sulfate, filtered, followed by removal of the volatiles from the filtrate in vacuo.

Purification by flash chromatography on silica gel with gradient of 10% to 25% dichloromethane in hexanes to give the ester **16** (64.7 mg, 89%).

White solid, R_f = 0.44 (10% ethyl acetate in hexanes)

^{1}H NMR (700 MHz, $CDCl_3$): δ 8.15 (dd, *J* = 7.8, 1.4 Hz, 1H), 7.48 (ddd, *J* = 7.5, 1.5 Hz, 1H), 7.34 – 7.30 (m, 2H), 7.14 – 7.12 (m, 2H), 6.96 – 6.94 (m, 2H), 3.83 (s, 3H), 2.67 (s, 3H).

^{13}C NMR (176 MHz, $CDCl_3$): δ 166.21, 157.27, 144.37, 141.21, 132.61, 131.91, 131.10, 128.66, 125.87, 122.54, 114.53, 55.63, 21.93.

HRMS (APCI+): calculated $C_{15}H_{15}O_3$+ $[M+H]^+$: 243.1016, found: 243.1017.

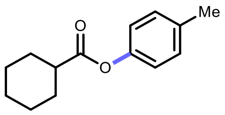

17

***p*-Tolyl cyclohexanecarboxylate (17)**

The ester **17** was prepared using **method A** above in a 0.300 mmol scale. After sixteen hours, the reaction mixture was diluted with 10 mL ethyl acetate and washed with saturated sodium sulfate aqueous solution (2 × 10 mL). The organic layer was dried over anhydrous magnesium sulfate, filtered, followed by removal of the volatiles from the filtrate in vacuo.

Purification by flash chromatography on silica gel with gradient of 5% to 15% ethyl acetate in hexanes to give the ester **17** (57.0 mg, 87%).

White solid, R_f = 0.40 (10% ethyl acetate in hexanes)

^{1}H NMR (700 MHz, $CDCl_3$): δ 7.17 (d, *J* = 7.7 Hz, 2H), 6.95 (ddd, 2H), 2.58 – 2.53 (m, 1H), 2.35 (s, 3H), 2.07 (dp, *J* = 13.4, 3.5 Hz, 2H), 1.83 (dp, *J* = 11.1, 3.7 Hz, 2H), 1.72 – 1.67 (m, 1H), 1.60 (qd, *J* = 12.9, 12.3, 3.5 Hz, 2H), 1.40 – 1.26 (m, 3H).

^{13}C NMR (176 MHz, $CDCl_3$): δ 174.83, 148.76, 135.29, 129.96, 121.33, 43.33, 29.10, 25.87, 25.51, 20.97.

HRMS (APCI+): calculated $C_{14}H_{19}O_2^+$ $[M+H]^+$: 219.1380, found: 219.1385.

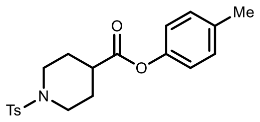

18

***p*-Tolyl 1-tosylpiperidine-4-carboxylate (18)**

The ester **18** was prepared using method A above in a 0.300 mmol scale. After sixteen hours, the reaction mixture was diluted with 10 mL ethyl acetate and washed with saturated sodium sulfate aqueous solution (2 × 10 mL). The organic layer was dried over anhydrous magnesium sulfate, filtered, followed by removal of the volatiles from the filtrate in vacuo.

Purification by flash chromatography on silica gel with gradient of 5% to 15% ethyl acetate in hexanes to give the product **18** (93.9 mg, 84%).

White solid, R_f = 0.28 (20% ethyl acetate in hexanes).

^{1}H NMR (500 MHz, $CDCl_3$): δ 7.66 (d, *J* = 8.2 Hz, 2H), 7.33 (d, *J* = 8.0 Hz, 2H), 7.14 (d, *J* = 8.4 Hz, 2H), 6.91 – 6.85 (m, 2H), 3.68 – 3.61 (m, 2H), 2.61 – 2.48 (m, 3H), 2.44 (s, 3H), 2.32 (s, 3H), 2.10 (dt, *J* = 13.2, 3.9 Hz, 2H), 1.96 (dtd, *J* = 14.1, 10.5, 3.9 Hz, 2H).

^{13}C NMR (176 MHz, $CDCl_3$): δ 172.68, 148.33, 143.76, 135.71, 133.22, 130.05, 129.8, 127.80, 121.08, 45.39, 40.13, 27.55, 21.65, 20.95.

HRMS (ESI+): calculated $C_{20}H_{23}NNaO_4S^+$ $[M+Na]^+$: 396.1240, found: 396.1236.

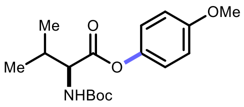

19

4-Methoxyphenyl (tert-butoxycarbonyl)-*L*-valinate (19)

The ester **19** was prepared using **method A** above in a 0.300 mmol scale. After sixteen hours, the reaction mixture was diluted with 10 mL ethyl acetate and washed with saturated sodium sulfate aqueous solution (2 × 10 mL). The organic layer was dried over anhydrous magnesium sulfate, filtered, followed by removal of the volatiles from the filtrate in vacuo.

Purification by flash chromatography on silica gel with gradient of 5% to 15% ethyl acetate in hexanes to give the product **1** (80.5 mg, 83%).

Slightly yellow solid, R_f = 0.42 (20% ethyl acetate in hexanes).

^{1}H NMR (700 MHz, $CDCl_3$): δ 7.02 – 6.98 (m, 2H), 6.88 (d, *J* = 9.0 Hz, 2H), 5.09 (d, *J* = 9.1 Hz, 1H), 4.45 (dd, *J* = 9.2, 4.8 Hz, 1H), 3.79 (s, 3H), 2.32 (m, 1H), 1.46 (s, 9H), 1.07 (d, *J* = 6.8 Hz, 3H), 1.01 (d, *J* = 6.9 Hz, 3H).

^{13}C NMR (176 MHz, $CDCl_3$): δ 171.56, 157.53, 155.83, 144.04, 122.25, 114.62, 80.06, 58.76, 55.71, 31.55, 28.46, 19.21, 17.80.

HRMS (ESI+): calculated $C_{17}H_{25}NNaO_5^+$ $[M+Na]^+$: 346.1625, found: 346.1617.

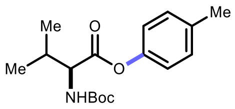

20

4-Methylphenyl (*tert*-butoxycarbonyl)-*L*-valinate (**20**)

The ester **20** was prepared using **method A** above in a 0.300 mmol scale. After sixteen hours, the reaction mixture was diluted with 10 mL ethyl acetate and washed with saturated sodium sulfate aqueous solution (2 × 10 mL). The organic layer was dried over anhydrous magnesium sulfate, filtered, followed by removal of the volatiles from the filtrate in vacuo.

Purification by flash chromatography on silica gel with gradient of 5% to 15% ethyl acetate in hexanes to give the product **20** (73.8 mg, 80%).

White solid, R_f = 0.61 (20% ethyl acetate in hexanes).

^{1}H NMR (700 MHz, $CDCl_3$): δ 7.17 (d, *J* = 8.1 Hz, 2H), 6.97 (m, 2H), 5.09 (d, *J* = 9.1 Hz, 1H), 4.46 (dd, *J* = 9.2, 4.7 Hz, 1H), 2.34 (s, 3H), 1.47 (s, 9H), 1.08 (d, *J* = 6.9 Hz, 3H), 1.02 (d, *J* = 6.9 Hz, 3H).

^{13}C NMR (176 MHz, $CDCl_3$): δ 171.40, 155.85, 148.34, 135.87, 130.12, 121.18, 80.08, 58.78, 31.59, 28.47, 21.01, 19.23, 17.79.

HRMS (ESI+): calculated $C_{17}H_{25}NNaO_4^+$ $[M+Na]^+$: 330.1676, found: 330.1669.

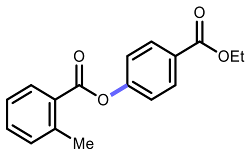

21

4-(Ethoxycarbonyl)phenyl 2-methylbenzoate (21)

The ester **21** was prepared using **method A** above in a 0.300 mmol scale. After sixteen hours, the reaction mixture was diluted with 10 mL ethyl acetate and washed with saturated sodium sulfate aqueous solution (2 × 10 mL). The organic layer was dried over anhydrous magnesium sulfate, filtered, followed by removal of the volatiles from the filtrate in vacuo.

Purification by flash chromatography on silica gel with gradient of 5% to 15% ethyl acetate in hexanes to give the product **21** (63.1 mg, 74%).

White solid, R_f = 0.59 (20% ethyl acetate in hexanes).

^{1}H NMR (500 MHz, $CDCl_3$): δ 8.17 (dd, *J* = 7.4, 1.6 Hz, 1H), 8.15 – 8.12 (m, 2H), 7.50 (ddd, *J* = 7.5, 1.5 Hz, 1H), 7.36 – 7.32 (m, 2H), 7.31 – 7.28 (m, 2H), 4.40 (q, *J* = 7.1 Hz, 2H), 2.68 (s, 3H), 1.41 (t, *J* = 7.1 Hz, 3H).

^{13}C NMR (126 MHz, $CDCl_3$): δ 166.01, 165.29, 154.67, 141.71, 133.15, 132.20, 131.38, 131.30, 128.19, 126.13, 121.95, 121.94, 61.22, 22.11, 14.49.

HRMS (APCI+) calculated $C_{17}H_{17}O_4^+$ $[M+H]^+$: 285.1121, found: 285.1117.

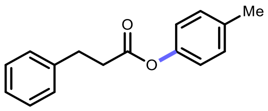

22

p-Tolyl 3-phenylpropanoate (22)

The ester **22** was prepared using **method A** above in a 0.300 mmol scale. After sixteen hours, the reaction mixture was diluted with 10 mL ethyl acetate and washed with saturated sodium sulfate aqueous solution (2 × 10 mL). The organic layer was dried over anhydrous magnesium sulfate, filtered, followed by removal of the volatiles from the filtrate in vacuo.

Purification by flash chromatography on silica gel with gradient of 5% to 15% ethyl acetate in hexanes to give the product **22** (56.2 mg, 78%).

Clear liquid, R_f = 0.66 (20% ethyl acetate in hexanes).

^{1}H NMR (500 MHz, $CDCl_3$): δ 7.40 – 7.26 (m, 5H), 7.19 (d, *J* = 8.1 Hz, 2H), 6.94 (d, *J* = 8.3 Hz, 2H), 3.12 (t, *J* = 7.7 Hz, 2H), 2.91 (t, *J* = 7.7 Hz, 2H), 2.37 (s, 3H).

^{13}C NMR (126 MHz, $CDCl_3$): δ 171.65, 148.52, 140.28, 135.48, 129.99, 128.66, 128.50, 126.51, 121.28, 36.09, 31.09, 20.94.

HRMS (APCI+): calculated $C_{16}H_{17}O_2^+$ [M+H]$^+$: 241.1223, found: 241.1222.

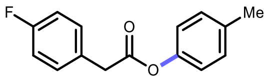

23

***p*-Tolyl 2-(4-fluorophenyl)acetate (23)**

The ester **23** was prepared using **method A** above in a 0.300 mmol scale. After sixteen hours, the reaction mixture was diluted with 10 mL ethyl acetate and washed with saturated sodium sulfate aqueous solution (2 × 10 mL). The organic layer was dried over anhydrous magnesium sulfate, filtered, followed by removal of the volatiles from the filtrate in vacuo.

Purification by flash chromatography on silica gel with gradient of 5% to 15% ethyl acetate in hexanes to give the product **23** (57.2 mg, 78%).

Slightly yellow solid, R_f = 0.54 (10% ethyl acetate in hexanes).

^{1}H NMR (500 MHz, $CDCl_3$): δ 7.41 – 7.33 (m, 2H), 7.18 (d, *J* = 8.2 Hz, 2H), 7.12 – 7.03 (m, 2H), 6.99 – 6.94 (m, 2H), 3.84 (s, 2H), 2.36 (s, 3H).

^{13}C NMR (126 MHz, $CDCl_3$): δ 170.12 (d, *J* = 1.4 Hz), 162.23 (d, *J* = 245.6 Hz), 148.54, 135.66, 131.00 (d, *J* = 8.1 Hz), 130.01, 129.37 (d, *J* = 3.3 Hz), 121.14, 115.65 (d, *J* = 21.5 Hz), 40.59, 20.92.

HRMS (APCI+): calculated $C_{15}H_{14}FO_2^+$ $[M+H]^+$: 245.0972, found: 245.0973.

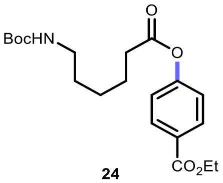

24

Ethyl 4-((6-((*tert*-butoxycarbonyl)amino)hexanoyl)oxy)benzoate (24)

The ester **24** was prepared using **method A** above in a 0.300 mmol scale. After sixteen hours, the reaction mixture was diluted with 10 mL ethyl acetate and washed with saturated sodium sulfate aqueous solution (2 × 10 mL). The organic layer was dried over anhydrous magnesium sulfate, filtered, followed by removal of the volatiles from the filtrate in vacuo.

Purification by flash chromatography on silica gel with gradient of 5% to 15% ethyl acetate in hexanes to give the product **24** (86.5 mg, 76%).

White solid, R_f = 0.4 (20% ethyl acetate in hexanes).

^{1}H NMR (700 MHz, $CDCl_3$): δ 8.08 – 8.03 (m, 2H), 7.16 – 7.11 (m, 2H), 4.59 (s, 1H), 4.36 (q, *J* = 7.1 Hz, 2H), 3.13 (d, *J* = 6.8 Hz, 2H), 2.56 (t, *J* = 7.5 Hz, 2H), 1.79 – 1.72 (m, 2H), 1.57 – 1.41 (m, 2H), 1.43 (s, 9H), 1.43 (m, 2H), 1.37 (t, *J* = 7.1 Hz, 3H).

^{13}C NMR (176 MHz, $CDCl_3$): δ 171.57, 165.91, 156.09, 154.36, 131.18, 128.08, 121.62, 79.20, 61.16, 40.44, 34.32, 29.89, 28.53, 26.34, 24.55, 14.42.

HRMS (ESI+): calculated $C_{20}H_{29}NNaO_6^+$ $[M+Na]^+$: 402.1887, found: 402.1884.

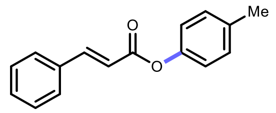

25

p-Tolyl cinnamate (25)

The ester **25** was prepared using **method A** above in a 0.300 mmol scale. After sixteen hours, the reaction mixture was diluted with 10 mL ethyl acetate and washed with saturated sodium sulfate aqueous solution (2 × 10 mL). The organic layer was dried over anhydrous magnesium sulfate, filtered, followed by removal of the volatiles from the filtrate in vacuo.

Purification by flash chromatography on silica gel with gradient of 5% to 15% ethyl acetate in hexanes to give the product **25** (51.5 mg, 72%).

White solid, R_f = 0.2 (ethyl acetate / hexanes 1:20).

^{1}H NMR (700 MHz, $CDCl_3$): δ 7.87 (d, *J* = 16.0 Hz, 1H), 7.61 – 7.57 (m, 2H), 7.44 – 7.41 (m, 3H), 7.21 (d, *J* = 7.8 Hz, 2H), 7.08 – 7.04 (m, 2H), 6.64 (d, *J* = 16.0 Hz, 1H), 2.37 (s, 3H).

^{13}C NMR (176 MHz, $CDCl_3$): δ 165.75, 148.69, 146.52, 135.56, 134.37, 130.78, 130.09, 129.12, 128.41, 121.43, 117.57, 21.04.

HRMS (APCI+): calculated: $C_{16}H_{15}O_2^+$ [M +H]$^+$: 239.1067, found: 239.1067.

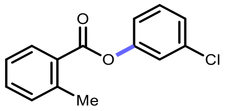

26

3-Chlorophenyl 2-methylbenzoate (26)

The ester **26** was prepared using **method A** above in a 0.300 mmol scale. After sixteen hours, the reaction mixture was diluted with 10 mL ethyl acetate and washed with saturated sodium sulfate aqueous solution (2 × 10 mL). The organic layer was dried over anhydrous magnesium sulfate, filtered, followed by removal of the volatiles from the filtrate in vacuo.

Purification by flash chromatography on silica gel with gradient of hexanes to 20% dichloromethane in hexanes to give the product **26** (51.8 mg, 70%).

Slightly yellow liquid, R_f = 0.38 (ethyl acetate / hexanes 1:20).

^{1}H NMR (700 MHz, $CDCl_3$): δ 8.14 (dd, *J* = 7.8, 1.5 Hz, 1H), 7.51 – 7.48 (m, 1H), 7.38 – 7.35 (m, 1H), 7.33 (t, *J* = 7.8 Hz, 2H), 7.28 – 7.25 (m, 2H), 7.14 – 7.12 (m, 1H), 2.67 (s, 3H).

^{13}C NMR (176 MHz, $CDCl_3$): δ 165.46, 151.56, 141.70, 134.91, 133.14, 132.20, 131.35, 130.34, 128.16, 126.26, 126.13, 122.70, 120.42, 22.12.

HRMS (APCI+): calculated: $C_{14}H_{12}ClO_2^+$ $[M+H]^+$: 247.0520, found: 247.0526.

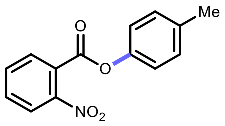

27

_p_-Tolyl 2-nitrobenzoate (27)

The ester **27** was prepared using **method A** above in a 0.300 mmol scale. After sixteen hours, the reaction mixture was diluted with 10 mL ethyl acetate and washed with saturated sodium sulfate aqueous solution (2 × 10 mL). The organic layer was dried over anhydrous magnesium sulfate, filtered, followed by removal of the volatiles from the filtrate in vacuo.

Purification by flash chromatography on silica gel with gradient of 5% to 15% ethyl acetate in hexanes to give the product **27** (53.2 mg, 69%).

White solid, R_f = 0.38 (20% ethyl acetate in hexanes).

^{1}H NMR (500 MHz, $CDCl_3$): δ 8.03 (d, *J* = 8.0 Hz, 1H), 7.88 (d, *J* = 7.5 Hz, 1H), 7.76 (t, *J* = 7.6 Hz, 1H), 7.70 (t, *J* = 7.7 Hz, 1H), 7.23 (d, *J* = 8.1 Hz, 2H), 7.14 (d, *J* = 8.6 Hz, 2H), 2.37 (s, 3H).

^{13}C NMR (126 MHz, $CDCl_3$): δ 164.52, 148.37, 148.22, 136.31, 133.36, 132.15, 130.25, 130.22, 127.76, 124.32, 121.03, 21.07.

HRMS (APCI+): calculated: $C_{14}H_{12}NO_4^+$ $[M+H]^+$: 258.0761, found: 258.0765.

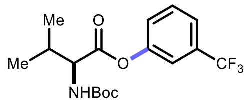

28

3-(Trifluoromethyl)phenyl (*tert*-butoxycarbonyl)-*L*-valinate (28)

The ester **28** was prepared using **method A** above in a 0.300 mmol scale. After sixteen hours, the reaction mixture was diluted with 10 mL ethyl acetate and washed with saturated sodium sulfate aqueous solution (2 × 10 mL). The organic layer was dried over anhydrous magnesium sulfate, filtered, followed by removal of the volatiles from the filtrate in vacuo.

Purification by flash chromatography on silica gel with gradient of 5% to 15% ethyl acetate in hexanes to give the product **28** (69.4 mg, 64%).

White solid, R_f = 0.55 (20% ethyl acetate in hexanes).

^{1}H NMR (500 MHz, $CDCl_3$): δ 7.52 – 7.49 (m, 2H), 7.38 – 7.35 (m, 1H), 7.30 (td, *J* = 4.9, 2.5 Hz, 1H), 5.06 (d, *J* = 9.0 Hz, 1H), 4.46 (dd, *J* = 9.0, 4.9 Hz, 1H), 2.33 (dq, *J* = 13.4, 6.8 Hz, 1H), 1.47 (s, 9H), 1.09 (d, *J* = 6.9 Hz, 3H), 1.03 (d, *J* = 6.9 Hz, 3H).

^{13}C NMR (126 MHz, $CDCl_3$): δ 170.96, 155.85, 150.65, 132.20 (q, *J* = 32.8 Hz), 130.23, 125.19, 123.04 (q, *J* = 3.8 Hz), 124.66 (d, *J* = 272.3 Hz), 118.84 (q, *J* = 3.4 Hz), 80.34, 58.95, 31.34, 28.44, 19.27, 17.88.

HRMS (ESI+): calculated: $C_{17}H_{22}F_3NNaO_4^+$ $[M+Na]^+$: 384.1393, found: 384.1391.

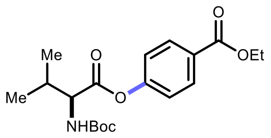

29

Ethyl 4-(((*tert*-butoxycarbonyl)-*L*-valyl)oxy)benzoate (29)

The ester **29** was prepared using **method A** above in a 0.300 mmol scale. After sixteen hours, the reaction mixture was diluted with 10 mL ethyl acetate and washed with saturated sodium sulfate aqueous solution (2 × 10 mL). The organic layer was dried over anhydrous magnesium sulfate, filtered, followed by removal of the volatiles from the filtrate in vacuo.

Purification by flash chromatography on silica gel with gradient of 5% to 15% ethyl acetate in hexanes to give the product **29** (68.0 mg, 62%).

White solid, R_f = 0.45 (20% ethyl acetate in hexanes).

^{1}H NMR (500 MHz, $CDCl_3$): δ 7.52 – 7.49 (m, 2H), 7.38 – 7.35 (m, 1H), 7.30 (td, *J* = 4.9, 2.5 Hz, 1H), 5.06 (d, *J* = 9.0 Hz, 1H), 4.46 (dd, *J* = 9.0, 4.9 Hz, 1H), 2.33 (dq, *J* = 13.4, 6.8 Hz, 1H), 1.47 (s, 9H), 1.09 (d, *J* = 6.9 Hz, 3H), 1.03 (d, *J* = 6.9 Hz, 3H).

^{13}C NMR (126 MHz, $CDCl_3$): δ 170.96, 155.85, 150.65, 132.20 (q, *J* = 32.8 Hz), 130.23, 125.19, 123.04 (q, *J* = 3.8 Hz), 124.66 (d, *J* = 272.3 Hz), 118.84 (q, *J* = 3.4 Hz), 80.34, 58.95, 31.34, 28.44, 19.27, 17.88.

HRMS (ESI+): calculated: $C_{19}H_{27}NNaO_6^+$ $[M+Na]^+$: 388.1731, found: 388.1743.

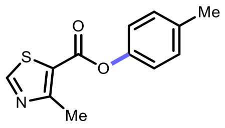

30

***p*-Tolyl 4-methylthiazole-5-carboxylate (30)**

The ester **30** was prepared using **method A** above in a 0.300 mmol scale, 4-methylthiazole-5-carboxylic acid does not dissolve well in acetonitrile after mixing with collidine, so 5%(v/v) anhydrous DMSO in MeCN was used for a better solubility. After sixteen hours, the reaction mixture was diluted with 10 mL ethyl acetate and washed with saturated sodium sulfate aqueous solution (2 × 10 mL). The organic layer was dried over anhydrous magnesium sulfate, filtered, followed by removal of the volatiles from the filtrate in vacuo.

Purification by flash chromatography on silica gel with gradient of 5% to 20% ethyl acetate in hexanes to give the product **30** (42.7 mg, 61%).

White solid, R_f = 0.31 (20% ethyl acetate in hexanes).

^{1}H NMR (700 MHz, $CDCl_3$): δ 8.85 (s, 1H), 7.21 (d, *J* = 8.0 Hz, 2H), 7.08 (d, *J* = 8.7 Hz, 2H), 2.84 (s, 3H), 2.37 (s, 3H).

^{13}C NMR (176 MHz, $CDCl_3$): δ 162.34, 160.82, 156.13, 148.08, 136.03, 130.13, 121.43, 121.36, 21.01, 17.63.

HRMS (ESI+): calculated: $C_{12}H_{12}NO_2S^+$ $[M+H]^+$: 234.0583, found: 234.0589.

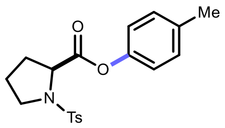

31

***p*-Tolyl tosyl-*L*-prolinate (31)**

The ester **31** was prepared using **method A** above in a 0.300 mmol scale. After sixteen hours, the reaction mixture was diluted with 10 mL ethyl acetate and washed with saturated sodium sulfate aqueous solution (2 × 10 mL). The organic layer was dried over anhydrous magnesium sulfate, filtered, followed by removal of the volatiles from the filtrate in vacuo.

Purification by flash chromatography on silica gel with gradient of 5% to 33% ethyl acetate in hexanes to give the product **31** (61.4 mg, 57%).

White solid, R_f = 0.17 (20% ethyl acetate in hexanes).

^{1}H NMR (500 MHz, $CDCl_3$): δ 7.82 – 7.79 (m, 2H), 7.31 (d, *J* = 8.1 Hz, 2H), 7.16 (d, *J* = 8.1 Hz, 2H), 7.00 – 6.95 (m, 2H), 4.47 (dd, *J* = 7.4, 5.6 Hz, 1H), 3.54 (ddd, *J* = 9.5, 7.3, 4.9 Hz, 1H), 3.37 (dt, *J* = 9.5, 7.2 Hz, 1H), 2.42 (s, 3H), 2.34 (s, 3H), 2.22 – 2.15 (m, 2H), 2.11 – 2.01 (m, 1H), 1.87 – 1.79 (m, 1H).

^{13}C NMR (176 MHz, $CDCl_3$): δ 171.05, 148.43, 143.82, 135.76, 135.34, 130.02, 129.83, 127.71, 121.10, 60.59, 48.62, 31.20, 24.89, 21.66, 20.99.

HRMS (ESI+): calculated: $C_{19}H_{21}NNaO_4S^+$ $[M+Na]^+$: 382.1083, found: 382.1078.

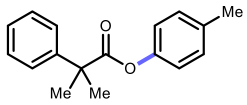

32

p-Tolyl 2-methyl-2-phenylpropanoate (32)

The ester **32** was prepared using **method A** above in a 0.300 mmol scale. After sixteen hours, the reaction mixture was diluted with 10 mL ethyl acetate and washed with saturated sodium sulfate aqueous solution (2 × 10 mL). The organic layer was dried over anhydrous magnesium sulfate, filtered, followed by removal of the volatiles from the filtrate in vacuo.

Purification by flash chromatography on silica gel with gradient of 5% to 15% ethyl acetate in hexanes to give the product **32** (45.0 mg, 52%).

Clear liquid, R_f = 0.63 (20% ethyl acetate in hexanes).

^{1}H NMR (700 MHz, $CDCl_3$): δ 7.49 – 7.46 (m, 2H), 7.43 – 7.37 (m, 2H), 7.32 – 7.28 (m, 1H), 7.15 – 7.12 (m, 2H), 6.88 – 6.85 (m, 2H), 2.33 (s, 3H), 1.74 (s, 6H).

^{13}C NMR (176 MHz, $CDCl_3$): δ 175.61, 148.91, 144.36, 135.38, 129.92, 128.68, 127.01, 125.77, 121.08, 46.94, 26.59, 20.98.

HRMS (APCI+): calculated: $C_{17}H_{19}NO_2^+$ $[M+H]^+$: 255.1380, found: 255.1391.

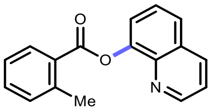

33

Quinolin-8-yl 2-methylbenzoate (33)

The ester **33** was prepared using **method A** above in a 0.300 mmol scale with 0.5 equiv. $Cu(MeCN)_4BF_4$. After sixteen hours, the reaction mixture was diluted with 10 mL ethyl acetate and washed with saturated sodium sulfate aqueous solution (2 × 10 mL). The organic layer was dried over anhydrous magnesium sulfate, filtered, followed by removal of the volatiles from the filtrate in vacuo.

Purification by flash chromatography on silica gel with gradient of 10% to 33% ethyl acetate in hexanes to give the product **33** (52.1 mg, 66%).

White solid, R_f = 0.65 (33% ethyl acetate in hexanes).

^{1}H NMR (700 MHz, $CDCl_3$): δ 8.90 (dd, *J* = 4.1, 1.7 Hz, 1H), 8.39 (dd, *J* = 7.8, 1.4 Hz, 1H), 8.20 (dd, *J* = 8.3, 1.7 Hz, 1H), 7.77 (dd, *J* = 7.9, 1.6 Hz, 1H), 7.61 – 7.55 (m, 2H), 7.50 (td, *J* = 7.6, 1.5 Hz, 1H), 7.43 (dd, *J* = 8.3, 4.1 Hz, 1H), 7.39 – 7.33 (m, 2H), 2.73 (s, 3H).

^{13}C NMR (176 MHz, $CDCl_3$): δ 166.37, 150.70, 147.98, 141.62, 141.57, 136.06, 132.77, 131.96, 131.80, 129.76, 128.90, 126.39, 126.07, 126.01, 121.86, 121.80, 21.99.

HRMS (ESI+): calculated: $C_{17}H_{12}NNaO_2^+$ $[M+Na]^+$: 286.0838, found: 286.0839.

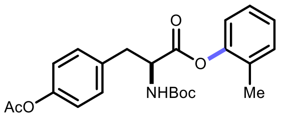

34

***o*-Tolyl *(S)*-3-(4-acetoxyphenyl)-2-((*tert*-butoxycarbonyl)amino)propanoate (34)**

The ester **34** was prepared using **method A** above in a 0.300 mmol scale. After the reaction was determined complete, the reaction mixture was diluted with 10 mL ethyl acetate and washed with saturated sodium sulfate aqueous solution (2 × 10 mL). The organic layer was dried over anhydrous magnesium sulfate, filtered, followed by removal of the volatiles from the filtrate in vacuo.

Purification by flash chromatography on silica gel with gradient of 5% to 33% ethyl acetate in hexanes to give the product **34** (39.7 mg, 32%).

White solid, R_f = 0.24 (20% ethyl acetate in hexanes).

^{1}H NMR (700 MHz, $CDCl_3$): δ 7.28 (d, *J* = 8.2 Hz, 2H), 7.22 – 7.17 (m, 2H), 7.14 (t, *J* = 7.4 Hz, 1H), 7.07 (d, *J* = 8.4 Hz, 2H), 6.88 (d, *J* = 8.0 Hz, 1H), 5.05 (d, *J* = 8.4 Hz, 1H), 4.83 (dt, *J* = 7.1 Hz, 1H), 3.29 (dd, *J* = 14.0, 6.0 Hz, 1H), 3.20 (dd, *J* = 14.1, 7.0 Hz, 1H), 2.30 (s, 3H), 2.12 (s, 3H), 1.44 (s, 9H).

^{13}C NMR (176 MHz, $CDCl_3$): δ 170.52, 169.56, 155.30, 150.01, 149.11, 133.54, 131.36, 130.59, 130.17, 127.10, 126.42, 121.96, 121.66, 80.41, 54.66, 37.75, 28.43, 21.30, 16.31.

HRMS (ESI+): calculated: $C_{23}H_{27}NNaO_6^+$ $[M+Na]^+$: 436.1731, found: 436.1723.

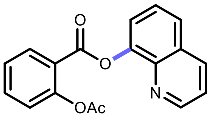

35

Quinolin-8-yl 2-acetoxybenzoate (35)

The ester **35** was prepared following **method A** above in a 0.300 mmol scale. After the reaction was determined complete, the reaction mixture was diluted with 10 mL ethyl acetate and washed with saturated sodium sulfate aqueous solution (2 × 10 mL). The organic layer was dried over anhydrous magnesium sulfate, filtered, followed by removal of the volatiles from the filtrate in vacuo.

Purification by flash chromatography on silica gel with gradient of 5% to 33% ethyl acetate in hexanes to give the product **35** (59.0 mg, 64%).

White solid, R_f = 0.11 (20% ethyl acetate in hexanes).

^{1}H NMR (700 MHz, $CDCl_3$): δ 8.90 (dd, *J* = 4.2, 1.7 Hz, 1H), 8.46 (dd, *J* = 7.8, 1.7 Hz, 1H), 8.19 (dd, *J* = 8.3, 1.7 Hz, 1H), 7.77 (dd, *J* = 8.0, 1.5 Hz, 1H), 7.66 (ddd, *J* = 8.2, 7.4, 1.7 Hz, 1H), 7.58 (t, *J* = 7.7 Hz, 1H), 7.55 (dd, *J* = 7.5, 1.5 Hz, 1H), 7.45 – 7.42 (m, 2H), 7.21 (dd, *J* = 8.2, 1.2 Hz, 1H), 2.27 (s, 3H).

^{13}C NMR (176 MHz, $CDCl_3$): δ 169.71, 163.06, 151.32, 150.64, 147.33, 141.25, 135.85, 134.41, 132.77, 129.57, 126.21, 126.20, 126.07, 123.99, 122.70, 121.77, 121.60, 21.04.

HRMS (ESI+): calculated: $C_{18}H_{13}NNaO_4^+$ $[M+Na]^+$: 330.0737, found: 330.0733.

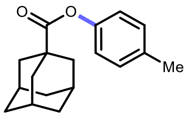

36

_p_-Tolyl (3r,5r,7r)-adamantane-1-carboxylate (36)

The ester **36** was prepared following **method A** above in a 0.300 mmol scale. After the reaction was determined complete, the reaction mixture was diluted with 10 mL ethyl acetate and washed with saturated sodium sulfate aqueous solution (2 × 10 mL). The organic layer was dried over anhydrous magnesium sulfate, filtered, followed by removal of the volatiles from the filtrate in vacuo.

Purification by flash chromatography on silica gel with gradient of 5% to 15% ethyl acetate in hexanes to give the product **36** (39.0 mg, 48%).

White solid, R_f = 0.68 (20% ethyl acetate in hexanes).

^{1}H NMR (700 MHz, $CDCl_3$): δ 7.17 – 7.13 (m, 2H), 6.93 – 6.90 (m, 2H), 2.34 (s, 3H), 2.08 (p, *J* = 3.1 Hz, 3H), 2.05 (d, *J* = 2.9 Hz, 6H), 1.80 – 1.73 (m, 6H).

^{13}C NMR (176 MHz, $CDCl_3$): δ 176.51, 148.95, 135.19, 129.95, 121.35, 41.10, 38.90, 36.61, 28.06, 21.00.

HRMS (APCI+): calculated: $C_{18}H_{23}O_2^+$ [M+H]$^+$: 271.1693, found: 271.1696.

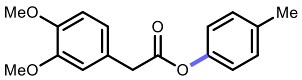

37

p-Tolyl 2-(3,4-dimethoxyphenyl)acetate (37)

The ester **37** was prepared following **method A** above in a 0.300 mmol scale. After the reaction was determined complete, the reaction mixture was diluted with 10 mL ethyl acetate and washed with saturated sodium sulfate aqueous solution (2 × 10 mL). The organic layer was dried over anhydrous magnesium sulfate, filtered, followed by removal of the volatiles from the filtrate in vacuo.

Purification by flash chromatography on silica gel with gradient of 5% to 15% ethyl acetate in hexanes to give the product **37** (67.9 mg, 79%).

White solid, R_f = 0.26 (20% ethyl acetate in hexanes).

^{1}H NMR (700 MHz, $CDCl_3$): δ 7.16 – 7.14 (m, 2H), 6.95 – 6.91 (m, 4H), 6.87 – 6.84 (m, 1H), 3.90 (s, 2H)3.89 (s, 3H), 3.79 (s, 2H), 2.33 (s, 3H).

^{13}C NMR (176 MHz, $CDCl_3$): δ 170.57, 149.09, 148.59, 148.39, 135.62, 130.01, 126.06, 121.60, 121.20, 112.44, 111.34, 56.01, 55.99, 41.06, 20.98.

HRMS (APCI+): calculated: $C_{17}H_{19}O_4^+$ [M+H]$^+$: 287.1278, found: 287.1286.

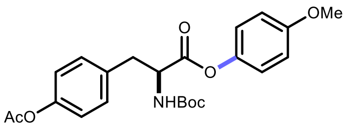

38

4-Methoxyphenyl *(S)*-3-(4-acetoxyphenyl)-2-((*tert*-butoxycarbonyl)amino)propanoate (38)

The ester **38** was prepared following **method A** above in a 0.300 mmol scale. After sixteen hours, the reaction mixture was diluted with 10 mL ethyl acetate and washed with saturated sodium sulfate aqueous solution (2 × 10 mL). The organic layer was dried over anhydrous magnesium sulfate, filtered, followed by removal of the volatiles from the filtrate in vacuo.

Purification by flash chromatography on silica gel with gradient of 5% to 15% ethyl acetate in hexanes to give the product **38** (88.0 mg, 71%).

White solid, R_f = 0.14 (20% ethyl acetate in hexanes).

^{1}H NMR (700 MHz, $CDCl_3$): δ 7.26 – 7.23 (m, 2H), 7.07 – 7.05 (m, 2H), 6.91 – 6.85 (m, 4H), 5.10 (d, *J* = 8.3 Hz, 1H), 4.79 (q, *J* = 7.0 Hz, 1H), 3.78 (s, 3H), 3.22 (d, *J* = 6.2 Hz, 2H), 2.30 (s, 3H), 1.45 (s, 9H).

^{13}C NMR (176 MHz, $CDCl_3$): δ 170.89, 169.50, 157.56, 155.22, 149.99, 143.86, 133.54, 130.58, 122.15, 121.90, 114.61, 80.34, 55.70, 54.67, 37.91, 28.43, 21.25.

HRMS (ESI+): calculated: $C_{23}H_{27}NNaO_7^+$ $[M+Na]^+$: 452.1680, found: 452.1684.

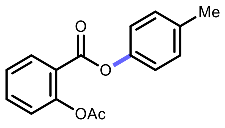

42

p-Tolyl 2-acetoxybenzoate (42)

The ester **42** was prepared following **method A** above in a 0.300 mmol scale. After sixteen hours, the reaction mixture was diluted with 10 mL ethyl acetate and washed with saturated sodium sulfate aqueous solution (2 × 10 mL). The organic layer was dried over anhydrous magnesium sulfate, filtered, followed by removal of the volatiles from the filtrate in vacuo. When using aspirin pills (Rite Aid Pharmacy®, lot # P118181, 81 mg aspirin per pill) for the esterification reaction, three pills were grinded to fine powder and the average mass was obtained. The fine powder was used (based on the net weight) directly following **method A** without filtration.

Purification by flash chromatography on silica gel with gradient of 5% to 20% ethyl acetate in hexanes to give the product **42** (74.6 mg, 92%, 80% using aspirin pills).

White solid, R_f = 0.4 (20% ethyl acetate in hexanes).

^{1}H NMR (700 MHz, $CDCl_3$): δ 8.24 (dd, J = 7.8, 1.7 Hz, 1H), 7.64 (ddd, J = 8.2, 7.4, 1.7 Hz, 1H), 7.39 (td, J = 7.7, 1.2 Hz, 1H), 7.23 (dd, J = 8.7, 0.7 Hz, 2H), 7.18 (dd, J = 8.1, 1.2 Hz, 1H), 7.09 – 7.06 (m, 2H), 2.38 (s, 3H), 2.32 (s, 3H).

^{13}C NMR (176 MHz, $CDCl_3$): δ 169.81, 163.27, 151.25, 148.41, 135.85, 134.58, 132.30, 130.19, 126.25, 124.08, 122.78, 121.44, 21.11, 20.99.

HRMS (ESI+): calculated: $C_{16}H_{14}NaO_4^+$ $[M+Na]^+$: 293.0784, found: 293.0857.

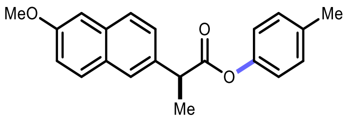

43

***p*-Tolyl *(S)*-2-(6-methoxynaphthalen-2-yl)propanoate (43)**

The ester **43** was prepared following **method A** above in a 0.300 mmol scale. After sixteen hours, the reaction mixture was diluted with 10 mL ethyl acetate and washed with saturated sodium sulfate aqueous solution (2 × 10 mL). The organic layer was dried over anhydrous magnesium sulfate, filtered, followed by removal of the volatiles from the filtrate in vacuo. When using naproxen sodium pills (Bayer Aleve®, lot # NAA8NKN, 220 mg naproxen sodium per pill) for the esterification reaction, three pills were grinded to fine powder and the average mass was obtained. The fine powder was used (based on the net weight) directly following **method A** without filtration. 5% (v/v) DMSO in MeCN was used as the solvent to accommodate the solubility.

Purification by flash chromatography on silica gel with gradient of 5% to 20% ethyl acetate in hexanes to give the product **43** (84.6 mg, 88%, 61% using naproxen sodium pills).

Orange solid (both from pills and reagent), R_f = 0.54 (20% ethyl acetate in hexanes).

^{1}H NMR (700 MHz, $CDCl_3$): δ 7.80 (d, *J* = 1.8 Hz, 1H), 7.77 (dd, *J* = 11.8, 8.7 Hz, 2H), 7.54 (dd, *J* = 8.5, 1.9 Hz, 1H), 7.19 (dd, *J* = 8.8, 2.5 Hz, 1H), 7.16 (d, *J* = 2.5 Hz, 1H), 7.13 (dd, *J* = 8.7, 0.8 Hz, 2H), 6.92 – 6.88 (m, 2H), 4.11 (q, *J* = 7.1 Hz, 1H), 3.94 (s, 3H), 2.33 (s, 3H), 1.72 (d, *J* = 7.2 Hz, 3H).

^{13}C NMR (176 MHz, $CDCl_3$): δ 173.45, 157.85, 148.72, 135.45, 135.38, 133.92, 129.92, 129.44, 129.11, 127.45, 126.27, 126.24, 121.16, 119.19, 105.73, 55.42, 45.68, 20.94, 18.69.

HRMS (ESI+): calculated: $C_{16}H_{14}NaO_4^+$ $[M+Na]^+$: 343.1305, found: 343.1306.

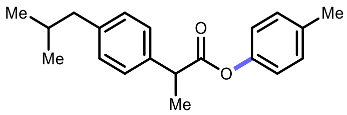

44

p-Tolyl 2-(4-isobutylphenyl)propanoate (44)

The ester **44** was prepared following **method A** above in a 0.300 mmol scale. After sixteen hours, the reaction mixture was diluted with 10 mL ethyl acetate and washed with saturated sodium sulfate aqueous solution (2 × 10 mL). The organic layer was dried over anhydrous magnesium sulfate, filtered, followed by removal of the volatiles from the filtrate in vacuo. When using ibuprofen pills (Advil®, lot # DJ6094, 200 mg ibuprofen per pill) for the esterification reaction, three pills were grinded to fine powder and the average mass was obtained. The fine powder was used (based on the net weight) directly following **method A** without filtration.

Purification by flash chromatography on silica gel with gradient of 10% to 30% dichloromethane in hexanes to give the product **44** (56.9 mg, 64%, 61% using ibuprofen pills).

White solid, R_f = 0.80 (20% ethyl acetate in hexanes).

^{1}H NMR (700 MHz, $CDCl_3$): δ 7.33 – 7.29 (m, 2H), 7.17 – 7.11 (m, 4H), 6.91 – 6.86 (m, 2H), 3.94 (q, *J* = 7.2 Hz, 1H), 2.49 (d, *J* = 7.2 Hz, 2H), 2.33 (s, 3H), 1.88 (tq, *J* = 13.5, 6.8 Hz, 1H), 1.61 (d, *J* = 7.2 Hz, 3H), 0.93 (d, *J* = 6.6 Hz, 6H).

^{13}C NMR (176 MHz, $CDCl_3$): δ 173.52, 148.77, 140.87, 137.49, 135.41, 129.93, 129.60, 127.35, 121.19, 45.39, 45.20, 30.33, 22.54, 22.54, 20.98, 18.71.

HRMS (ESI+): calculated: $C_{20}H_{24}NaO_2^+$ $[M+Na]^+$: 319.1669, found: 319.1670.

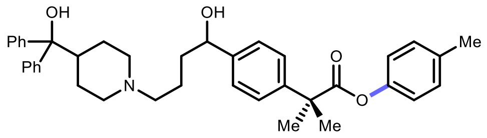

45

_p_-Tolyl 2-(4-(1-hydroxy-4-(4-(hydroxydiphenylmethyl)piperidin-1-yl)butyl)phenyl)-2-methylpropanoate (45)

The ester **45** was prepared following **method A** above in a 0.300 mmol scale. After sixteen hours, the reaction mixture was diluted with 10 mL ethyl acetate and washed with saturated sodium sulfate aqueous solution (2 × 10 mL). The organic layer was dried over anhydrous magnesium sulfate, filtered, followed by removal of the volatiles from the filtrate in vacuo. When using fexofenadine pills (Allegra Allergy®, lot # 20F204,180 mg fexofenadine HCl per pill) for the esterification reaction, three pills were grinded to fine powder and the average mass was obtained. The fine powder was used (based on the net weight) directly following **method A** without filtration. 5% (v/v) DMSO in MeCN was used as the solvent to accommodate the solubility.

Purification by flash chromatography on silica gel with gradient of dichloromethane to 5% methanol in dichloromethane to give the product **45** (47.9 mg, 27%, 15% using fexofenadine HCl tablets).

White solid, R_f = 0.41 (10% methanol in dichloromethane).

^{1}H NMR (700 MHz, $CDCl_3$): δ 7.47 (ddd, *J* = 8.3, 5.4, 1.3 Hz, 4H), 7.38 – 7.35 (m, 2H), 7.32 (d, *J* = 8.3 Hz, 2H), 7.28 – 7.24 (m, 4H), 7.18 – 7.14 (m, 2H), 7.10 (d, *J* = 8.2 Hz, 2H), 6.84 – 6.80 (m, 2H), 4.67 (dd, *J* = 8.2, 2.8 Hz, 1H), 3.34 (d, *J* = 11.8 Hz, 1H), 3.26 (d, *J* = 11.8 Hz, 1H), 2.73 (h, *J* = 6.8 Hz, 2H), 2.54 (tt, *J* = 12.0, 3.4 Hz, 1H), 2.39 (dt, *J* = 29.6, 12.3 Hz, 2H), 2.30 (s, 3H), 1.95 – 1.88 (m, 2H), 1.87 – 1.75 (m, 4H), 1.67 (d, *J* = 1.3 Hz, 6H), 1.57 – 1.52 (m, 2H).

^{13}C NMR (176 MHz, $CDCl_3$): δ 175.68, 148.87, 145.79, 145.73, 143.65, 143.09, 135.39, 129.92, 128.41, 126.73, 126.72, 126.04, 125.77, 125.72, 125.70, 125.67, 121.08, 79.13, 72.98, 57.63, 53.71, 53.10, 46.71, 43.13, 37.67, 26.64, 26.61, 24.54, 24.48, 22.33, 20.97.

HRMS (ESI+): calculated: $C_{39}H_{46}NO_4^+$ [M+H]$^+$: 592.3421, found: 592.3412.

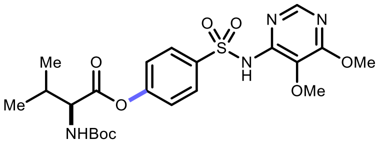

11

4-(*N*-(5,6-dimethoxypyrimidin-4-yl)sulfamoyl)phenyl (*tert*-butoxycarbonyl)-*L*-valinate (11)

The ester **11** was prepared following **method A** above in a 0.300 mmol scale, with $Cu(MeCN)_4BF_4$ (1.0 equiv. 0.300 mmol). After sixteen hours, the reaction mixture was diluted with 10 mL ethyl acetate and washed with saturated sodium sulfate aqueous solution (2 × 10 mL). The organic layer was dried over anhydrous magnesium sulfate, filtered, followed by removal of the volatiles from the filtrate in vacuo.

Purification by flash chromatography on silica gel with gradient of 5% to 33% acetone in hexanes to give the product **11** (115 mg, 75%).

White solid, R_f = 0.47 (ethyl acetate / dichloromethane 1:6).

^{1}H NMR (700 MHz, $CDCl_3$): δ 8.20 (d, *J* = 8.4 Hz, 2H), 8.16 (s, 1H), 7.89 (s, br, 1H), 7.27 – 7.24 (m, 2H), 5.03 (d, *J* = 8.9 Hz, 1H), 4.43 (dd, *J* = 9.0, 5.0 Hz, 1H), 3.98 (s, 3H), 3.86 (s, 3H), 2.30 (dq, *J* = 6.5 Hz, 1H), 1.46 (s, 9H), 1.08 (d, *J* = 6.8 Hz, 3H), 1.02 (d, *J* = 6.9 Hz, 3H).

^{13}C NMR (176 MHz, $CDCl_3$): δ 170.64, 161.00, 155.81, 154.29, 151.07, 149.62, 137.11, 130.46, 126.67, 121.94, 80.41, 60.71, 59.01, 54.32, 31.25, 28.43, 19.26, 17.90.

HRMS (ESI+): calculated: $C_{22}H_{31}N_4O_8S^+$ [M+H]$^+$: 511.1857, found: 511.1849.

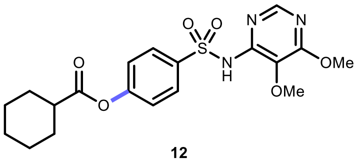

12

4-(*N*-(5,6-dimethoxypyrimidin-4-yl)sulfamoyl)phenyl cyclohexanecarboxylate (12)

The ester **12** was prepared following **method A** above in a 0.300 mmol scale, with $Cu(MeCN)_4BF_4$ (1.0 equiv. 0.300 mmol). After sixteen hours, the reaction mixture was diluted with 10 mL ethyl acetate and washed with saturated sodium sulfate aqueous solution (2 × 10 mL). The organic layer was dried over anhydrous magnesium sulfate, filtered, followed by removal of the volatiles from the filtrate in vacuo.

Purification by flash chromatography on silica gel with gradient of 20% to 33% acetone in hexanes to give the product **12** (84.7 mg, 67%).

White solid, R_f = 0.43 (33% acetone in hexanes).

^1H NMR (700 MHz, $CDCl_3$): δ 8.19 – 8.17 (m, 2H), 8.17 (s, 1H), 7.86 (s, br, 1H), 7.23 – 7.21 (m, 2H), 3.98 (s, 3H), 3.85 (s, 3H), 2.56 (tt, *J* = 11.2, 3.7 Hz, 1H), 2.04 (dt, *J* = 13.3, 3.7 Hz, 2H), 1.81 (dp, *J* = 11.4, 3.9 Hz, 2H), 1.69 (dp, *J* = 12.2, 4.1, 3.5 Hz, 1H), 1.57 (qd, *J* = 11.7, 3.6 Hz, 2H), 1.39 – 1.32 (m, 2H), 1.29 (tt, *J* = 12.1, 3.0 Hz, 1H).

^{13}C NMR (176 MHz, $CDCl_3$): δ 173.84, 160.98, 154.87, 151.08, 149.67, 136.52, 130.32, 126.63, 122.07, 60.71, 54.31, 43.30, 28.97, 25.75, 25.39.

HRMS (ESI+): calculated: $C_{19}H_{24}N_3O_6S^+$ [M+H]$^+$: 422.1380, found: 422.1375.

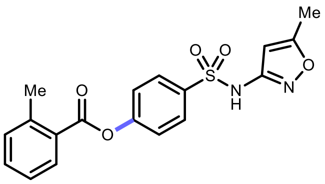

13

4-(*N*-(5-methylisoxazol-3-yl)sulfamoyl)phenyl 2-methylbenzoate (13)

The ester **13** was prepared following **method A** above in a 0.300 mmol scale, with $Cu(MeCN)_4BF_4$ (1.0 equiv. 0.300 mmol). After sixteen hours, the reaction mixture was diluted with 10 mL ethyl acetate and washed with saturated sodium sulfate aqueous solution (2 × 10 mL). The organic layer was dried over anhydrous magnesium sulfate, filtered, followed by removal of the volatiles from the filtrate in vacuo.

Purification by flash chromatography on silica gel with gradient of 20% to 33% acetone in hexanes to give the product **13** (30.1 mg, 27%).

White solid, R_f = 0.29 (33% acetone in hexanes).

^{1}H NMR (700 MHz, $CDCl_3$): δ 8.15 – 8.10 (m, 1H), 7.96 – 7.91 (m, 2H), 7.50 (td, *J* = 7.5, 1.5 Hz, 1H), 7.39 – 7.35 (m, 2H), 7.32 (td, *J* = 7.5, 0.7 Hz, 2H), 6.25 (s, 1H), 2.65 (s, 3H), 2.38 (s, 3H).

^{13}C NMR (176 MHz, $CDCl_3$): δ 171.36, 164.81, 157.28, 155.02, 141.99, 136.19, 133.49, 132.31, 131.42, 129.03, 127.57, 126.22, 123.05, 95.60, 22.17, 12.93.

HRMS (ESI+): calculated: $C_{18}H_{17}N_2O_5S^+$ $[M+H]^+$: 373.0853, found: 373.0857.

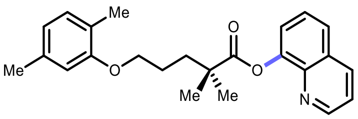

39

Quinolin-8-yl 5-(2,5-dimethylphenoxy)-2,2-dimethylpentanoate (39)

The ester **39** was prepared following **method A** above in a 0.300 mmol scale, with Cu(MeCN)$_4$BF$_4$ (0.50 equiv, 0.150 mmol), diazonium **7** (0.600 mmol, 2.0 equiv). After sixteen hours, the reaction mixture was diluted with 10 mL ethyl acetate and washed with saturated sodium sulfate aqueous solution (2 × 10 mL). The organic layer was dried over anhydrous magnesium sulfate, filtered, followed by removal of the volatiles from the filtrate in vacuo.

Purification by flash chromatography on silica gel with gradient of 5% to 20% ethyl acetate in hexanes to give the product **39** (59.0mg, 52%).

White solid, R_f = 0.51 (20% ethyl acetate in hexanes).

^{1}H NMR (700 MHz, CDCl$_3$): δ 8.83 (dd, *J* = 4.2, 0.9 Hz, 1H), 8.15 (d, *J* = 8.3 Hz, 1H), 7.70 (d, *J* = 8.2 Hz, 1H), 7.51 (t, *J* = 7.4 Hz, 1H), 7.39 (dd, *J* = 8.3, 3.3 Hz, 1H), 7.37 (dt, *J* = 7.4, 1.1 Hz, 1H), 7.01 (d, *J* = 7.7 Hz, 1H), 6.67 (d, *J* = 6.1 Hz, 2H), 4.06 (t, *J* = 6.4 Hz, 2H), 2.30 (s, 3H), 2.20 (s, 3H), 2.14 – 2.09 (m, 2H), 2.04 – 2.00 (m, 2H), 1.51 (d, *J* = 0.9 Hz, 6H).

^{13}C NMR (176 MHz, CDCl$_3$): δ 176.82, 157.20, 150.44, 148.03, 141.57, 136.60, 135.88, 130.42, 129.60, 126.25, 125.83, 123.81, 121.77, 121.46, 120.74, 112.15, 68.37, 42.84, 37.52, 25.60, 25.42, 21.57, 15.99.

HRMS (ESI+): calculated: $C_{24}H_{28}NO_3^+$ [M+H]$^+$: 378.2064, found: 378.2051.

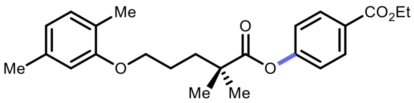

40

Ethyl 4-((5-(2,5-dimethylphenoxy)-2,2-dimethylpentanoyl)oxy)benzoate (40)

The ester **40** was prepared following **method A** above in a 0.300 mmol scale. After sixteen hours, the reaction mixture was diluted with 10 mL ethyl acetate and washed with saturated sodium sulfate aqueous solution (2 × 10 mL). The organic layer was dried over anhydrous magnesium sulfate, filtered, followed by removal of the volatiles from the filtrate in vacuo.

Purification by flash chromatography on silica gel with gradient of 5% to 20% ethyl acetate in hexanes to give the product **40** (94.4 mg, 79%).

White solid, R_f = 0.63 (20% ethyl acetate in hexanes).

^{1}H NMR (700 MHz, $CDCl_3$): δ 8.10 – 8.06 (m, 2H), 7.14 – 7.09 (m, 2H), 7.02 (d, *J* = 7.4 Hz, 1H), 6.68 (d, *J* = 8.4 Hz, 1H), 6.64 (s, 1H), 4.39 (q, *J* = 7.1 Hz, 2H), 4.00 (t, *J* = 5.6 Hz, 2H), 2.32 (s, 3H), 2.19 (s, 3H), 1.93 – 1.87 (m, 4H), 1.41 (t, *J* = 7.1 Hz, 3H), 1.40 (s, 6H).

^{13}C NMR (176 MHz, $CDCl_3$): δ 175.94, 165.96, 156.94, 154.75, 136.60, 131.18, 130.49, 128.05, 123.70, 121.64, 120.92, 112.05, 67.79, 61.17, 42.69, 37.23, 25.38, 25.23, 25.22, 21.53, 15.91, 14.45.

HRMS (ESI+): calculated: $C_{24}H_{30}NaO_5^+$ $[M+H]^+$: 421.1985, found: 421.1980.

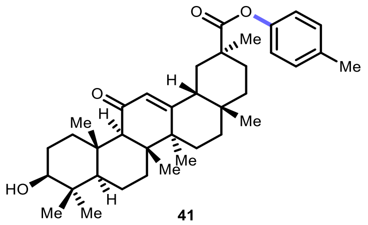

41

_p_-Tolyl (2_S_,4a_S_,6a_S_,6b_R_,8a_R_,10_S_,12a_S_,12b_R_,14b_R_)-10-hydroxy-2,4a,6a,6b,9,9,12a-heptamethyl-13-oxo-1,2,3,4,4a,5,6,6a,6b,7,8,8a,9,10,11,12,12a,12b,13,14b-icosahydropicene-2-carboxylate (41)

The ester **41** was prepared following **method A** above in a 0.300 mmol scale, 10% anhydrous DMSO in MeCN is used as the solvent to achieve better solubility of the acid. After sixteen hours, the reaction mixture was diluted with 10 mL ethyl acetate and washed with saturated sodium sulfate aqueous solution (2 × 10 mL). The organic layer was dried over anhydrous magnesium sulfate, filtered, followed by removal of the volatiles from the filtrate in vacuo.

Purification by flash chromatography on silica gel with gradient of 10% to 66% diethyl ether in hexanes to give the product **41** (144.7 mg, 86%).

White solid, R_f = 0.54 (33% ethyl acetate in hexanes).

^{1}H NMR (700 MHz, $CDCl_3$): δ 7.17 (d, *J* = 7.7 Hz, 2H), 6.94 – 6.89 (m, 2H), 5.68 (s, 1H), 3.22 (dd, *J* = 11.4, 4.9 Hz, 1H), 2.78 (dt, *J* = 13.5, 3.6 Hz, 1H), 2.34 (s, 1H), 2.34 (s, 3H), 2.26 (ddd, *J* = 13.5, 4.4, 1.7 Hz, 1H), 2.13 – 2.09 (m, 1H), 2.09 – 2.04 (m, 2H), 1.86 (td, *J* = 13.7, 4.8 Hz, 1H), 1.73 (t, *J* = 13.6 Hz, 1H), 1.68 – 1.58 (m, 4H), 1.50 – 1.40 (m, 5H), 1.39 (s, 3H), 1.33 (s, 3H), 1.21 (ddd, *J* = 13.9, 4.6, 2.4 Hz, 1H), 1.14 (s, 3H), 1.13 (s, 3H), 1.06 (ddt, *J* = 13.6, 4.6, 2.1 Hz, 1H), 0.99 (s, 3H), 1.00 – 0.94 (m, 1H), 0.85 (s, 3H), 0.80 (s, 3H), 0.70 (d, *J* = 11.8 Hz, 1H).

^{13}C NMR (176 MHz, $CDCl_3$): δ 200.26, 175.36, 169.00, 148.65, 135.53, 130.07, 128.80, 121.22, 78.89, 61.96, 55.09, 48.59, 45.53, 44.35, 43.36, 41.29, 39.27, 39.24, 37.88, 37.23, 32.92, 32.07, 31.31, 28.73, 28.26, 28.24, 27.45, 26.63, 26.59, 23.56, 21.00, 18.83, 17.63, 16.49, 15.72.

HRMS (ESI+): calculated: $C_{37}H_{53}O_4^+$ $[M+H]^+$: 561.3938, found: 561.3934.

Supplementary Note 1

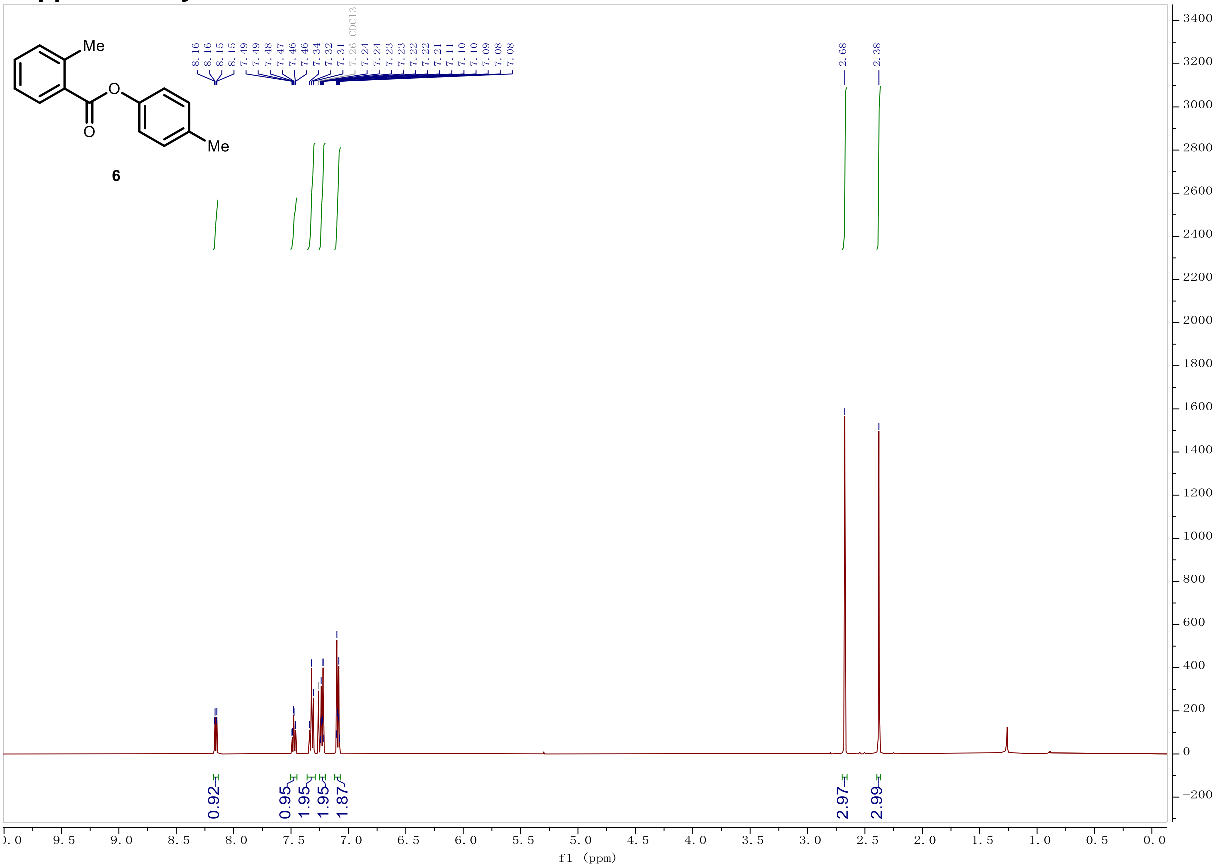

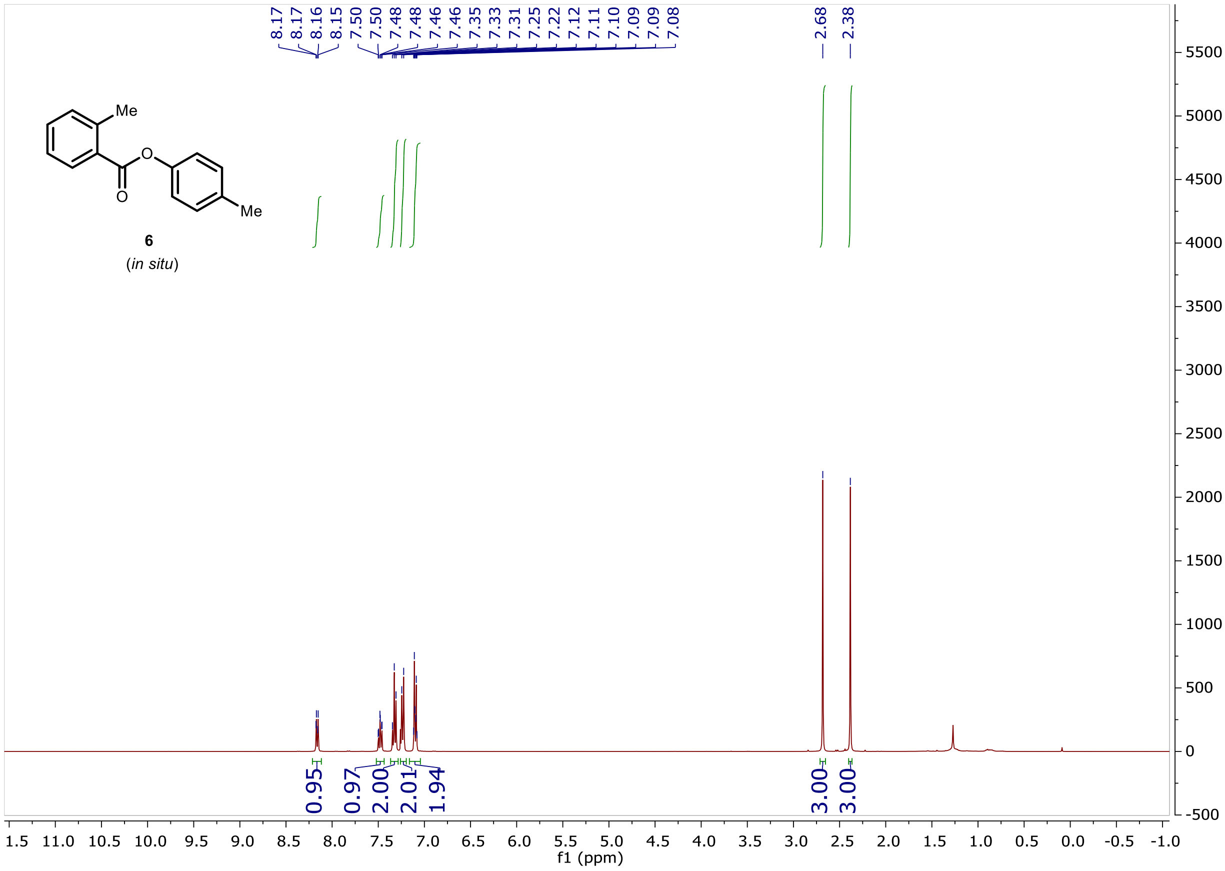
8.17
8.17
8.16
8.15
7.50
7.48
7.48
7.46
7.46
7.35
7.33
7.31
7.25
7.22
7.12
7.11
7.10
7.09
7.09
7.08
2.68
2.38
Me
O
O
Me
6
(in situ)
0.95
0.97
2.00
2.01
1.94
3.00
3.00
f1 (ppm)

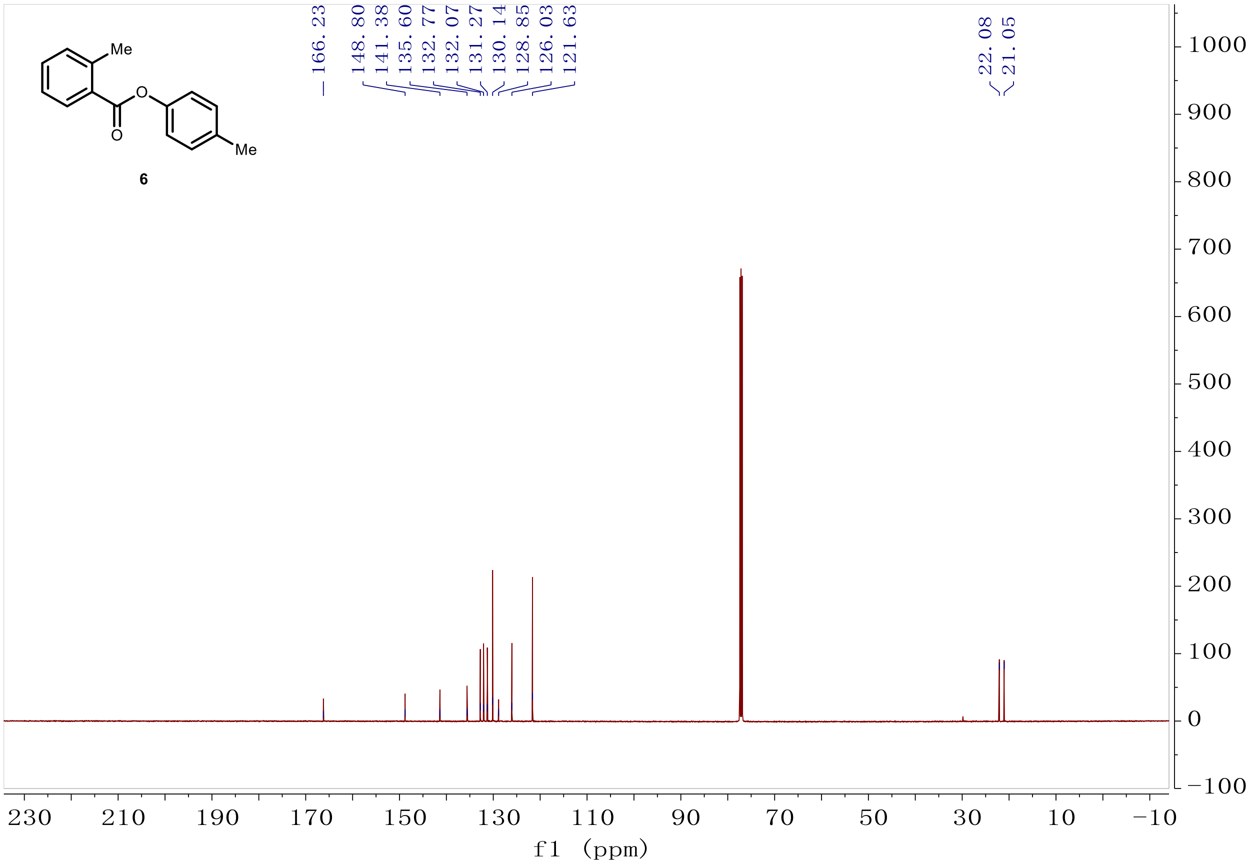

Me
O
O
Me
6
166.23
148.80
141.38
135.60
132.77
132.07
131.27
130.14
128.85
126.03
121.63
22.08
21.05
1000
900
800
700
600
500
400
300
200
100
0
−100
230
210
190
170
150
130
110
90
70
50
30
10
−10
f1 (ppm)

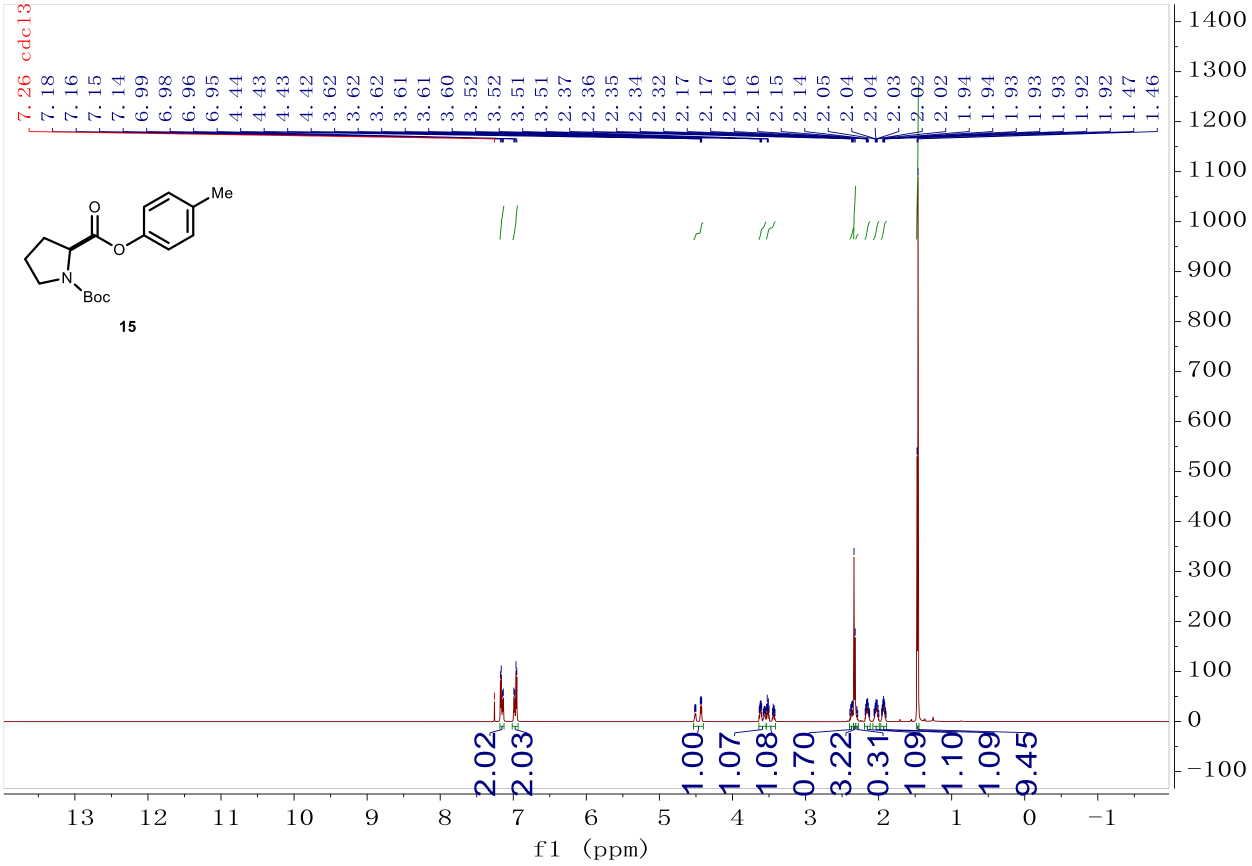
cdcl3
Me
O
O
N
Boc
15
f1 (ppm)

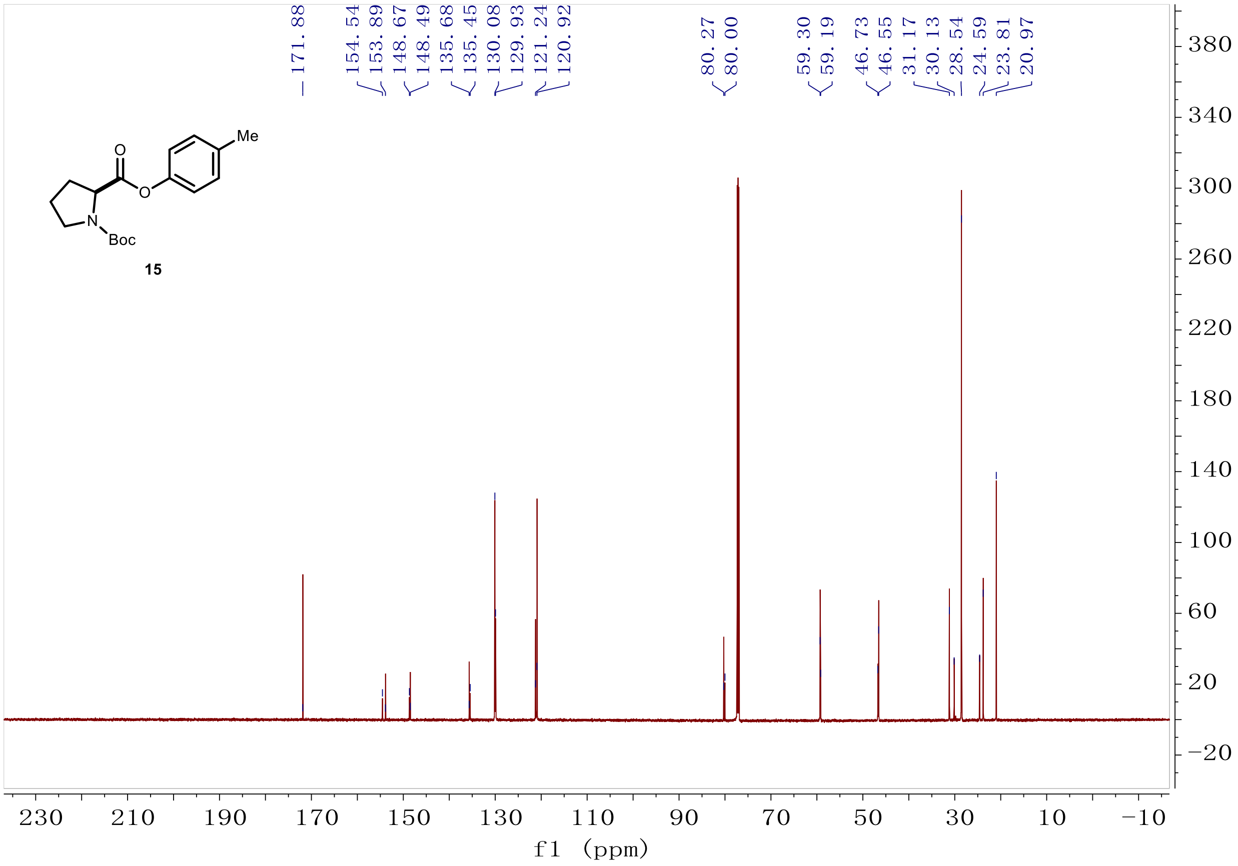
171.88
154.54
153.89
148.67
148.49
135.68
135.45
130.08
129.93
121.24
120.92
80.27
80.00
59.30
59.19
46.73
46.55
31.17
30.13
28.54
24.59
23.81
20.97
O
O
Me
N
Boc
15
380
340
300
260
220
180
140
100
60
20
-20
230
210
190
170
150
130
110
90
70
50
30
10
-10
f1 (ppm)

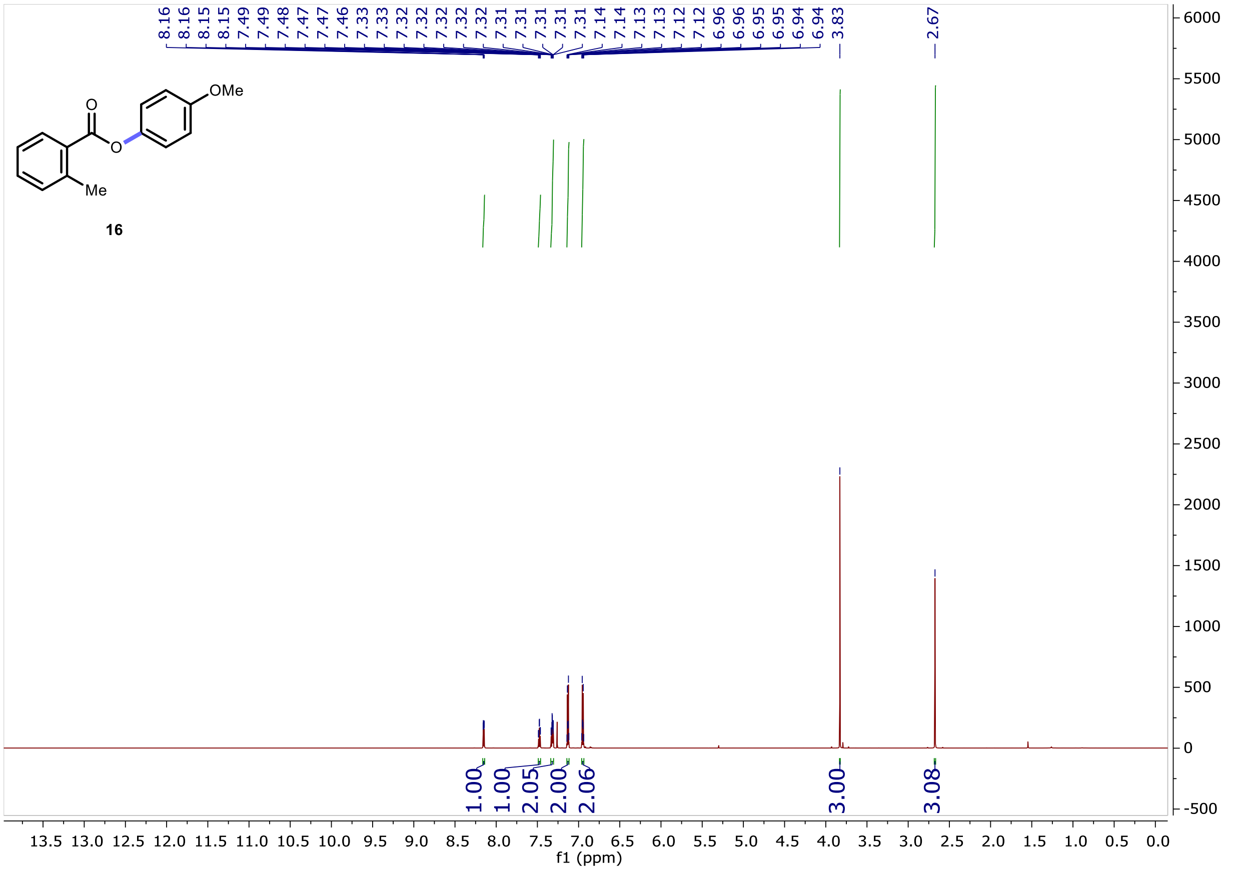
OMe
O
O
Me
16
8.16
8.16
8.15
8.15
7.49
7.49
7.48
7.47
7.47
7.46
7.33
7.33
7.32
7.32
7.32
7.32
7.32
7.31
7.31
7.31
7.31
7.31
7.14
7.14
7.13
7.13
7.12
7.12
6.96
6.96
6.95
6.95
6.94
6.94
3.83
2.67
1.00
1.00
2.05
2.00
2.06
3.00
3.08
f1 (ppm)

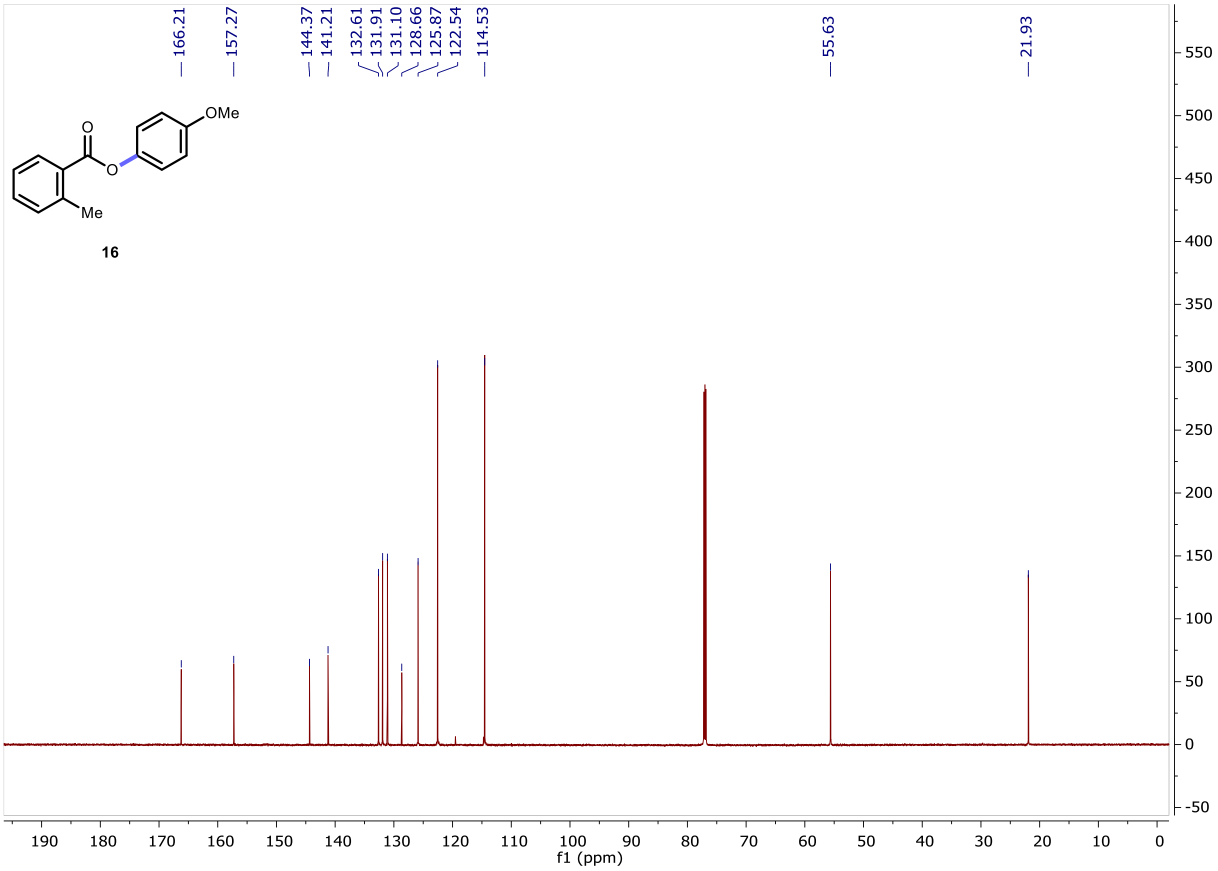
166.21
157.27
144.37
141.21
132.61
131.91
131.10
128.66
125.87
122.54
114.53
55.63
21.93
OMe
O
O
Me
16
f1 (ppm)

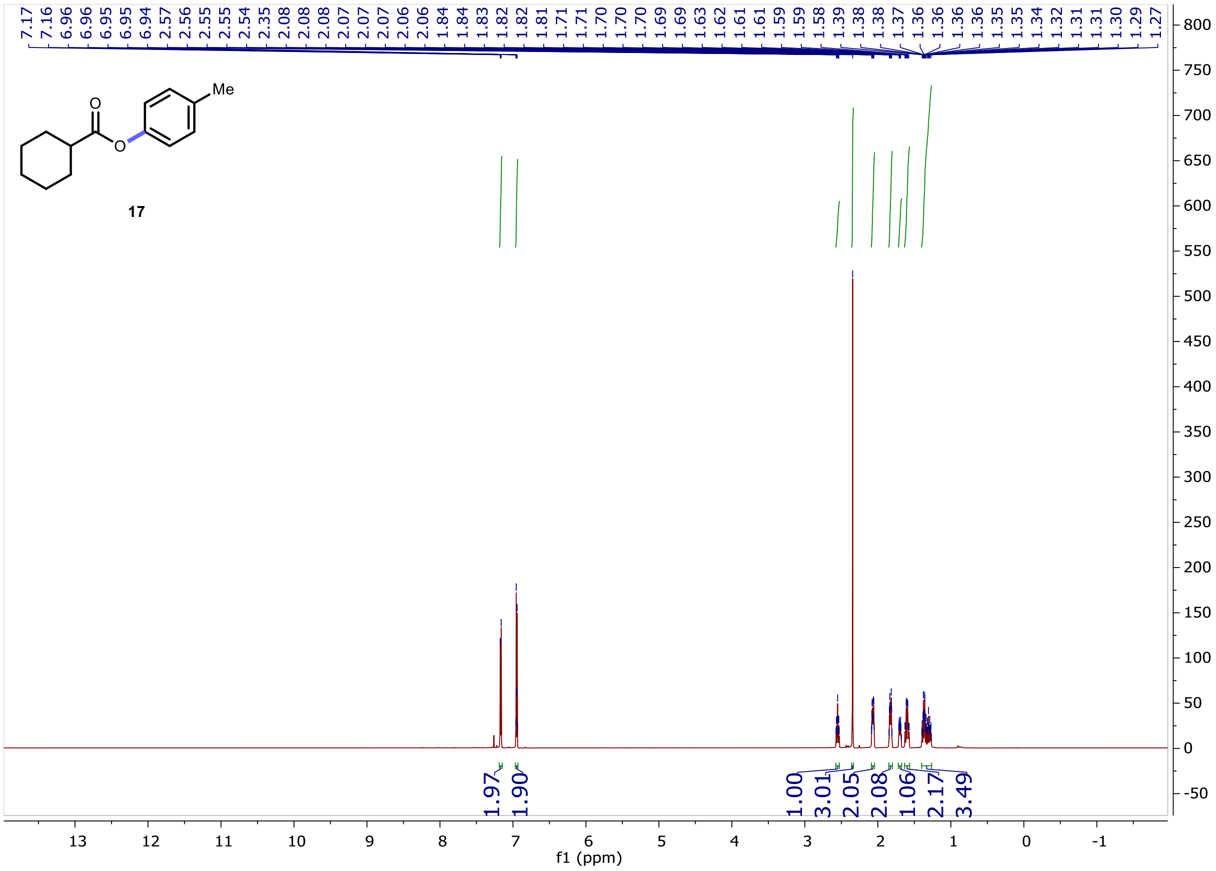
7.17 7.16 6.96 6.96 6.95 6.95 6.94 2.57 2.56 2.55 2.55 2.54 2.35 2.08 2.08 2.08 2.07 2.07 2.07 2.06 2.06 1.84 1.84 1.83 1.82 1.82 1.81 1.71 1.71 1.70 1.70 1.70 1.69 1.69 1.63 1.62 1.61 1.61 1.59 1.59 1.58 1.39 1.38 1.38 1.37 1.36 1.36 1.36 1.36 1.35 1.35 1.34 1.32 1.31 1.30 1.29 1.27
O
O
Me
17
1.97
1.90
1.00
3.01
2.05
2.08
1.06
2.17
3.49
f1 (ppm)

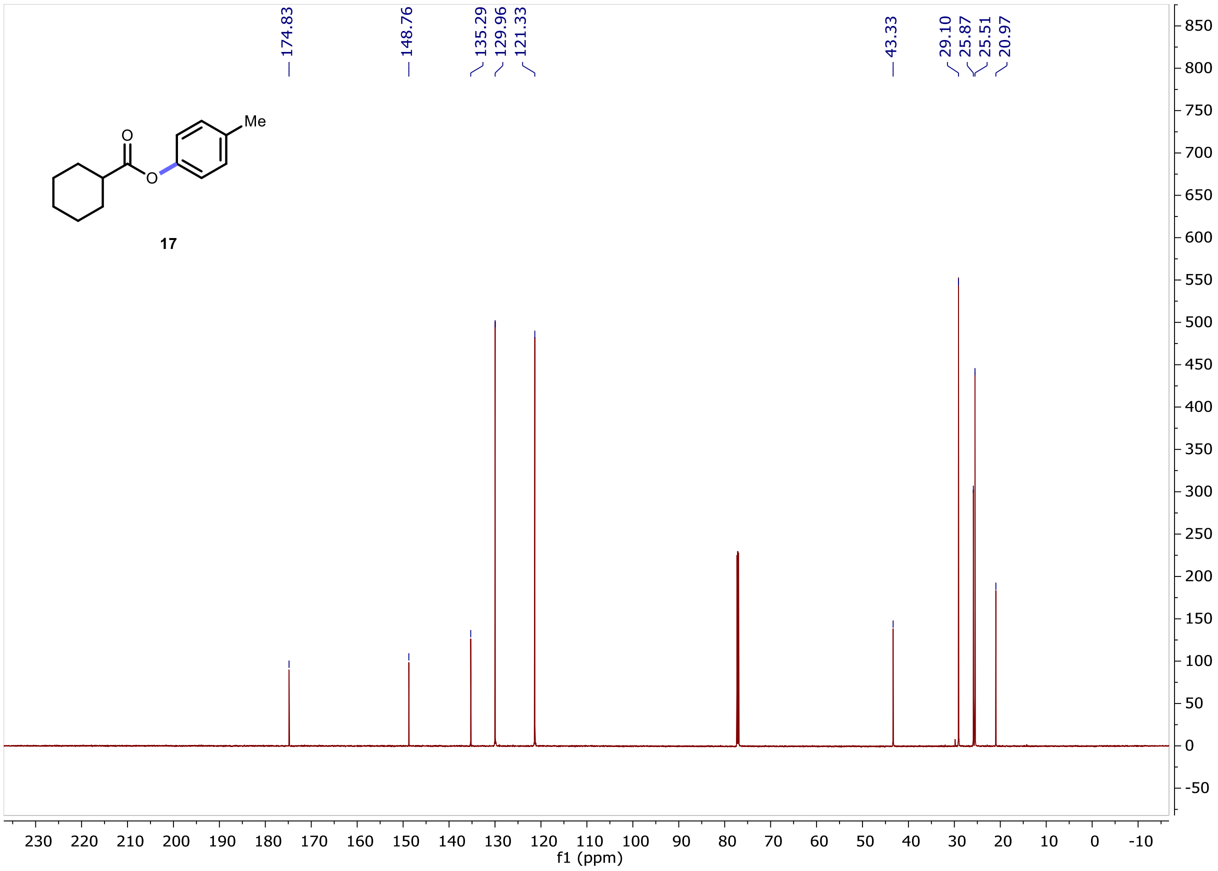

174.83
148.76
135.29
129.96
121.33
43.33
29.10
25.87
25.51
20.97
O
O
Me
17
f1 (ppm)

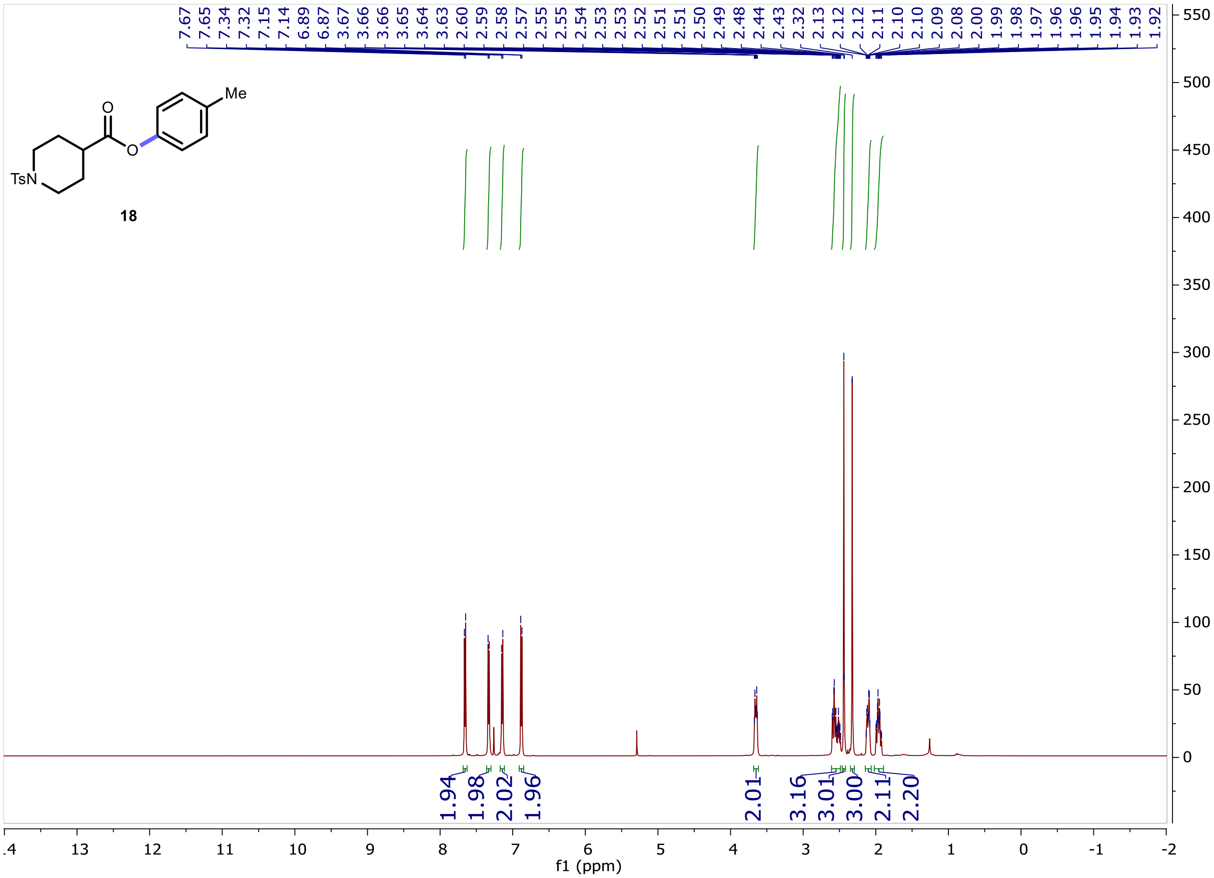
7.67
7.65
7.34
7.32
7.15
7.14
6.89
6.87
3.67
3.66
3.65
3.64
3.63
2.60
2.59
2.58
2.57
2.55
2.54
2.53
2.53
2.52
2.51
2.51
2.50
2.49
2.48
2.44
2.43
2.32
2.13
2.12
2.12
2.11
2.10
2.10
2.09
2.08
2.00
1.99
1.98
1.97
1.96
1.96
1.95
1.94
1.93
1.92
Me
O
O
TsN
18
1.94
1.98
2.02
1.96
2.01
3.16
3.01
3.00
2.11
2.20
f1 (ppm)

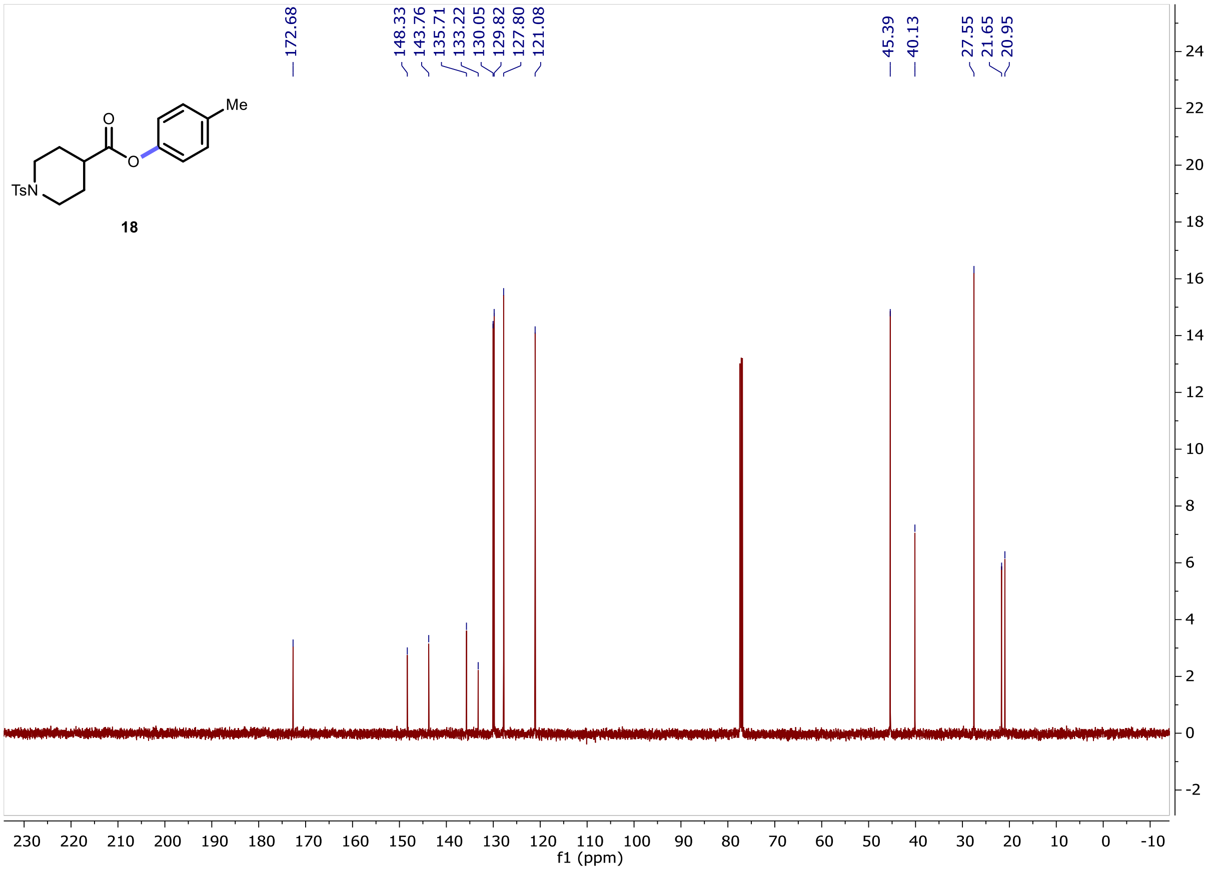

172.68
148.33
143.76
135.71
133.22
130.05
129.82
127.80
121.08
45.39
40.13
27.55
21.65
20.95
Me
O
O
TsN
18
24
22
20
18
16
14
12
10
8
6
4
2
0
-2
230
220
210
200
190
180
170
160
150
140
130
120
110
100
90
80
70
60
50
40
30
20
10
0
-10
f1 (ppm)

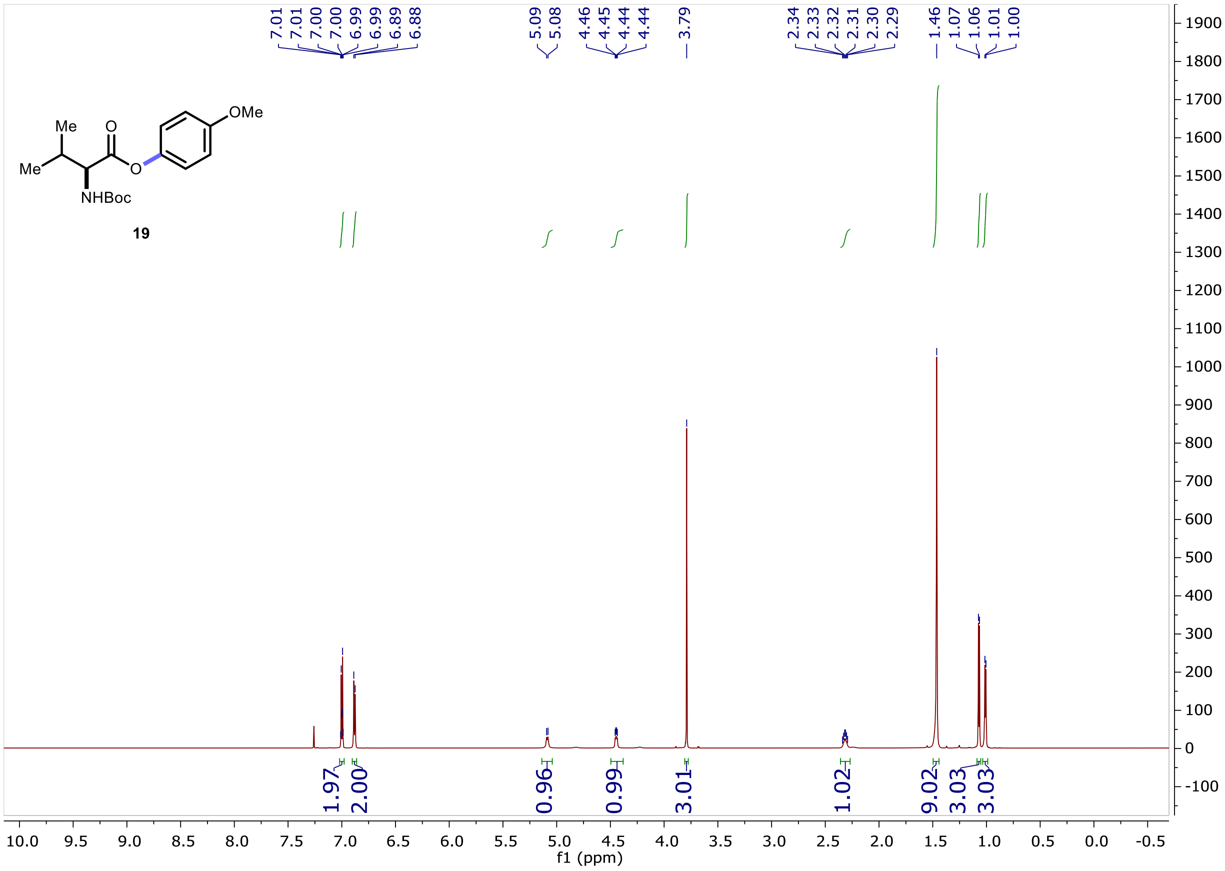

7.01
7.01
7.00
7.00
6.99
6.99
6.89
6.88
5.09
5.08
4.46
4.45
4.44
4.44
3.79
2.34
2.33
2.32
2.31
2.30
2.29
1.46
1.07
1.06
1.01
1.00
Me
O
OMe
Me
O
NHBoc
19
1.97
2.00
0.96
0.99
3.01
1.02
9.02
3.03
3.03
f1 (ppm)

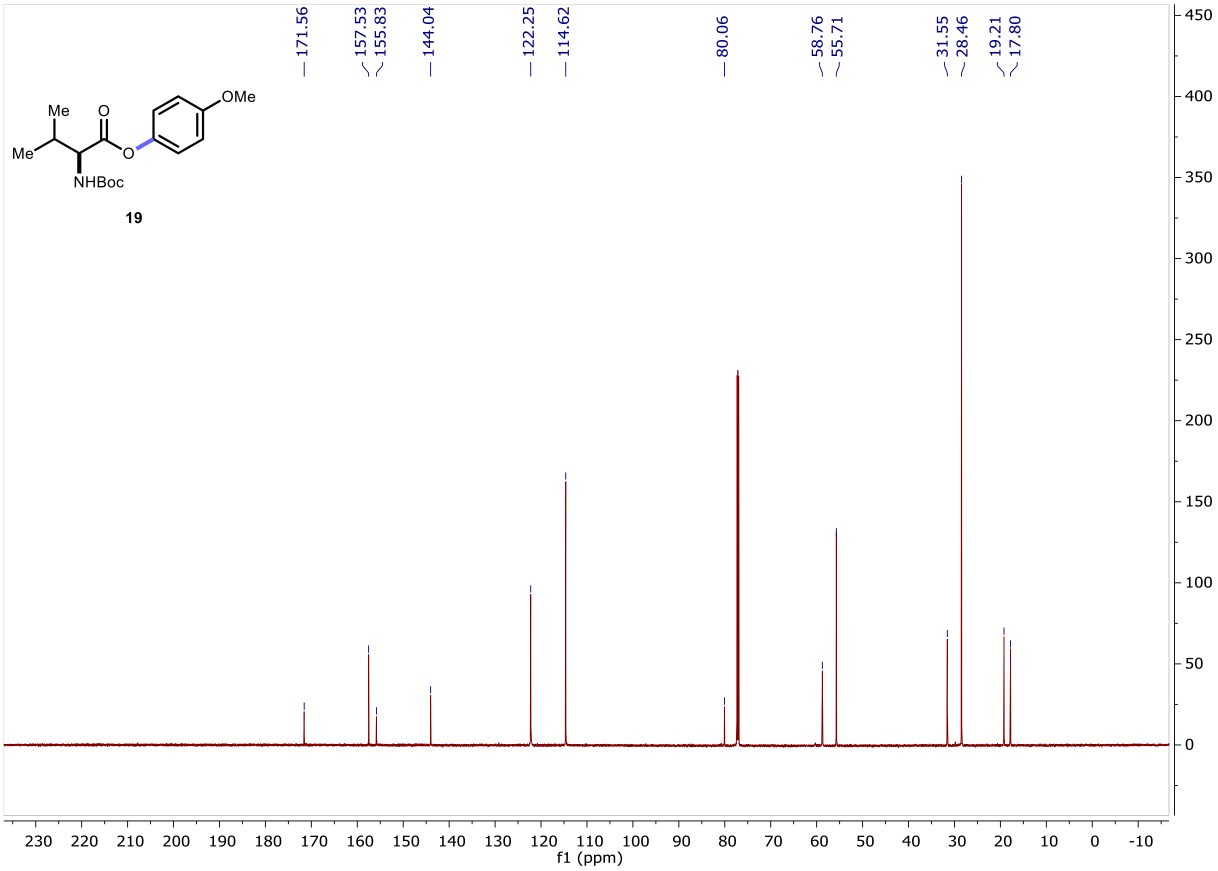
171.56
157.53
155.83
144.04
122.25
114.62
80.06
58.76
55.71
31.55
28.46
19.21
17.80
Me
O
OMe
Me
O
NHBoc
19
450
400
350
300
250
200
150
100
50
0
230 220 210 200 190 180 170 160 150 140 130 120 110 100 90 80 70 60 50 40 30 20 10 0 -10
f1 (ppm)

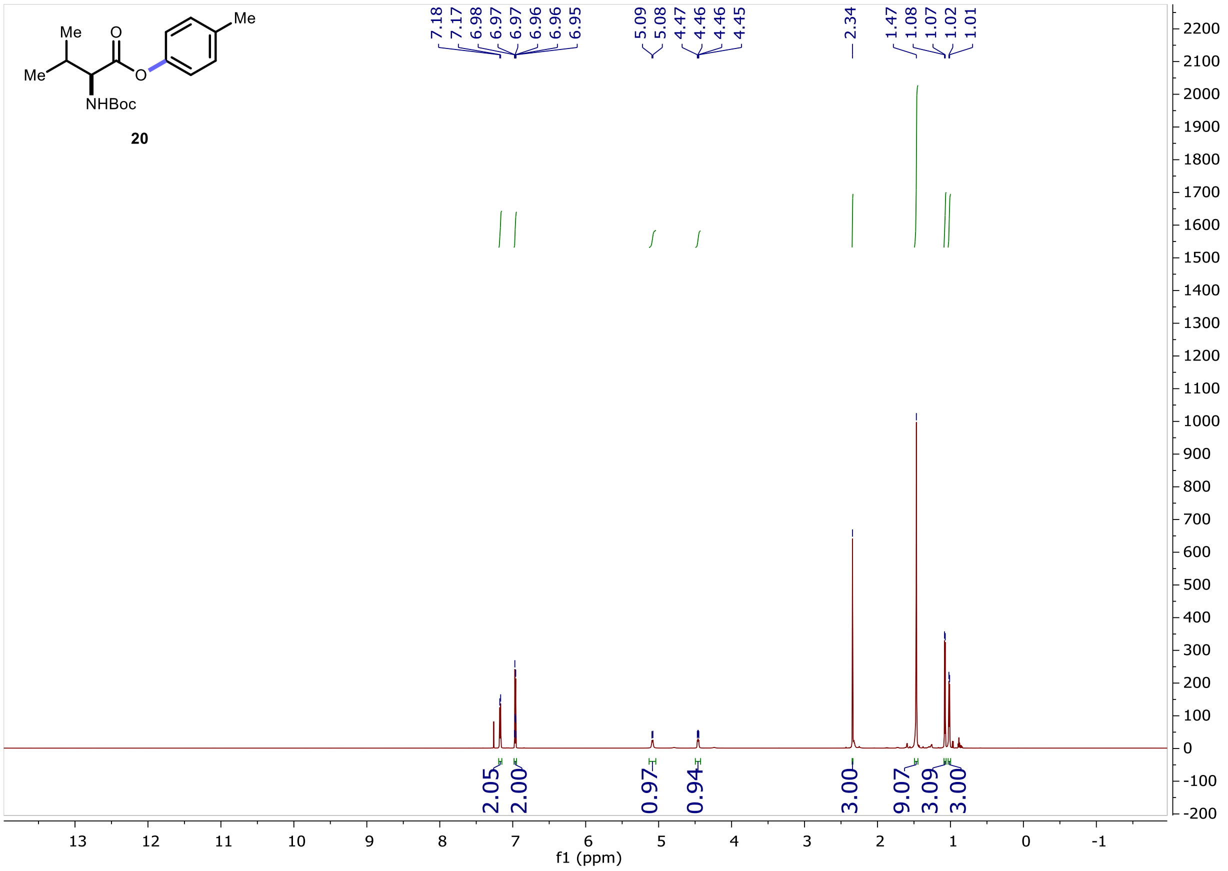

20
7.18
7.17
6.98
6.97
6.97
6.96
6.96
6.95
5.09
5.08
4.47
4.46
4.46
4.45
2.34
1.47
1.08
1.07
1.02
1.01
2.05
2.00
0.97
0.94
3.00
9.07
3.09
3.00
f1 (ppm)

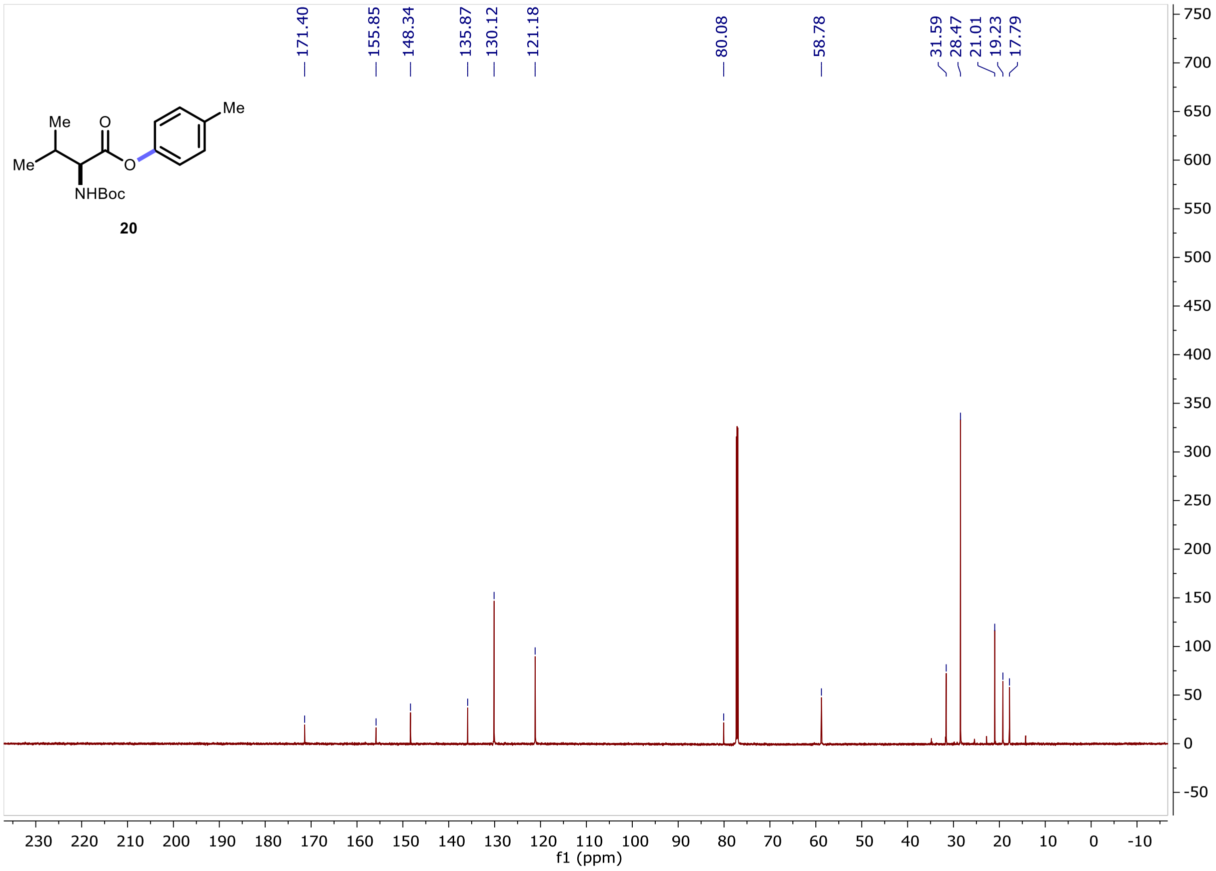

171.40
155.85
148.34
135.87
130.12
121.18
80.08
58.78
31.59
28.47
21.01
19.23
17.79
Me
O
Me
Me
O
NHBoc
20
f1 (ppm)

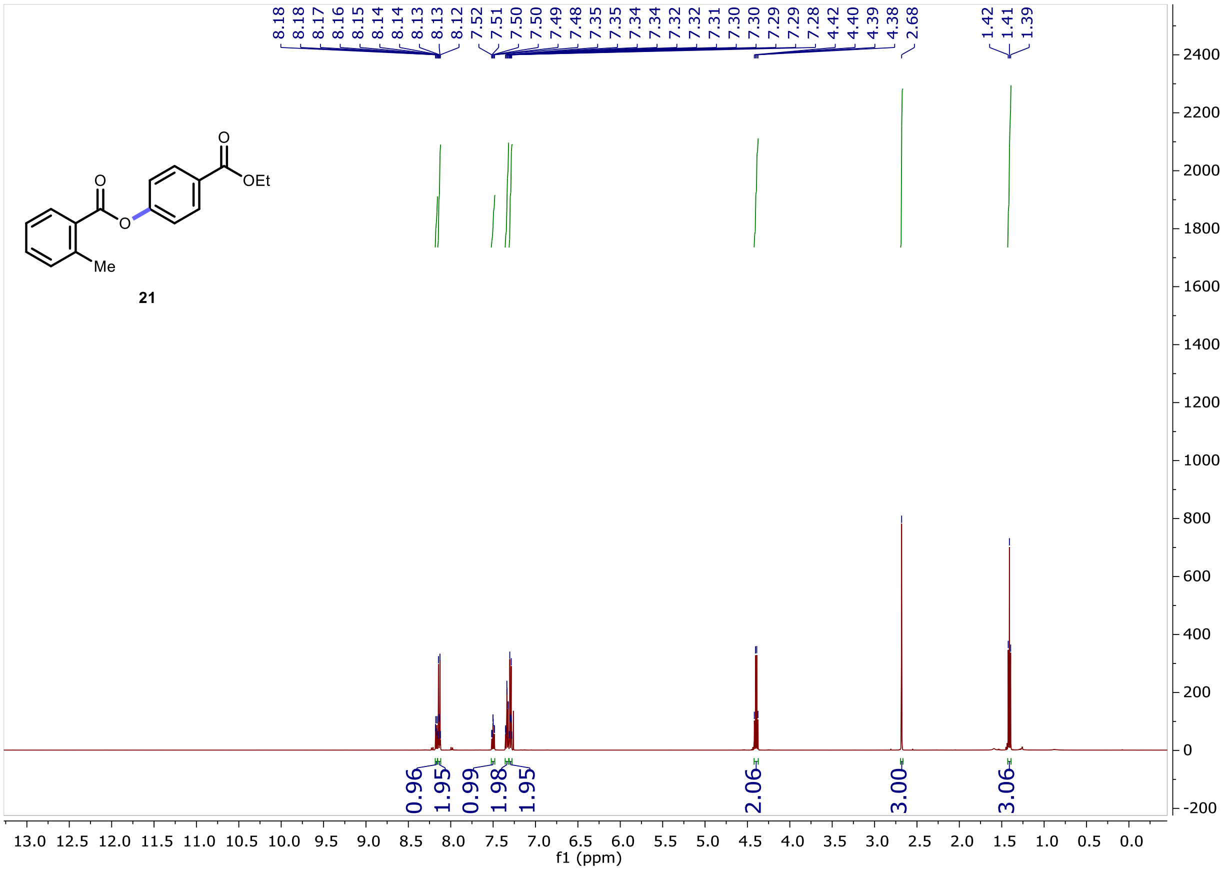

8.18
8.18
8.17
8.16
8.15
8.14
8.14
8.13
8.13
8.12
7.52
7.51
7.50
7.50
7.49
7.48
7.35
7.35
7.34
7.34
7.32
7.32
7.31
7.30
7.30
7.29
7.29
7.28
4.42
4.40
4.39
4.38
2.68
1.42
1.41
1.39
0.96
1.95
0.99
1.98
1.95
2.06
3.00
3.06
O
O
OEt
O
Me
21
13.0 12.5 12.0 11.5 11.0 10.5 10.0 9.5 9.0 8.5 8.0 7.5 7.0 6.5 6.0 5.5 5.0 4.5 4.0 3.5 3.0 2.5 2.0 1.5 1.0 0.5 0.0
f1 (ppm)
2400
2200
2000
1800
1600
1400
1200
1000
800
600
400
200
0
-200

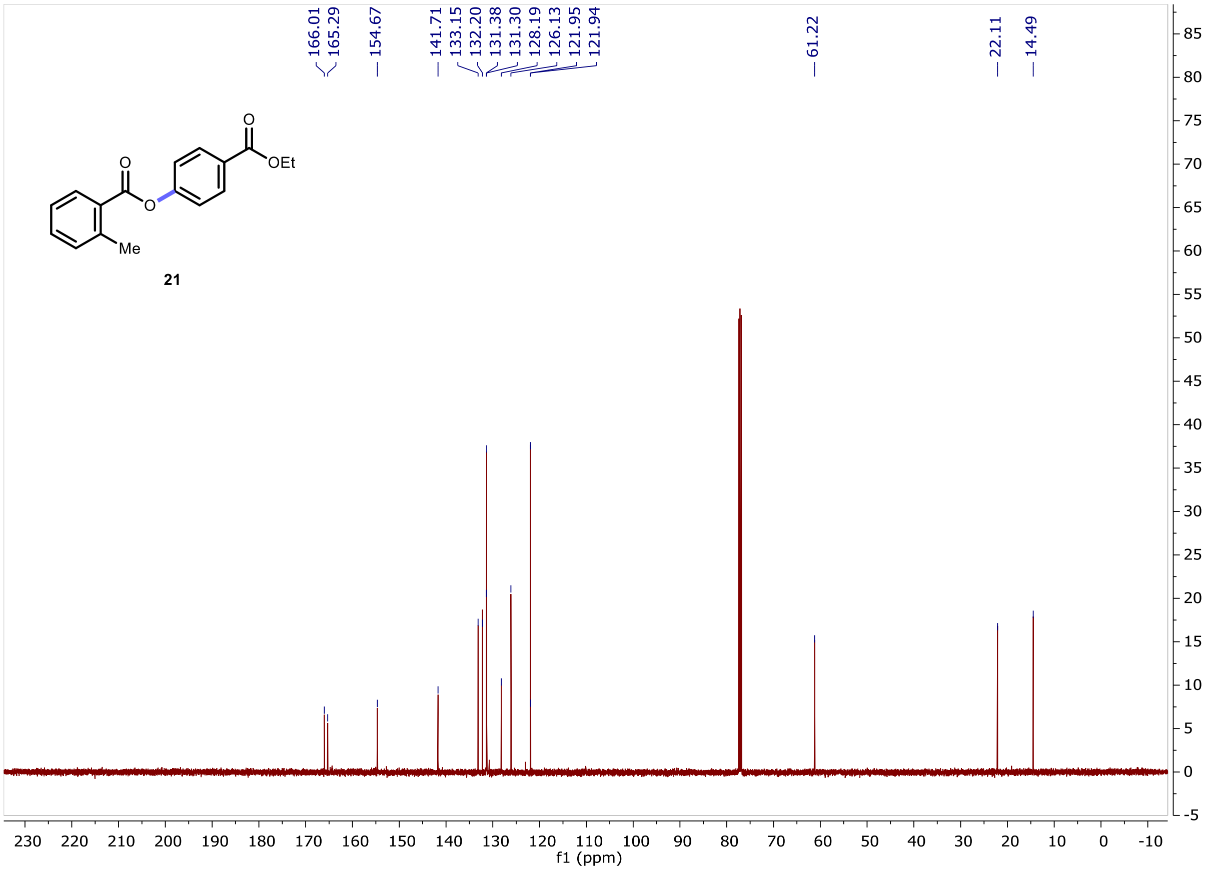
166.01
165.29
154.67
141.71
133.15
132.20
131.38
131.30
128.19
126.13
121.95
121.94
61.22
22.11
14.49
O
O
O
OEt
Me
21
f1 (ppm)

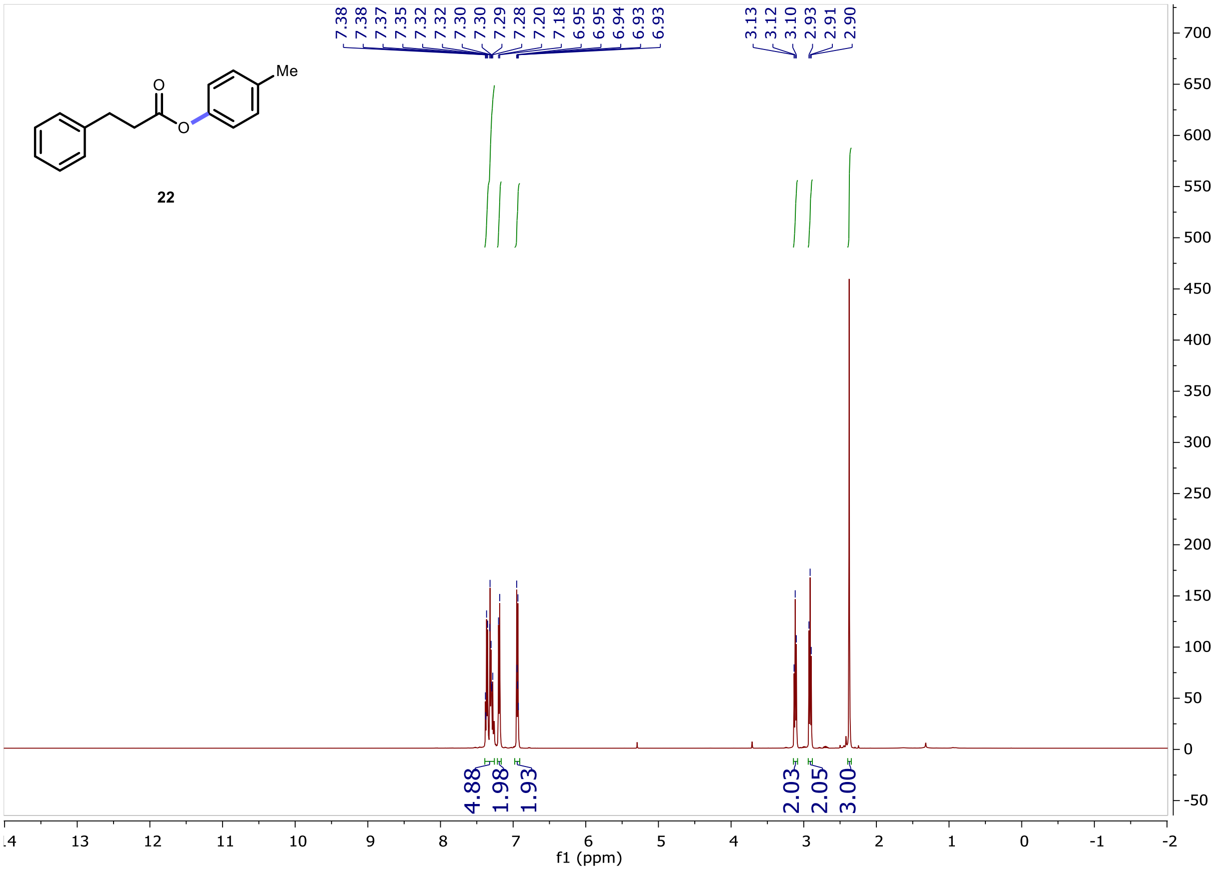

Me
O
O
22
7.38
7.38
7.37
7.35
7.32
7.32
7.30
7.30
7.29
7.28
7.20
7.18
6.95
6.95
6.94
6.93
6.93
3.13
3.12
3.10
2.93
2.91
2.90
4.88
1.98
1.93
2.03
2.05
3.00
f1 (ppm)

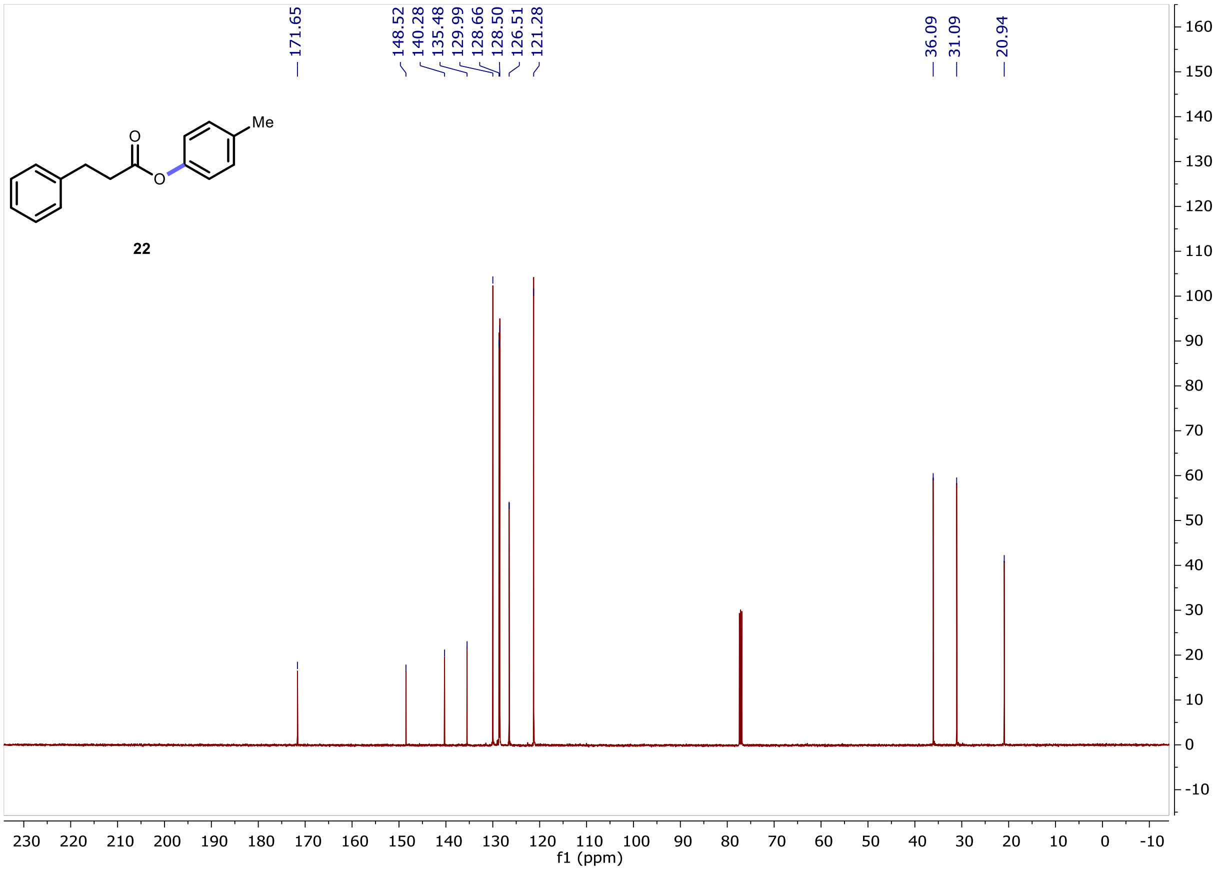
171.65
148.52
140.28
135.48
129.99
128.66
128.50
126.51
121.28
36.09
31.09
20.94
Me
O
O
22
f1 (ppm)

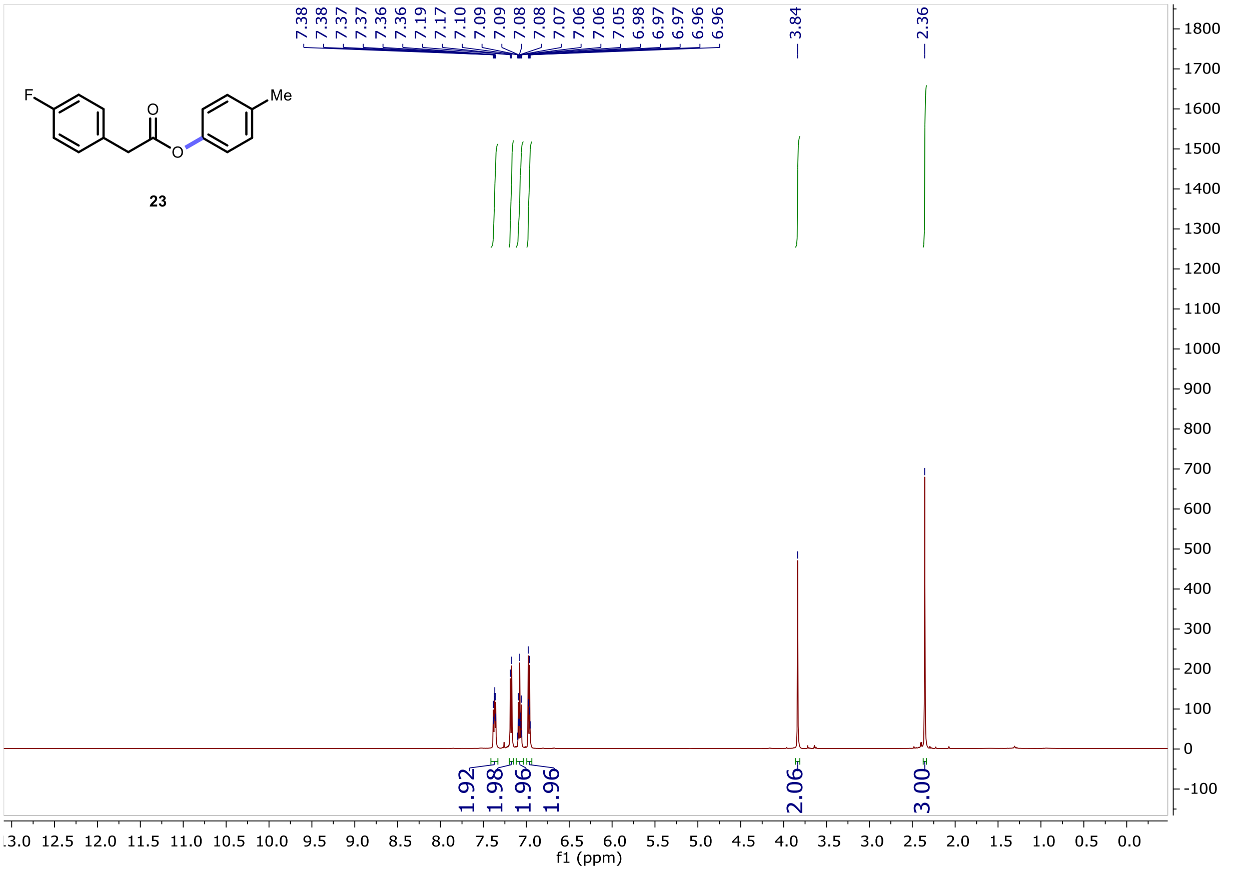
7.38
7.38
7.37
7.37
7.36
7.36
7.19
7.17
7.10
7.09
7.09
7.08
7.08
7.07
7.06
7.06
7.05
6.98
6.97
6.97
6.96
6.96
3.84
2.36
F
O
O
Me
23
1.92
1.98
1.96
1.96
2.06
3.00
f1 (ppm)

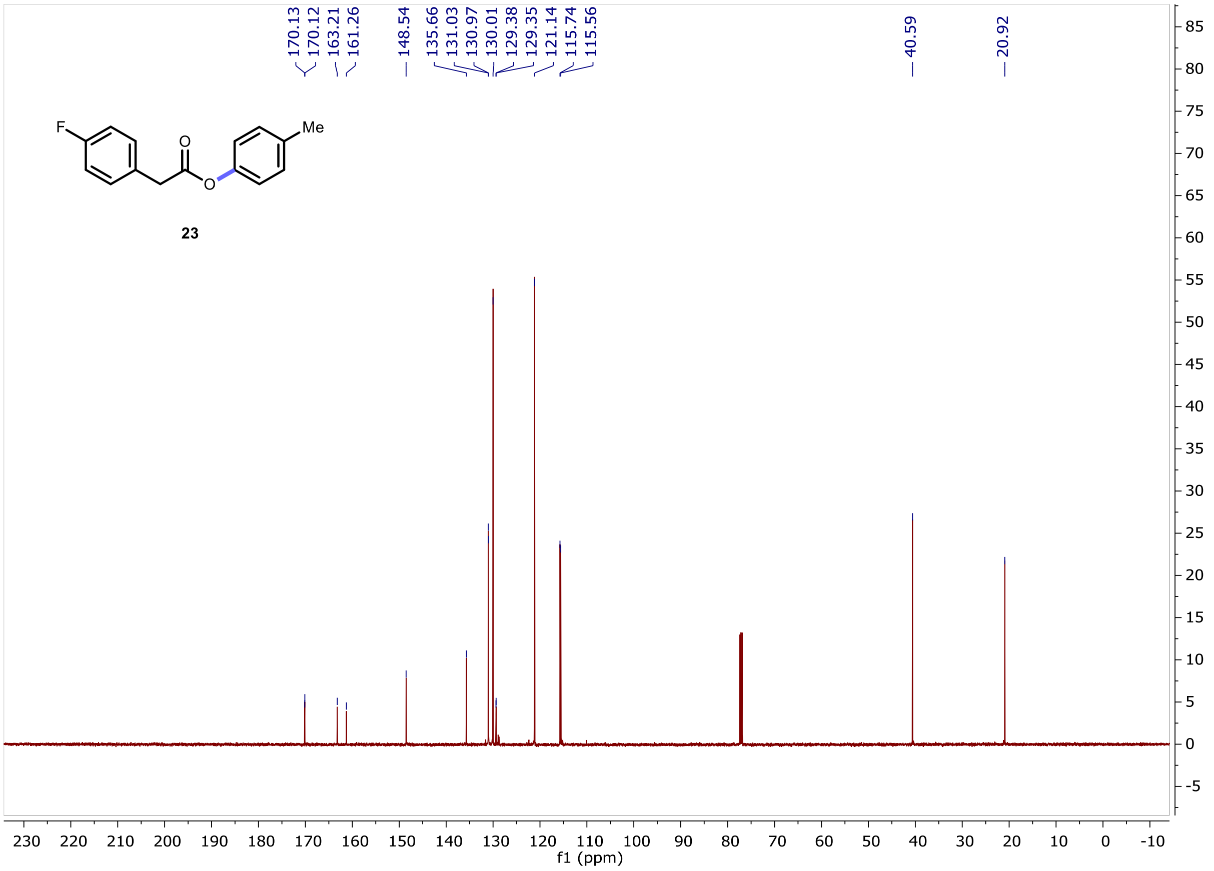
170.13
170.12
163.21
161.26
148.54
135.66
131.03
130.97
130.01
129.38
129.35
121.14
115.74
115.56
40.59
20.92
F
O
O
Me
23
f1 (ppm)

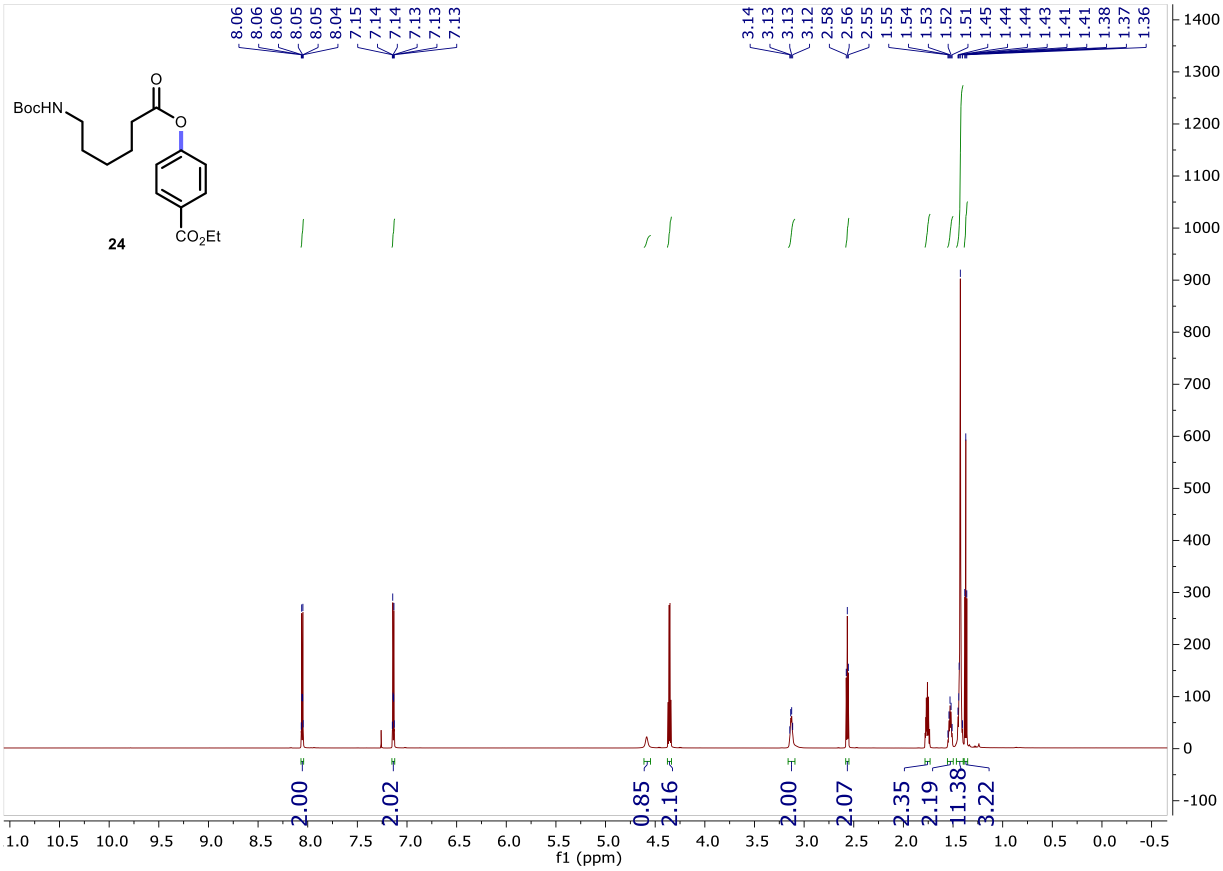
BocHN
O
O
CO2Et
24
8.06
8.06
8.06
8.05
8.05
8.04
7.15
7.14
7.14
7.13
7.13
7.13
3.14
3.13
3.13
3.12
2.58
2.56
2.55
1.55
1.54
1.53
1.52
1.51
1.45
1.44
1.44
1.43
1.41
1.41
1.38
1.37
1.36
2.00
2.02
0.85
2.16
2.00
2.07
2.35
2.19
11.38
3.22
f1 (ppm)

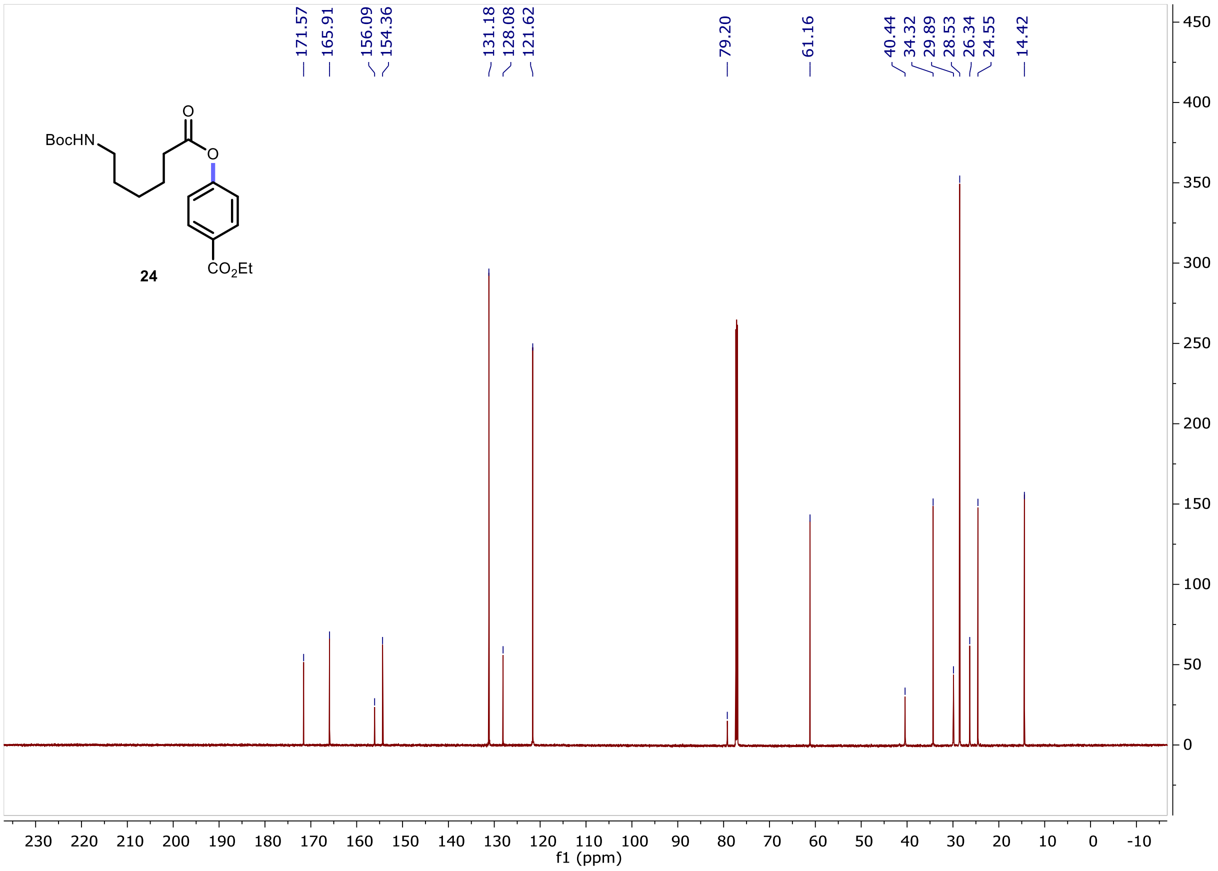
171.57
165.91
156.09
154.36
131.18
128.08
121.62
79.20
61.16
40.44
34.32
29.89
28.53
26.34
24.55
14.42
BocHN
O
O
CO2Et
24
f1 (ppm)

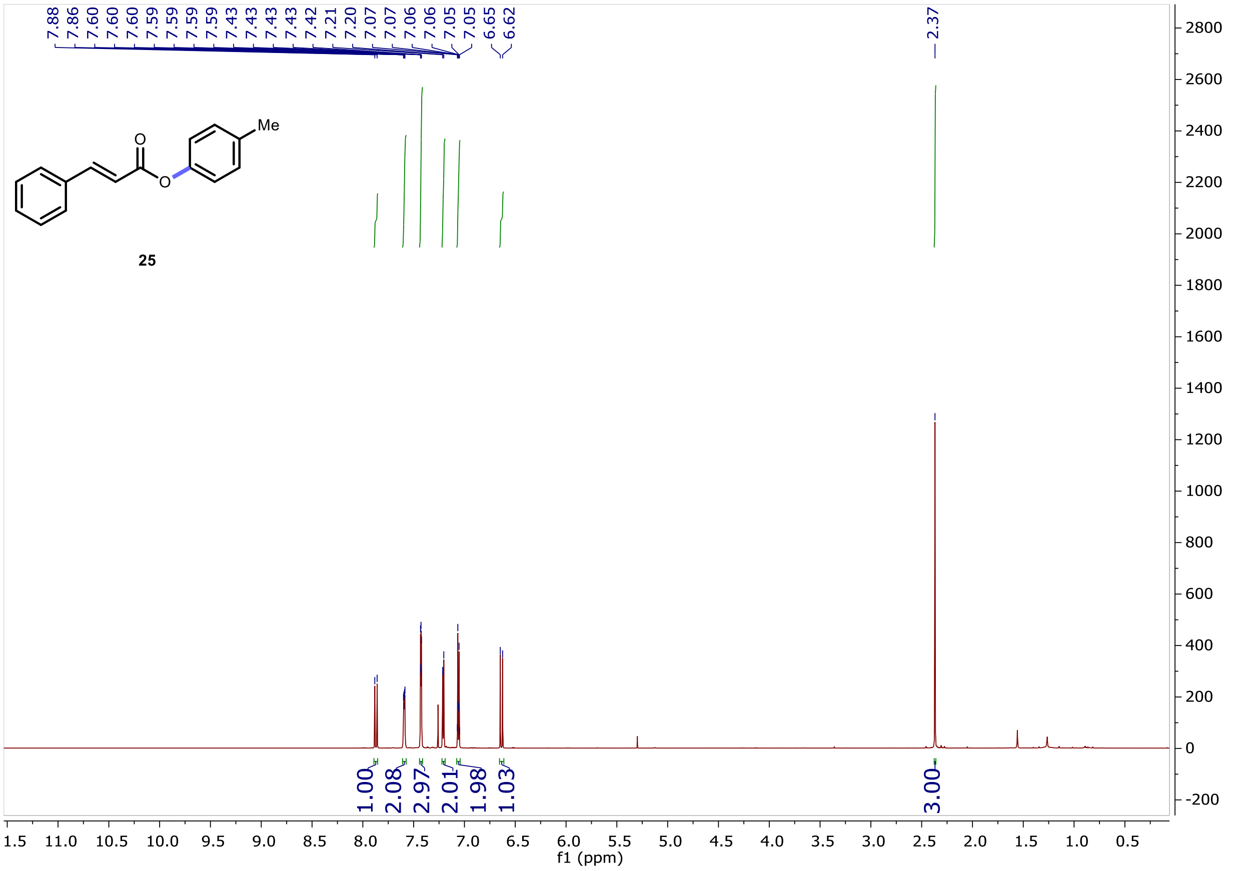

7.88
7.86
7.60
7.60
7.60
7.59
7.59
7.59
7.59
7.43
7.43
7.43
7.43
7.42
7.21
7.20
7.07
7.07
7.06
7.06
7.05
7.05
6.65
6.62
2.37
Me
O
O
25
1.00
2.08
2.97
2.01
1.98
1.03
3.00
f1 (ppm)

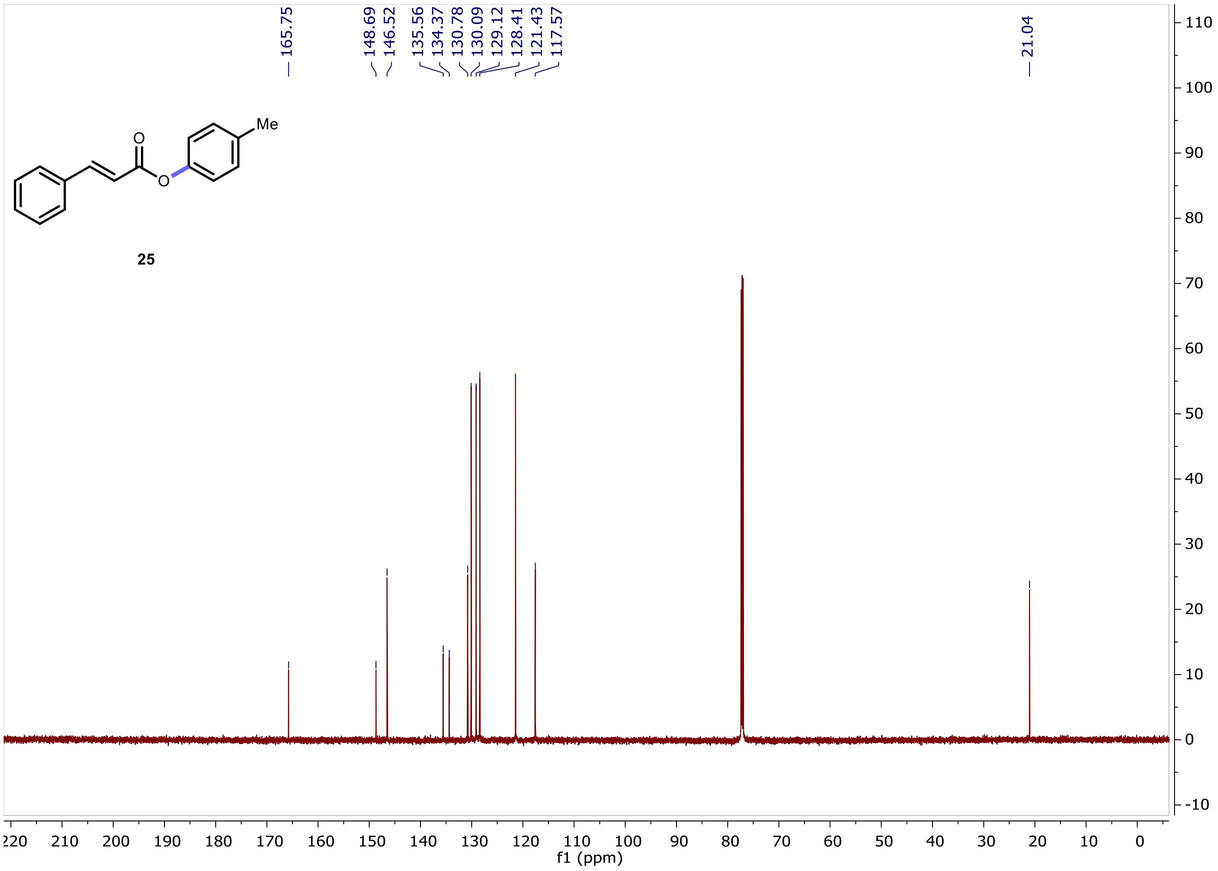
165.75
148.69
146.52
135.56
134.37
130.78
130.09
129.12
128.41
121.43
117.57
21.04
Me
O
O
25
f1 (ppm)

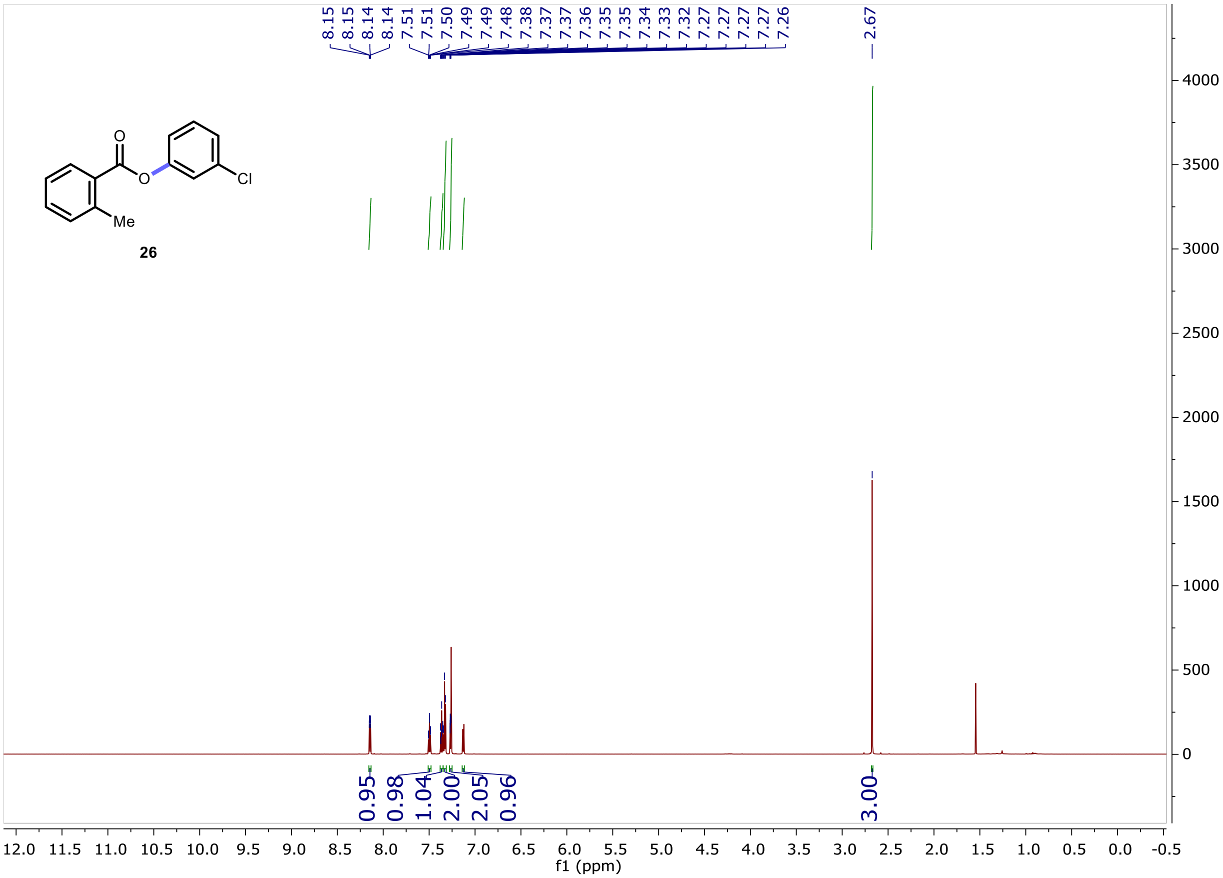

8.15
8.15
8.14
8.14
7.51
7.51
7.50
7.49
7.49
7.48
7.38
7.37
7.37
7.36
7.35
7.35
7.34
7.33
7.32
7.27
7.27
7.27
7.27
7.26
2.67
O
O
Cl
Me
26
0.95
0.98
1.04
2.00
2.05
0.96
3.00
12.0 11.5 11.0 10.5 10.0 9.5 9.0 8.5 8.0 7.5 7.0 6.5 6.0 5.5 5.0 4.5 4.0 3.5 3.0 2.5 2.0 1.5 1.0 0.5 0.0 -0.5
f1 (ppm)
4000
3500
3000
2500
2000
1500
1000
500
0

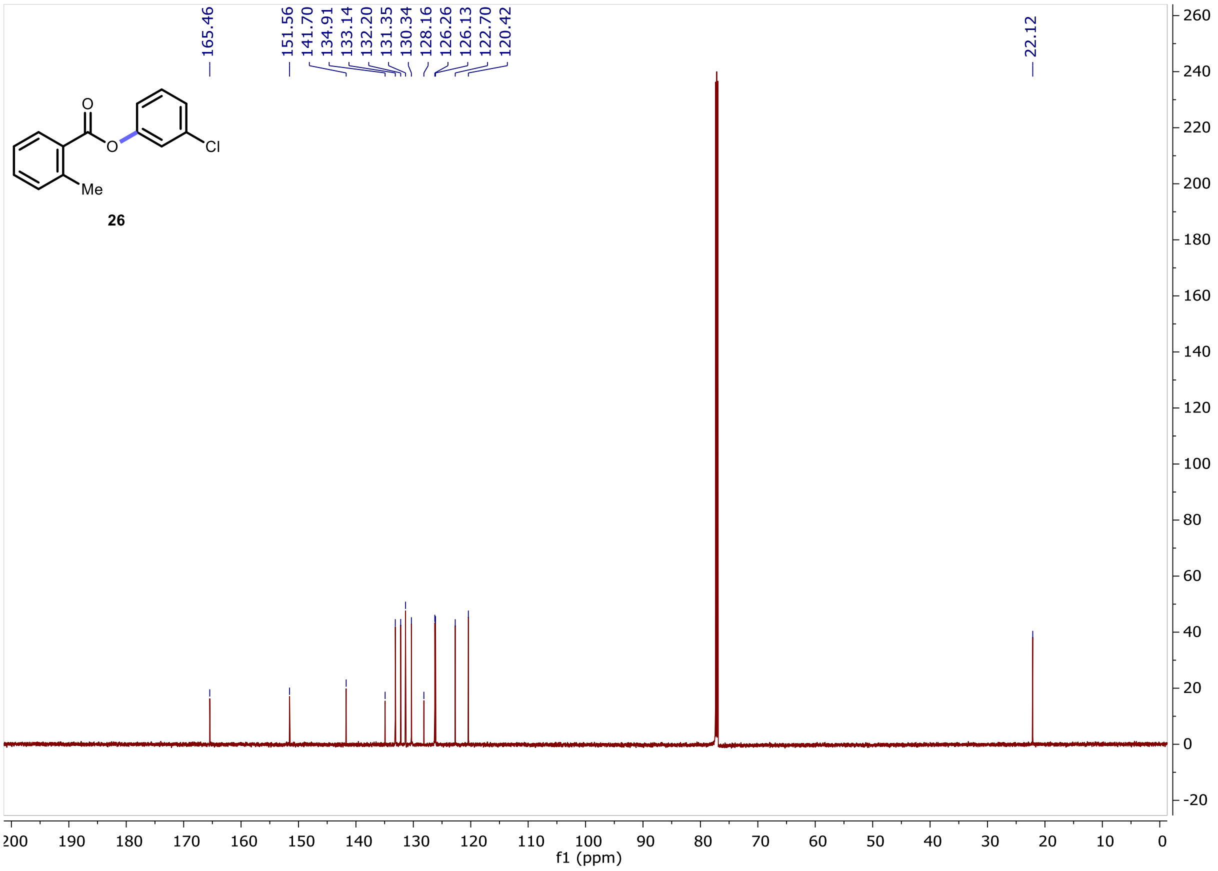
165.46
151.56
141.70
134.91
133.14
132.20
131.35
130.34
128.16
126.26
126.13
122.70
120.42
22.12
O
O
Cl
Me
26
f1 (ppm)

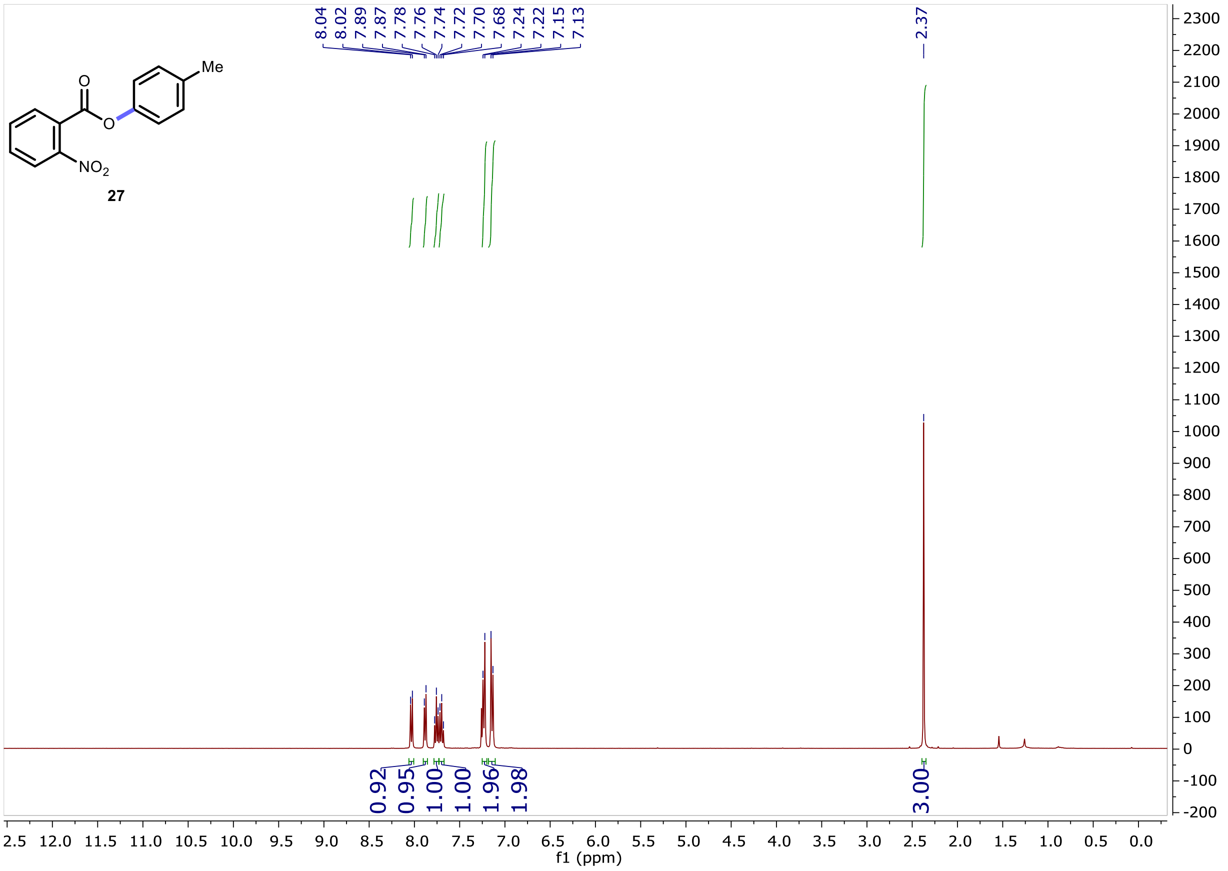
O
O
Me
NO2
27
8.04
8.02
7.89
7.87
7.78
7.76
7.74
7.72
7.70
7.68
7.24
7.22
7.15
7.13
2.37
0.92
0.95
1.00
1.00
1.96
1.98
3.00
f1 (ppm)

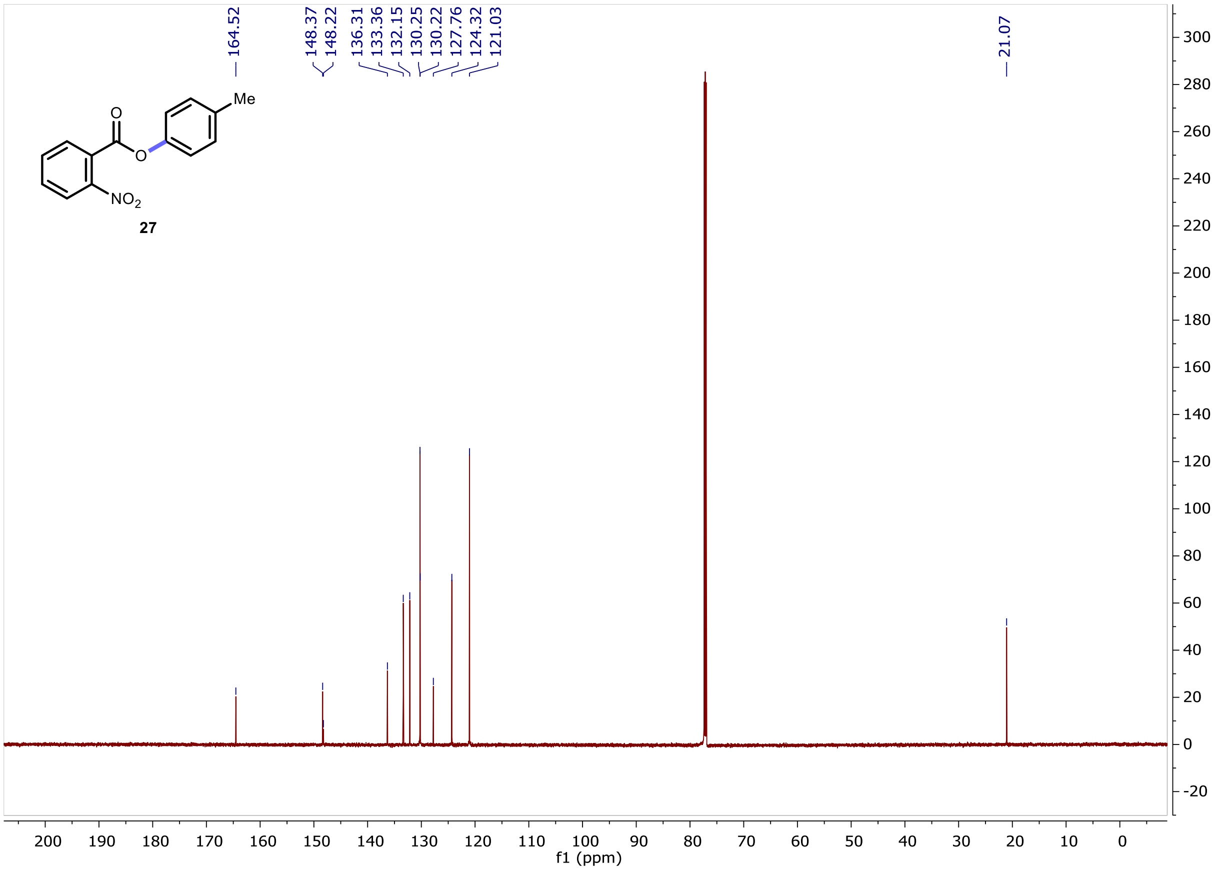
164.52
148.37
148.22
136.31
133.36
132.15
130.25
130.22
127.76
124.32
121.03
21.07
Me
O
O
NO2
27
f1 (ppm)

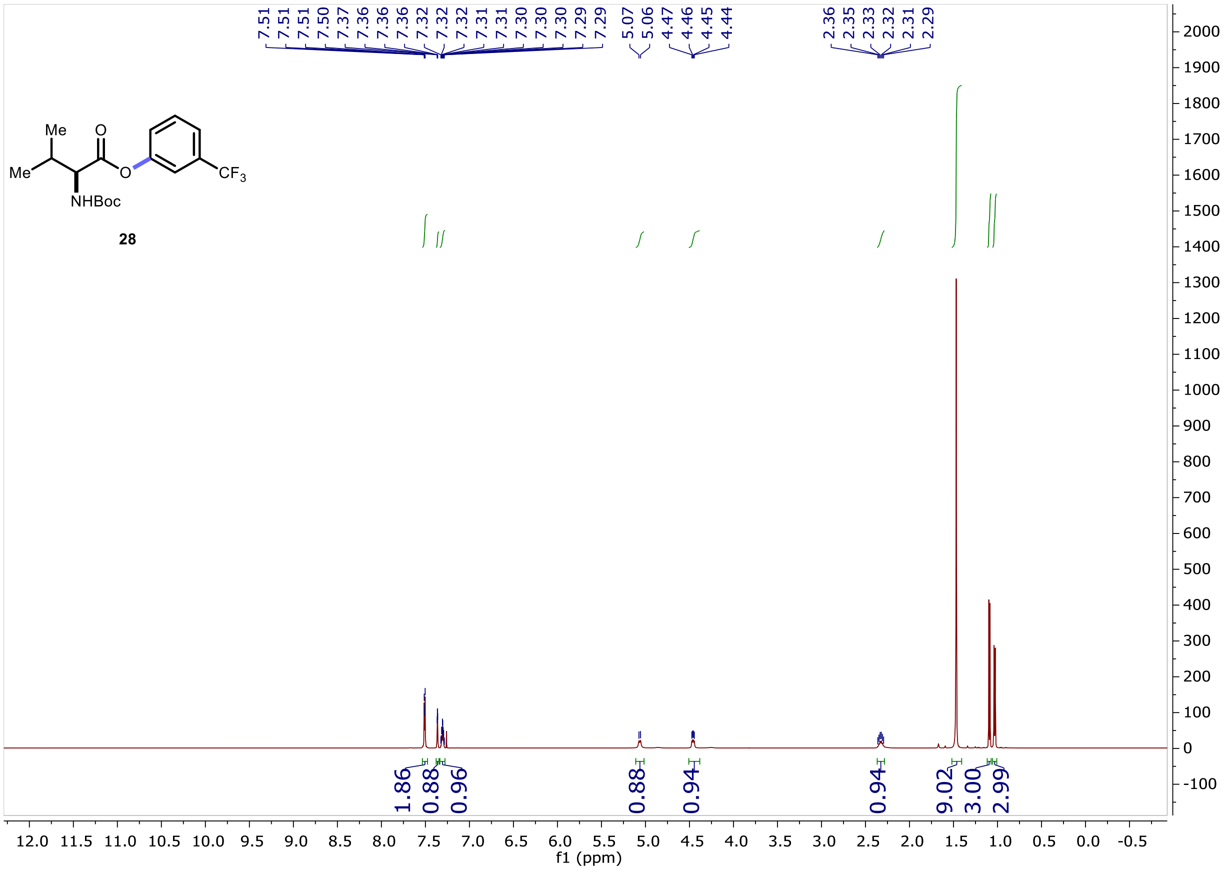

7.51
7.51
7.51
7.50
7.37
7.36
7.36
7.36
7.32
7.32
7.32
7.31
7.31
7.30
7.30
7.30
7.29
7.29
5.07
5.06
4.47
4.46
4.45
4.44
2.36
2.35
2.33
2.32
2.31
2.29
Me
O
Me
O
CF3
NHBoc
28
1.86
0.88
0.96
0.88
0.94
0.94
9.02
3.00
2.99
f1 (ppm)

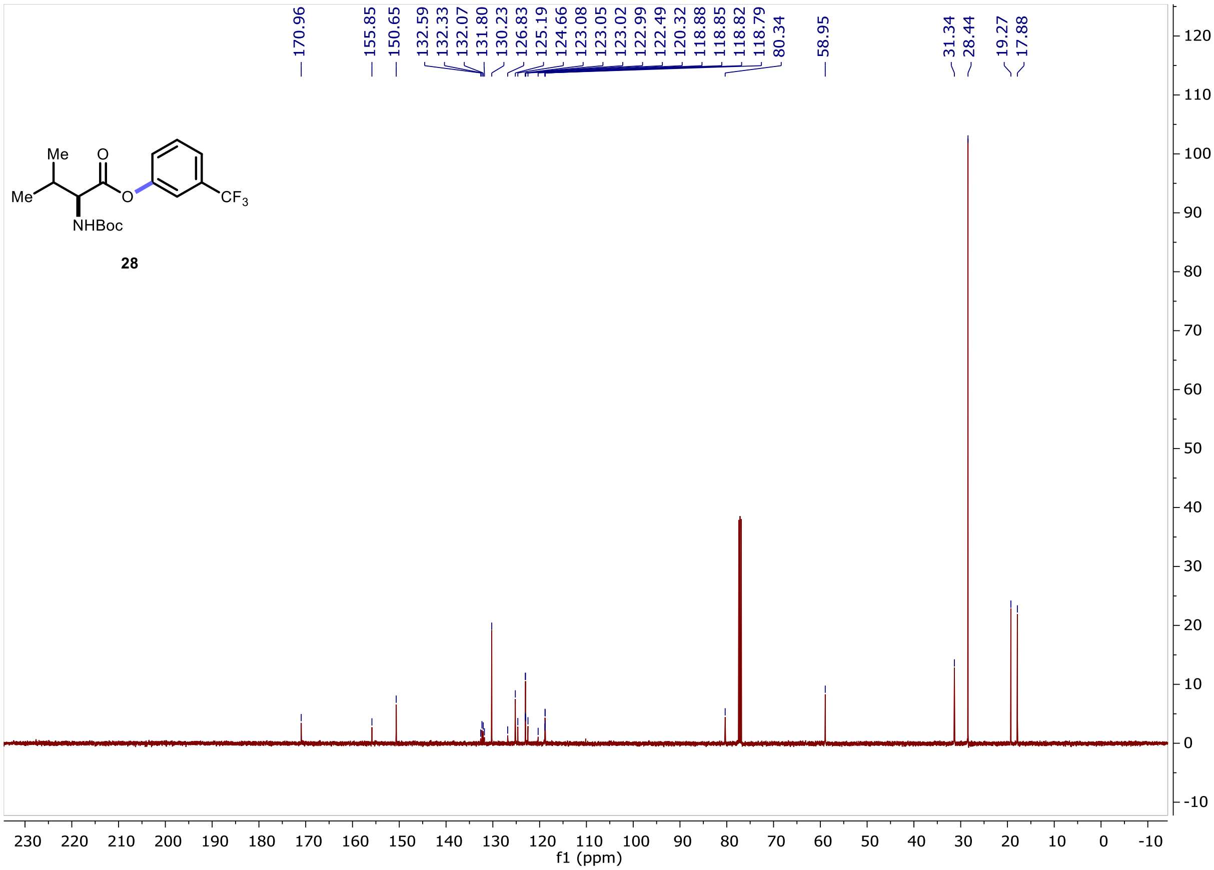
170.96
155.85
150.65
132.59
132.33
132.07
131.80
130.23
126.83
125.19
124.66
123.08
123.05
123.02
122.99
122.49
120.32
118.88
118.85
118.82
118.79
80.34
58.95
31.34
28.44
19.27
17.88
Me
O
Me
O
CF3
NHBoc
28
f1 (ppm)

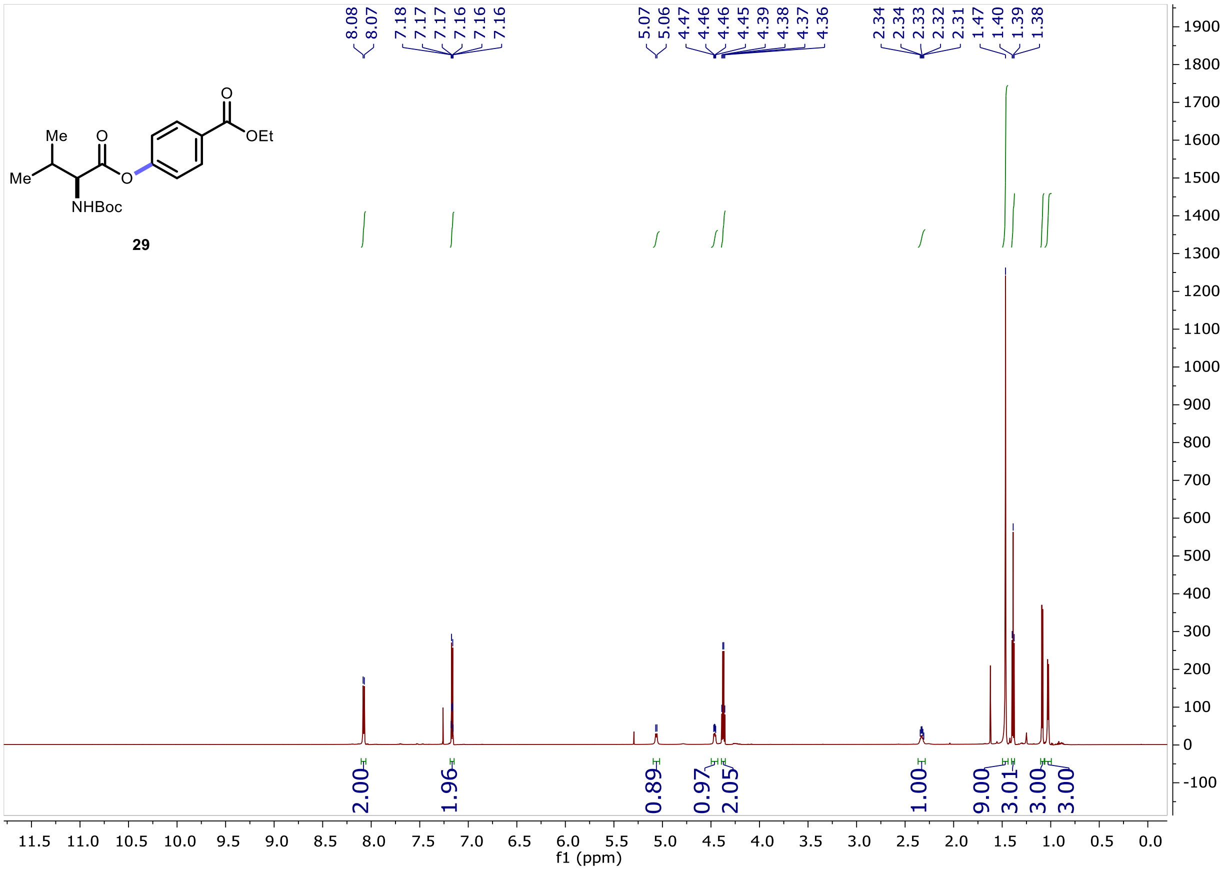

Me
O
O
OEt
Me
O
NHBoc
8.08
8.07
7.18
7.17
7.17
7.16
7.16
7.16
5.07
5.06
4.47
4.46
4.46
4.45
4.39
4.38
4.37
4.36
2.34
2.34
2.33
2.32
2.31
1.47
1.40
1.39
1.38
2.00
1.96
0.89
0.97
2.05
1.00
9.00
3.01
3.00
3.00
f1 (ppm)

29

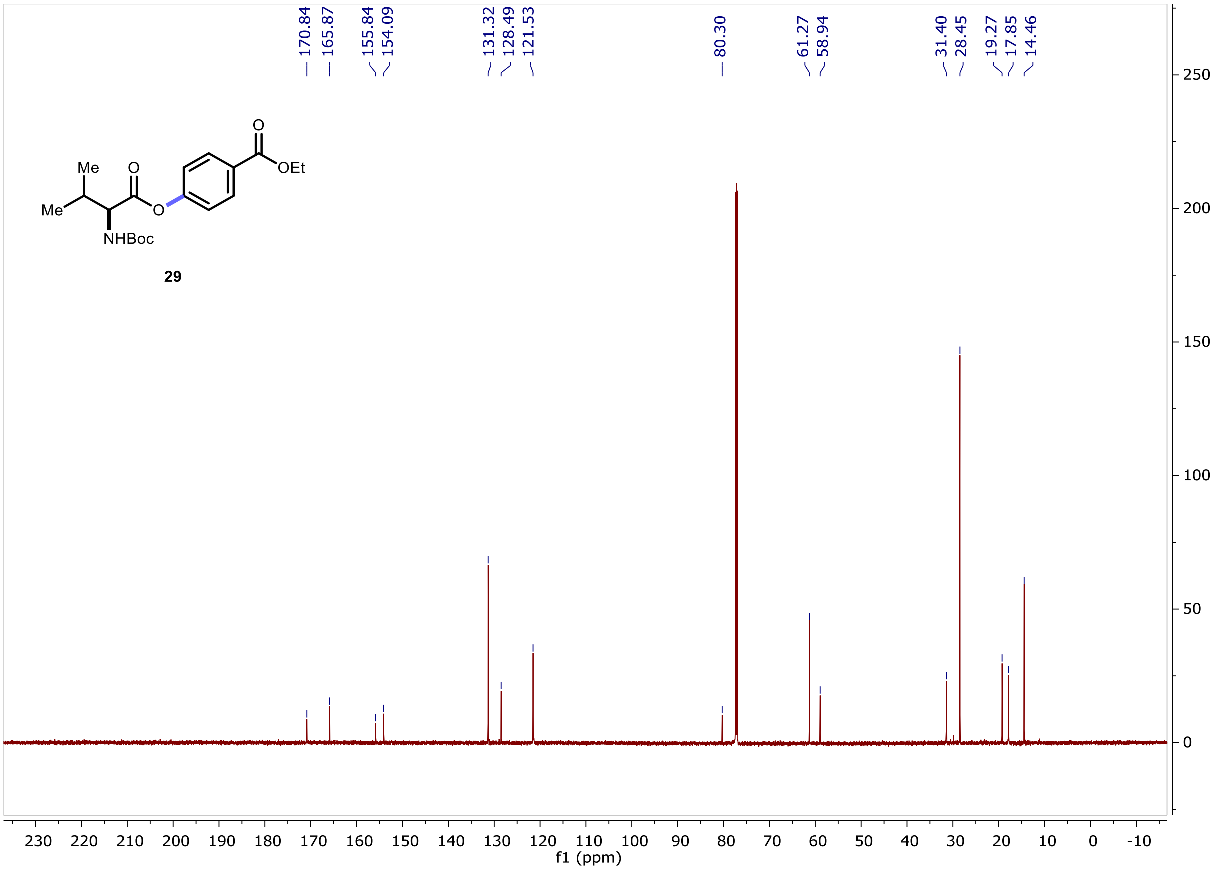
170.84
165.87
155.84
154.09
131.32
128.49
121.53
80.30
61.27
58.94
31.40
28.45
19.27
17.85
14.46
Me
O
O
OEt
Me
O
NHBoc
29
250
200
150
100
50
0
230 220 210 200 190 180 170 160 150 140 130 120 110 100 90 80 70 60 50 40 30 20 10 0 -10
f1 (ppm)

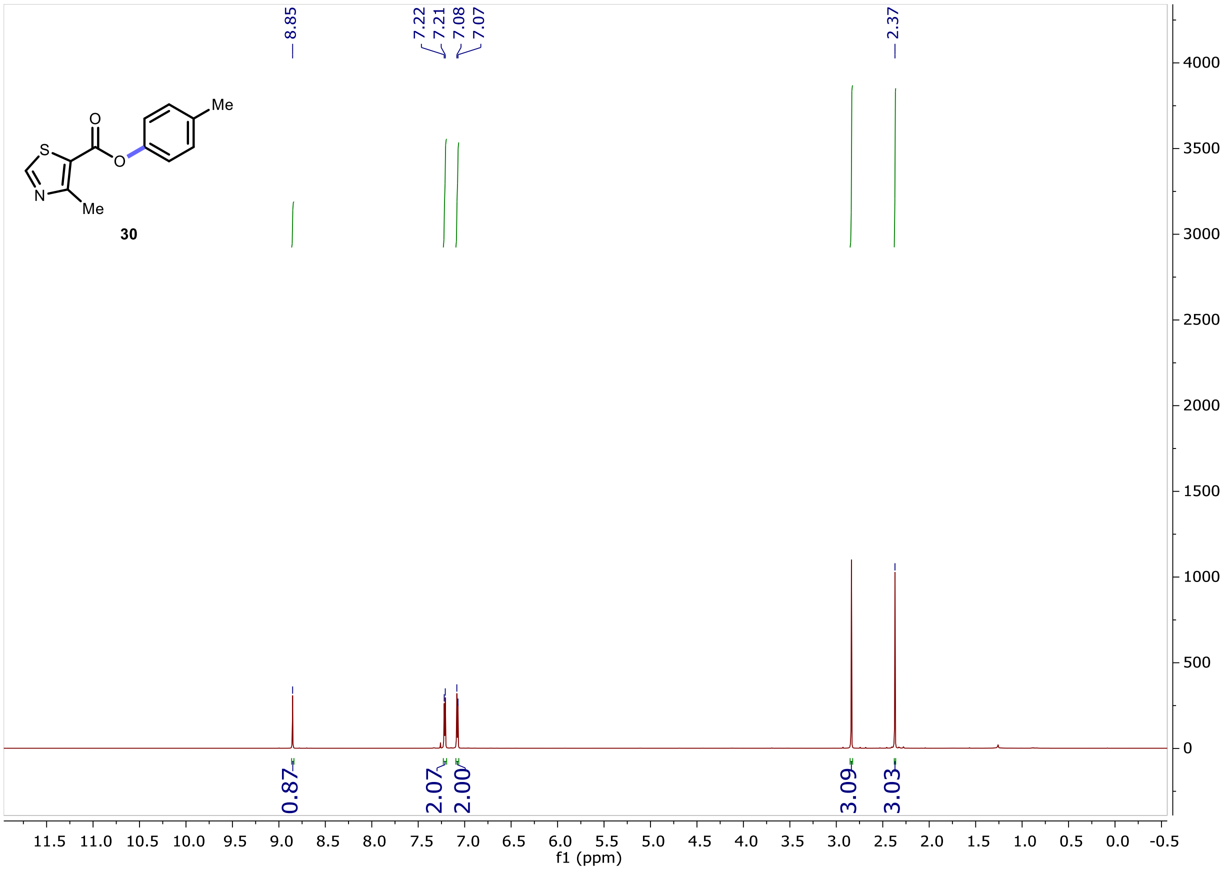

8.85
7.22
7.21
7.08
7.07
2.37
Me
O
S
O
N
Me
30
0.87
2.07
2.00
3.09
3.03
f1 (ppm)

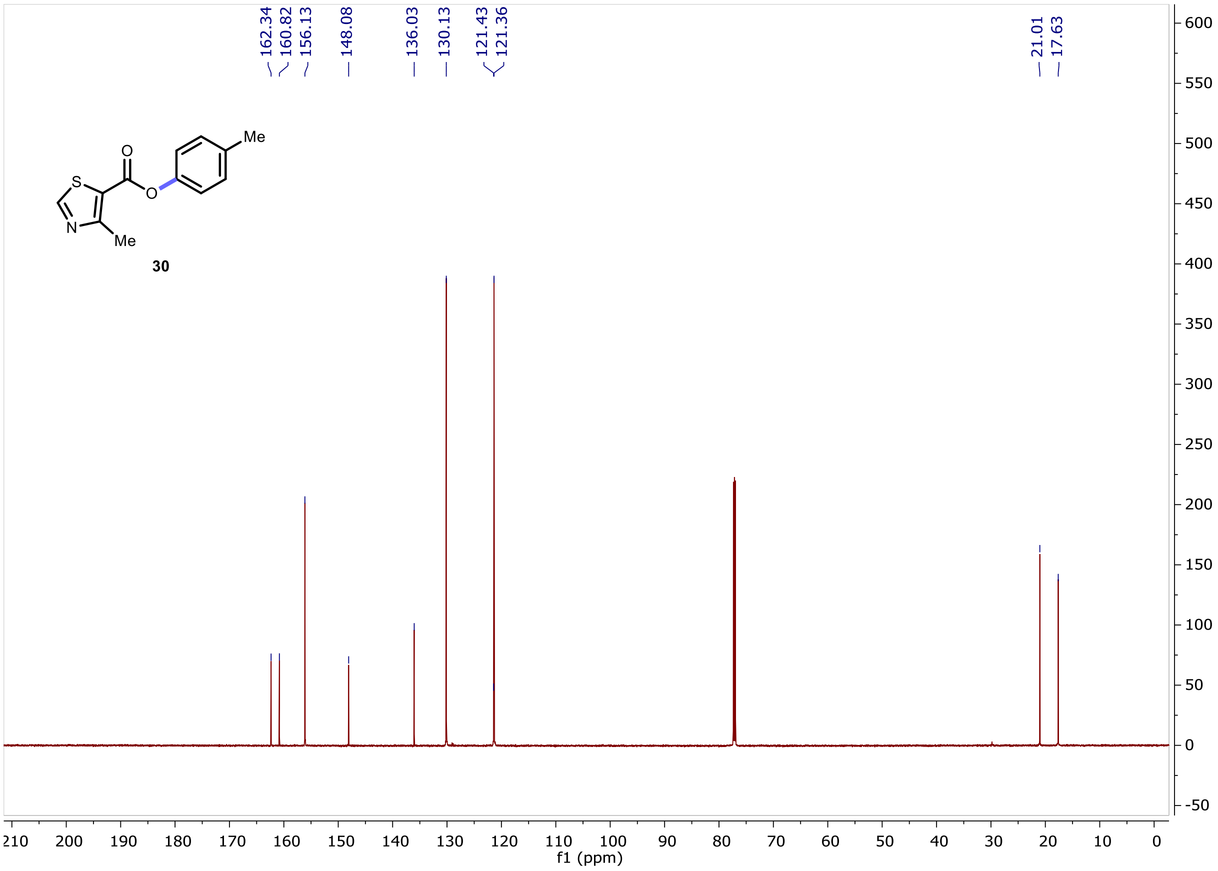
162.34
160.82
156.13
148.08
136.03
130.13
121.43
121.36
21.01
17.63
Me
O
S
O
N
Me
30
600
550
500
450
400
350
300
250
200
150
100
50
0
-50
210 200 190 180 170 160 150 140 130 120 110 100 90 80 70 60 50 40 30 20 10 0
f1 (ppm)

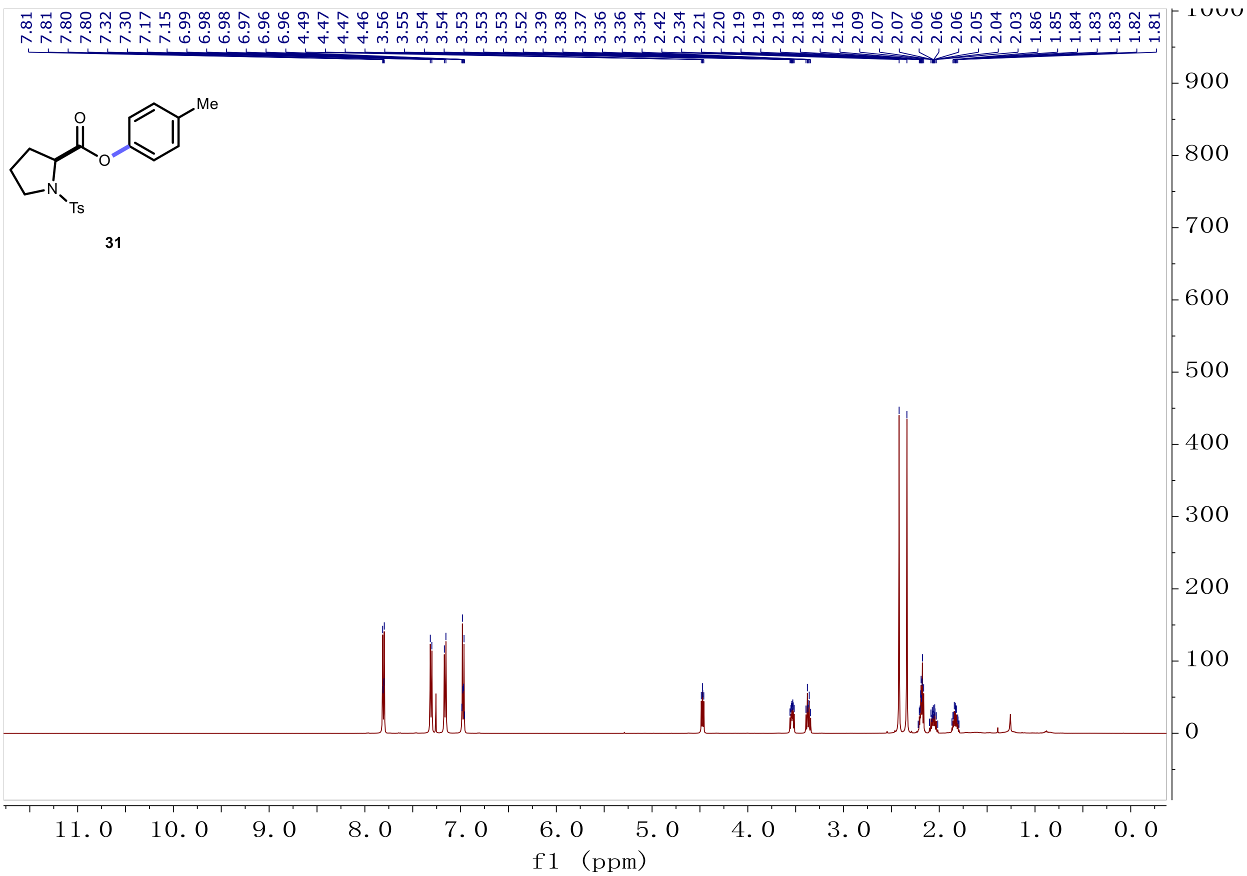
7.81
7.81
7.80
7.80
7.32
7.30
7.17
7.15
6.99
6.98
6.98
6.97
6.96
6.96
4.49
4.47
4.47
4.46
3.56
3.55
3.54
3.54
3.53
3.53
3.53
3.52
3.39
3.38
3.37
3.36
3.36
3.34
2.42
2.34
2.21
2.20
2.19
2.19
2.19
2.18
2.18
2.16
2.09
2.07
2.07
2.06
2.06
2.06
2.05
2.04
2.03
1.86
1.85
1.84
1.83
1.83
1.82
1.81
Me
O
O
N
Ts
31
f1 (ppm)

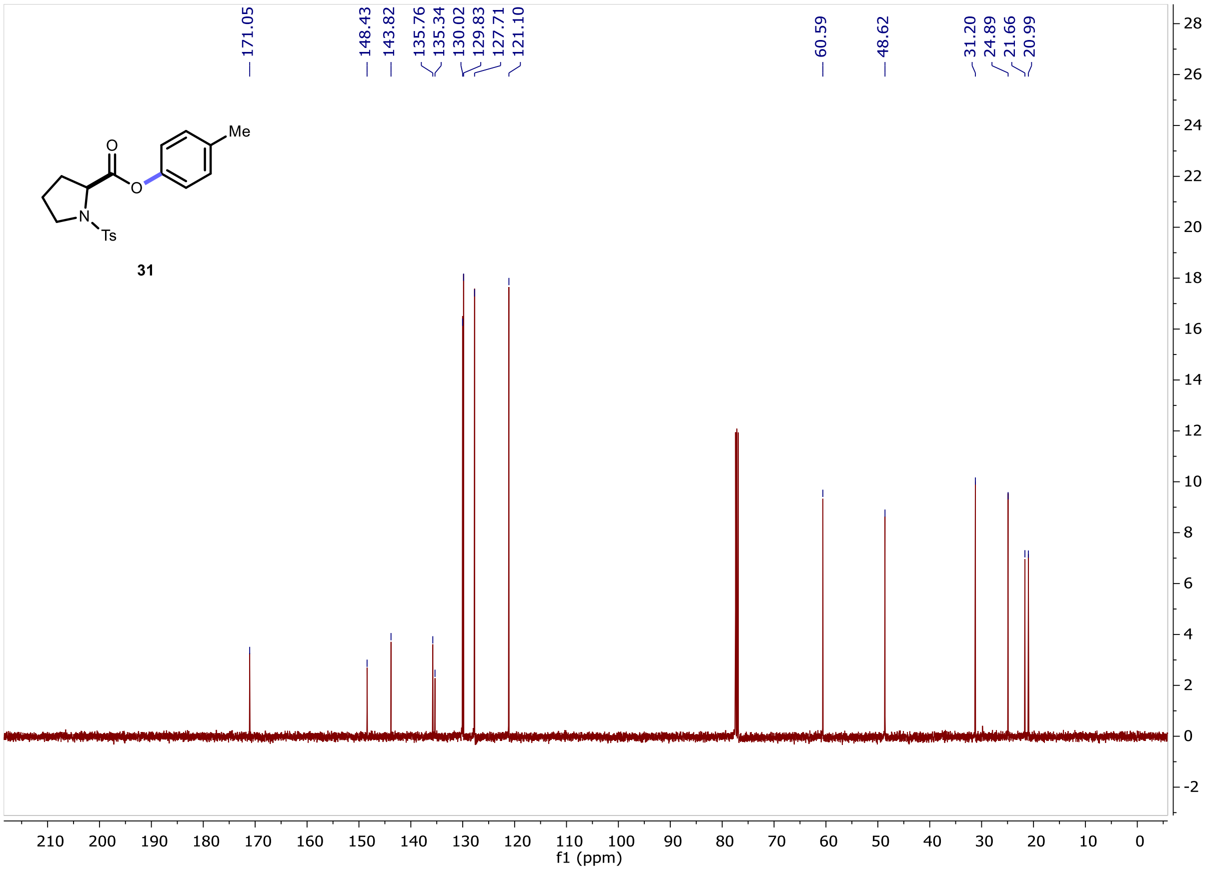
171.05
148.43
143.82
135.76
135.34
130.02
129.83
127.71
121.10
60.59
48.62
31.20
24.89
21.66
20.99
O
O
Me
N
Ts
31
28
26
24
22
20
18
16
14
12
10
8
6
4
2
0
-2
210
200
190
180
170
160
150
140
130
120
110
100
90
80
70
60
50
40
30
20
10
0
f1 (ppm)

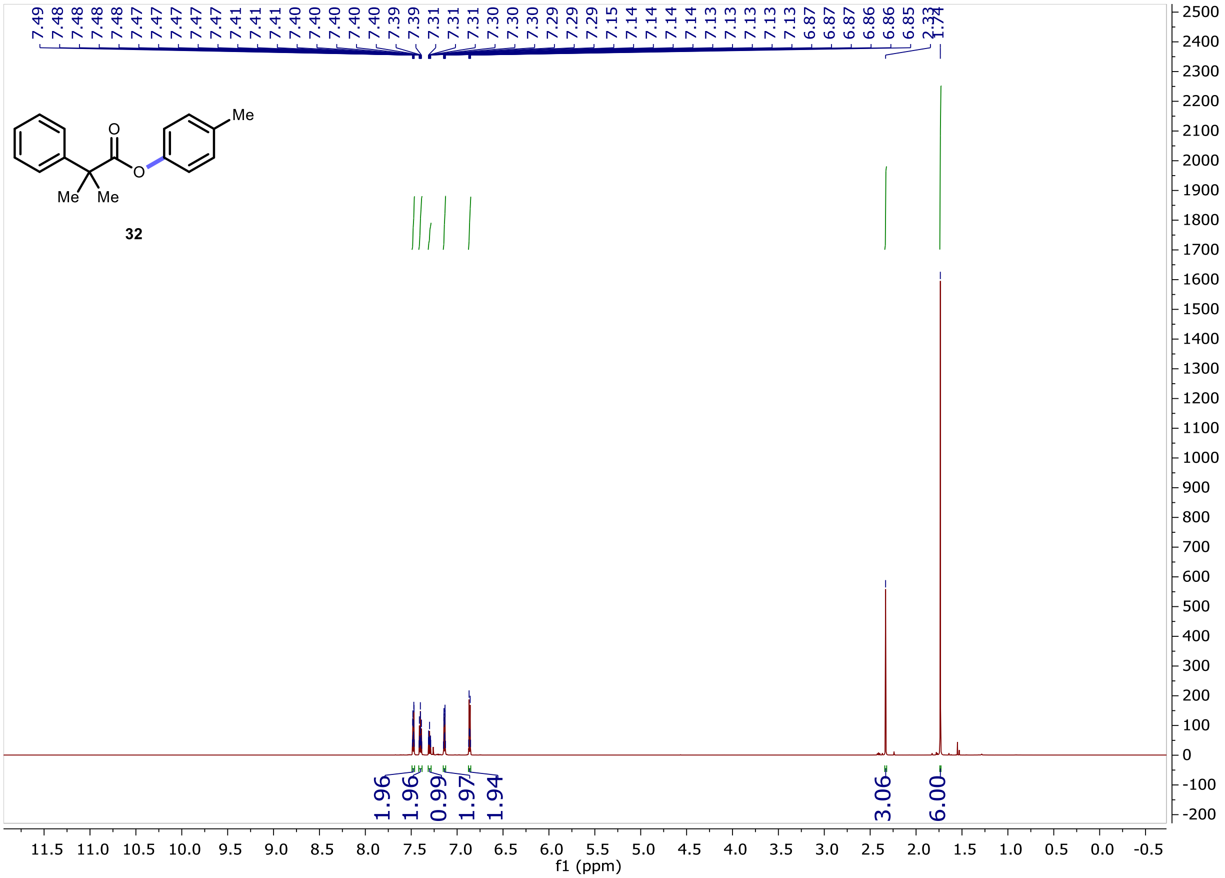
Me
O
O
Me
Me
32
f1 (ppm)
1.96
1.96
0.99
1.97
1.94
3.06
6.00

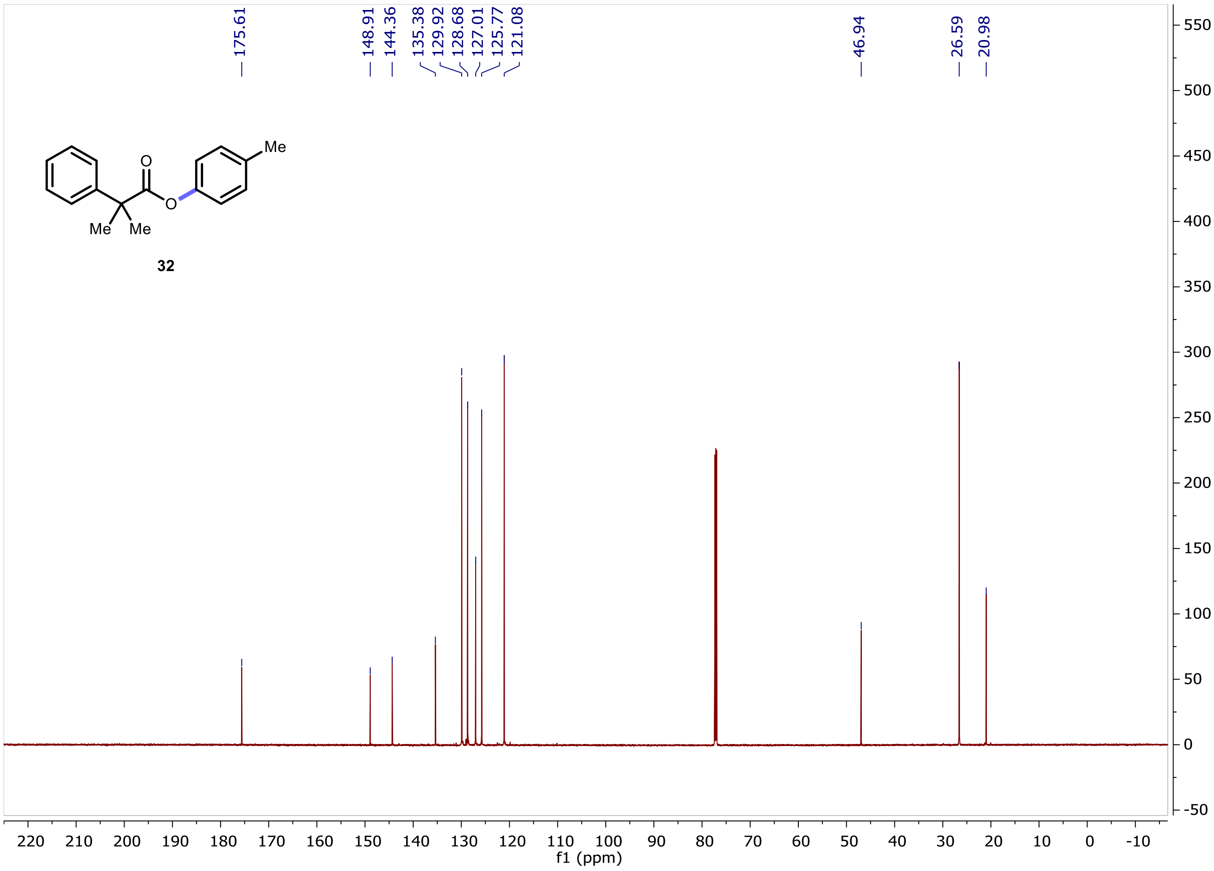

175.61
148.91
144.36
135.38
129.92
128.68
127.01
125.77
121.08
46.94
26.59
20.98
O
O
Me
Me
Me
32
550
500
450
400
350
300
250
200
150
100
50
0
-50
220 210 200 190 180 170 160 150 140 130 120 110 100 90 80 70 60 50 40 30 20 10 0 -10
f1 (ppm)

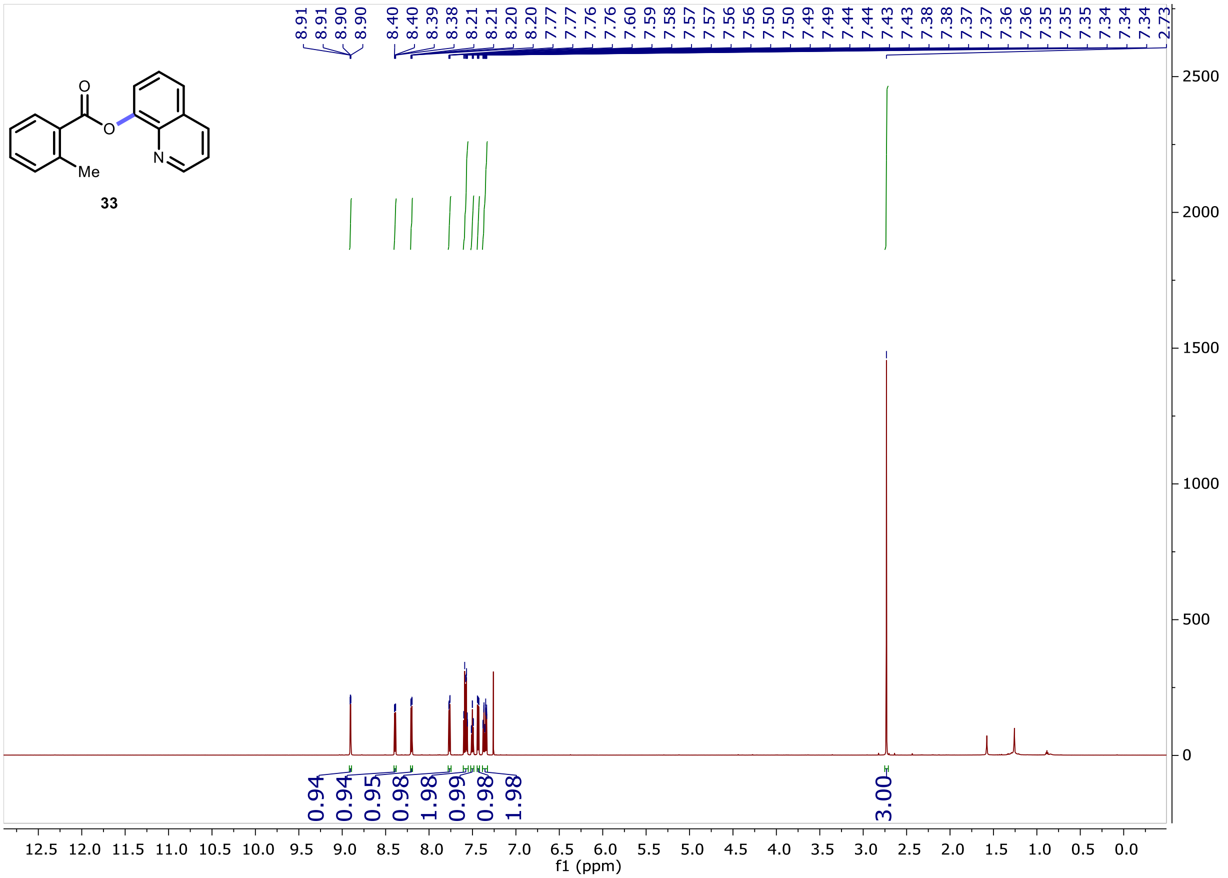
O
O
N
Me
33
8.91
8.91
8.90
8.90
8.40
8.40
8.39
8.38
8.21
8.21
8.20
8.20
7.77
7.77
7.76
7.76
7.60
7.59
7.58
7.57
7.57
7.56
7.56
7.50
7.50
7.49
7.49
7.44
7.44
7.43
7.43
7.38
7.38
7.37
7.37
7.36
7.35
7.35
7.35
7.34
7.34
7.34
2.73
0.94
0.94
0.95
0.98
1.98
0.99
0.99
0.98
1.98
3.00
f1 (ppm)

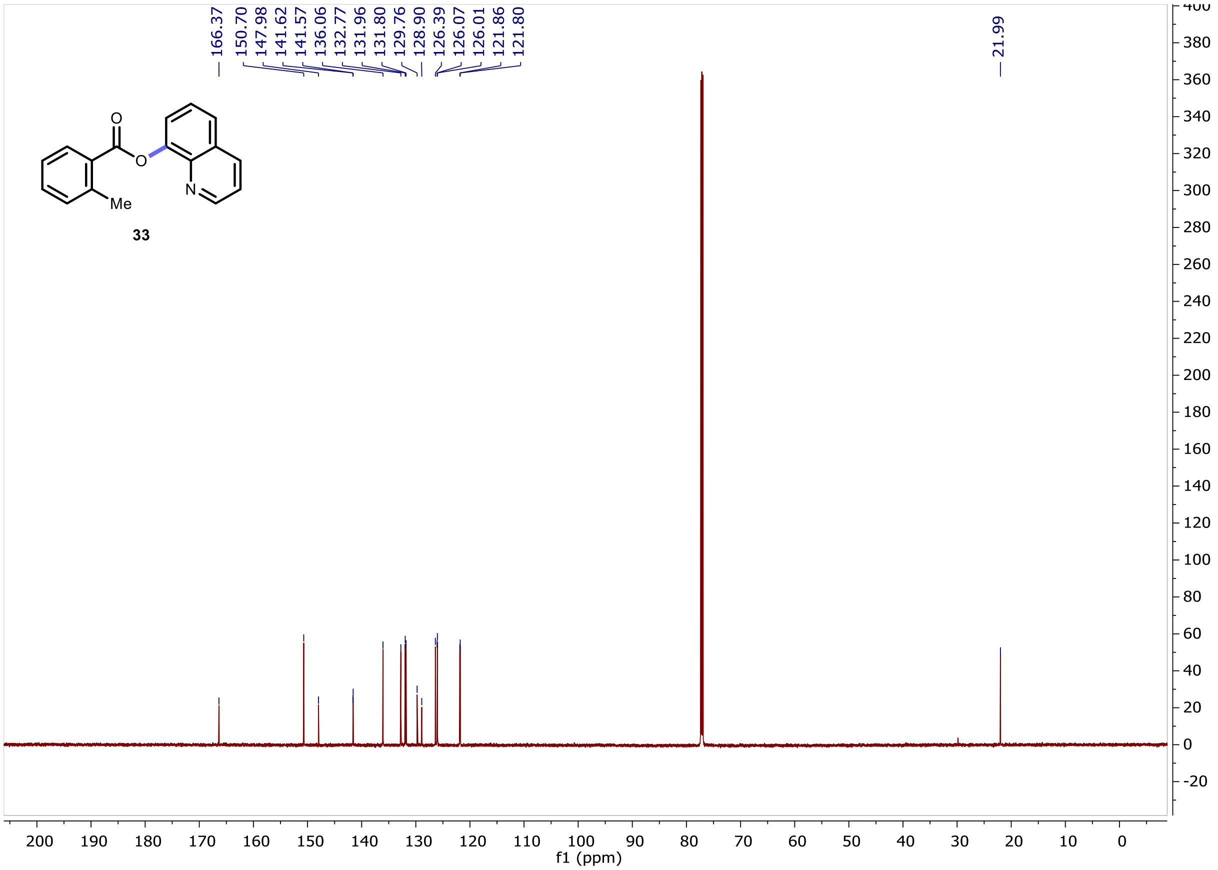
166.37
150.70
147.98
141.62
141.57
136.06
132.77
131.96
131.80
129.76
128.90
126.39
126.07
126.01
121.86
121.80
21.99
O
O
N
Me
33
f1 (ppm)

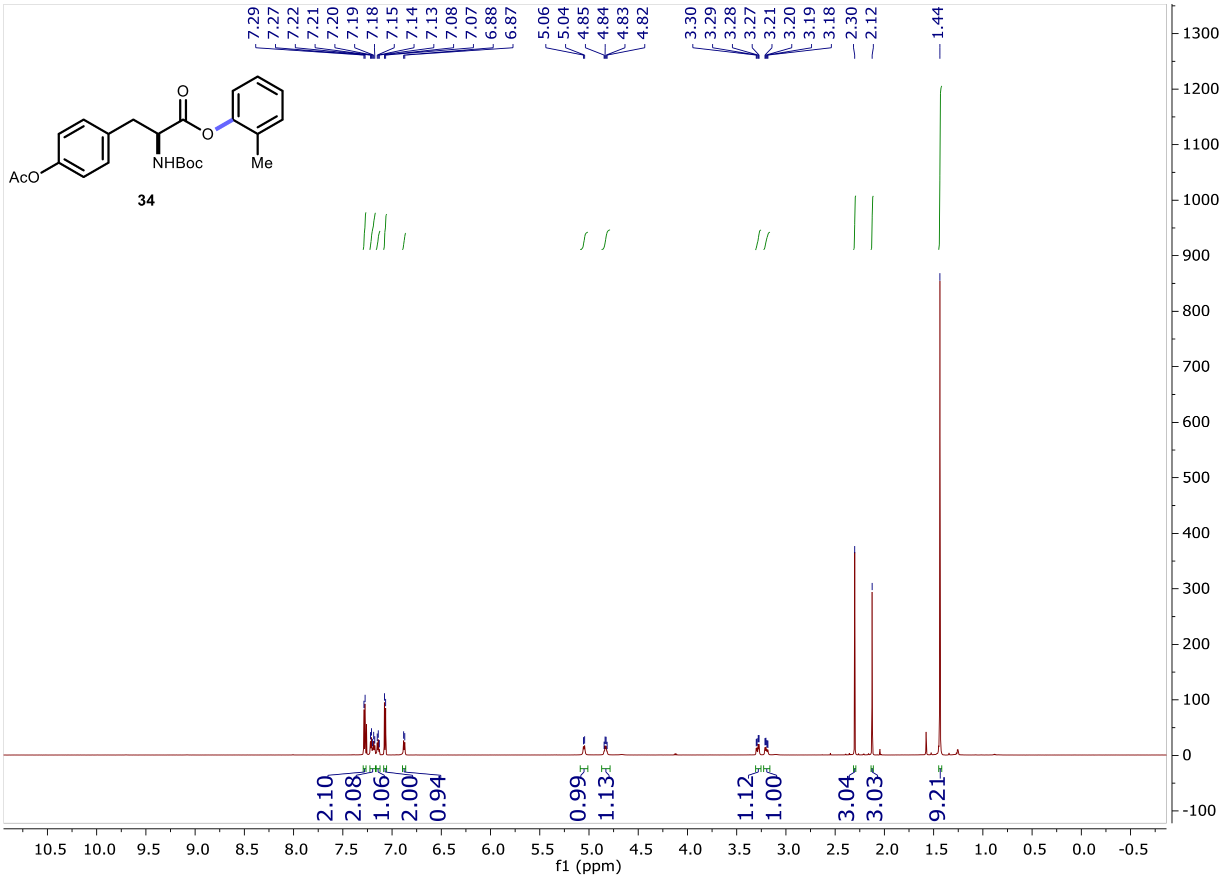

7.29 7.27 7.22 7.21 7.20 7.19 7.18 7.15 7.14 7.13 7.08 7.07 6.88 6.87
5.06 5.04 4.85 4.84 4.83 4.82
3.30 3.29 3.28 3.27 3.21 3.20 3.19 3.18
2.30
2.12
1.44
AcO
O
O
NHBoc
Me
34
2.10 2.08 1.06 2.00 0.94
0.99 1.13
1.12 1.00
3.04
3.03
9.21
f1 (ppm)

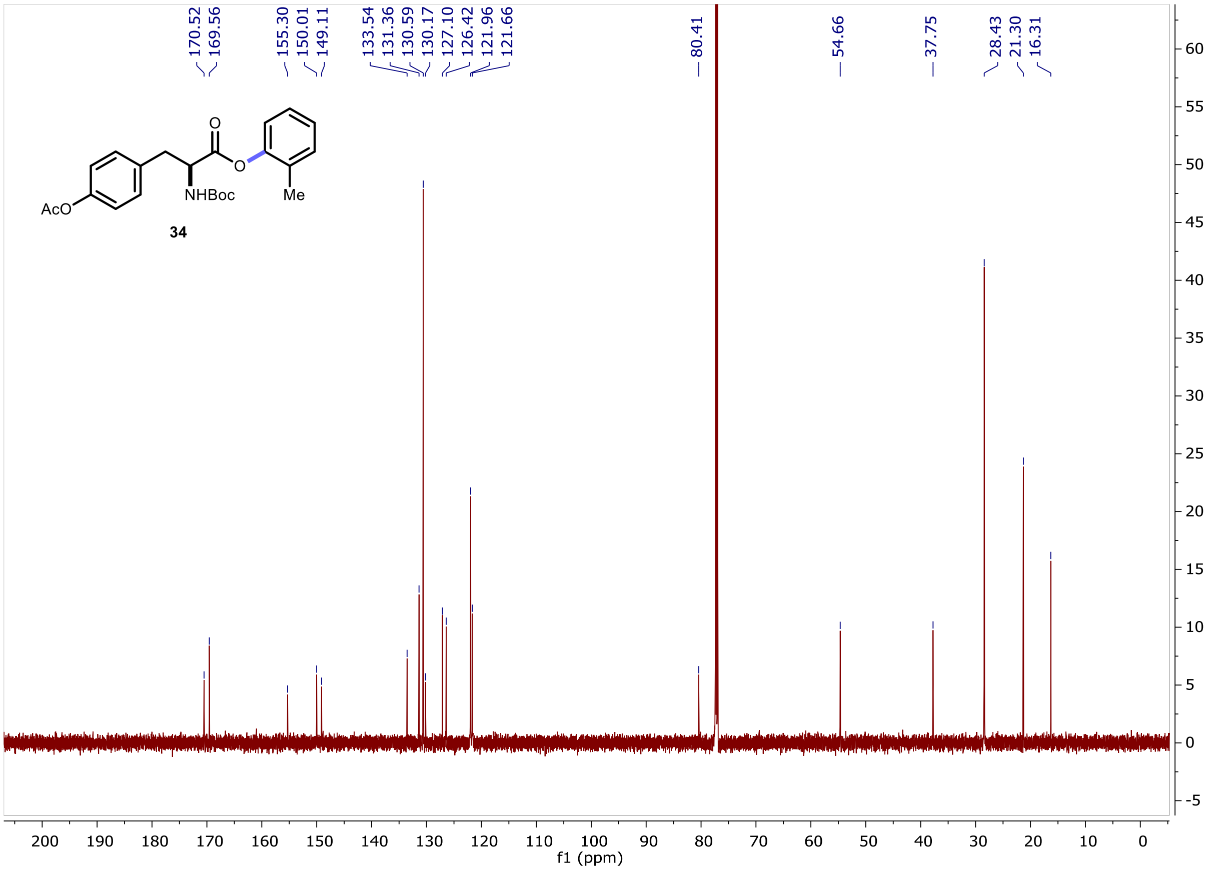
170.52
169.56
155.30
150.01
149.11
133.54
131.36
130.59
130.17
127.10
126.42
121.96
121.66
80.41
54.66
37.75
28.43
21.30
16.31
O
O
AcO
NHBoc
Me
34
f1 (ppm)

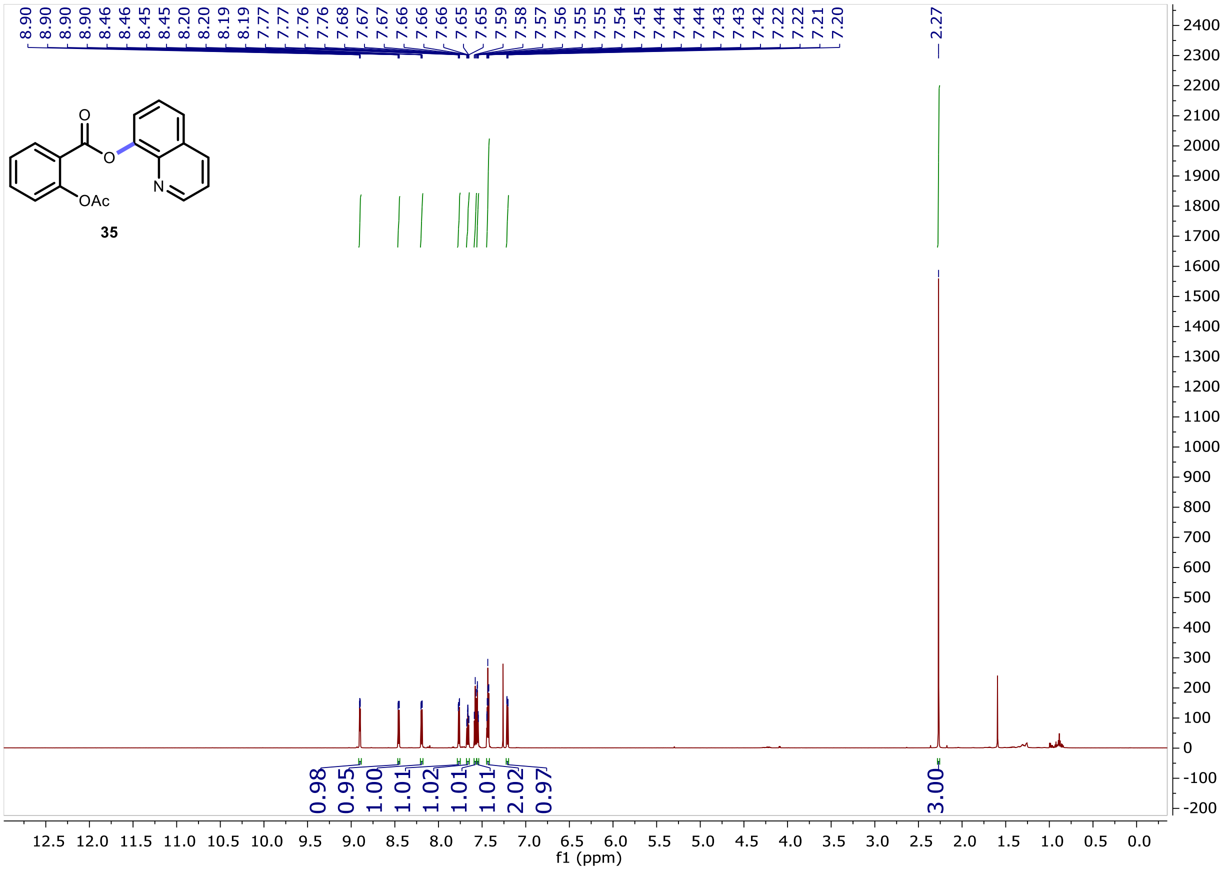

8.90
8.90
8.90
8.90
8.46
8.46
8.45
8.45
8.20
8.20
8.19
8.19
7.77
7.77
7.76
7.76
7.68
7.67
7.67
7.66
7.66
7.65
7.65
7.59
7.58
7.57
7.56
7.55
7.55
7.54
7.45
7.44
7.44
7.43
7.43
7.42
7.22
7.22
7.21
7.20
2.27
O
O
OAc
N
35
0.98
0.95
1.00
1.01
1.02
1.01
1.01
2.02
0.97
3.00
f1 (ppm)

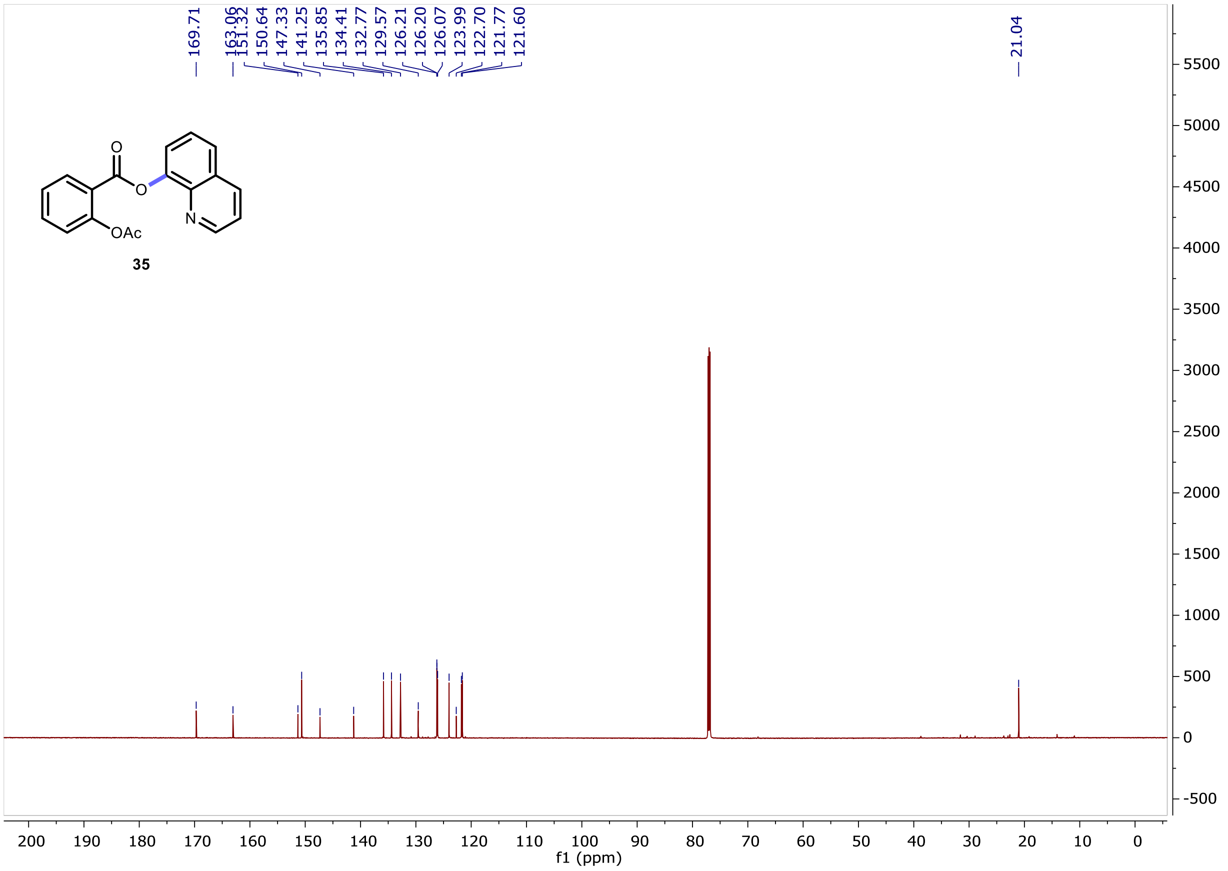
169.71
163.06
151.32
150.64
147.33
141.25
135.85
134.41
132.77
129.57
126.21
126.20
126.07
123.99
122.70
121.77
121.60
21.04
OAc
35
f1 (ppm)

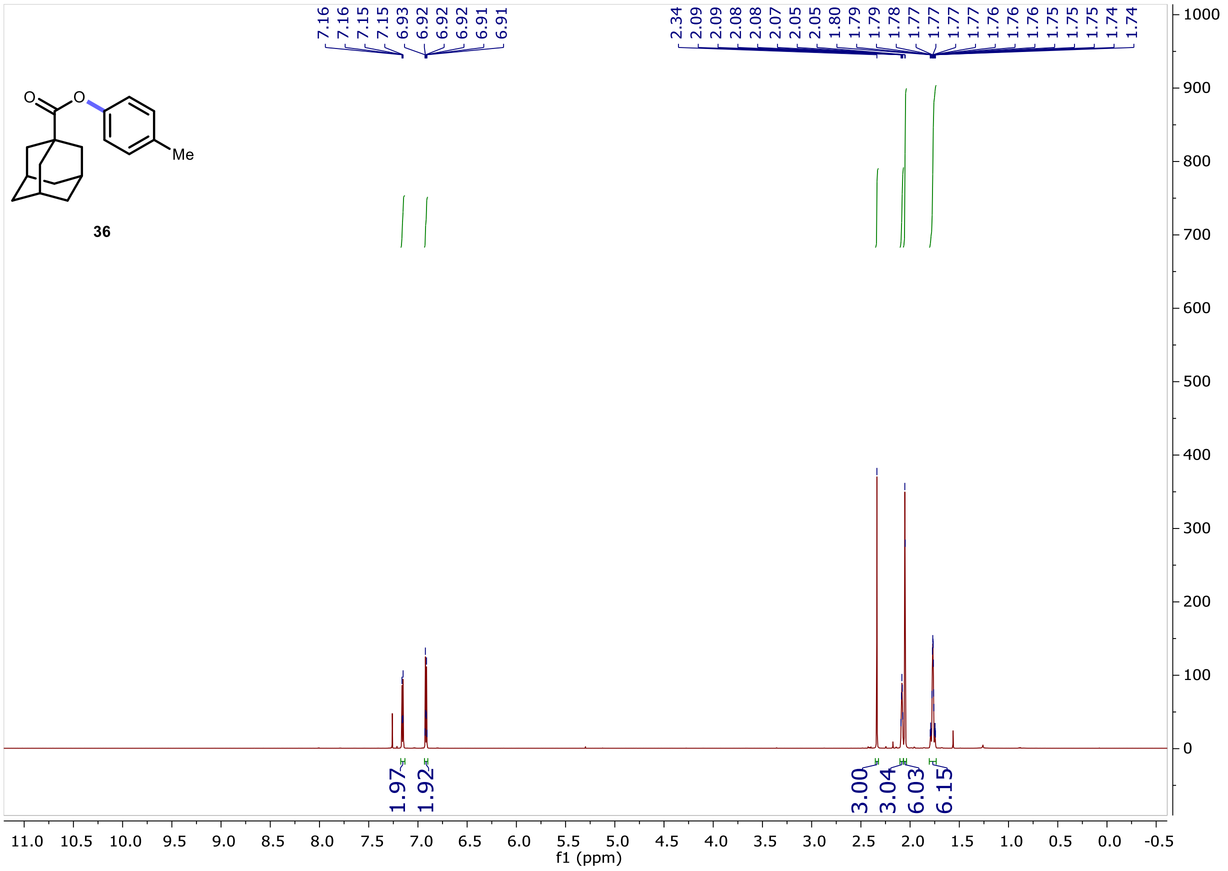
7.16
7.16
7.15
7.15
6.93
6.92
6.92
6.92
6.91
6.91
2.34
2.09
2.09
2.08
2.08
2.07
2.05
2.05
1.80
1.79
1.79
1.78
1.77
1.77
1.77
1.77
1.77
1.76
1.76
1.76
1.75
1.75
1.74
1.74
Me
36
1.97
1.92
3.00
3.04
6.03
6.15
f1 (ppm)

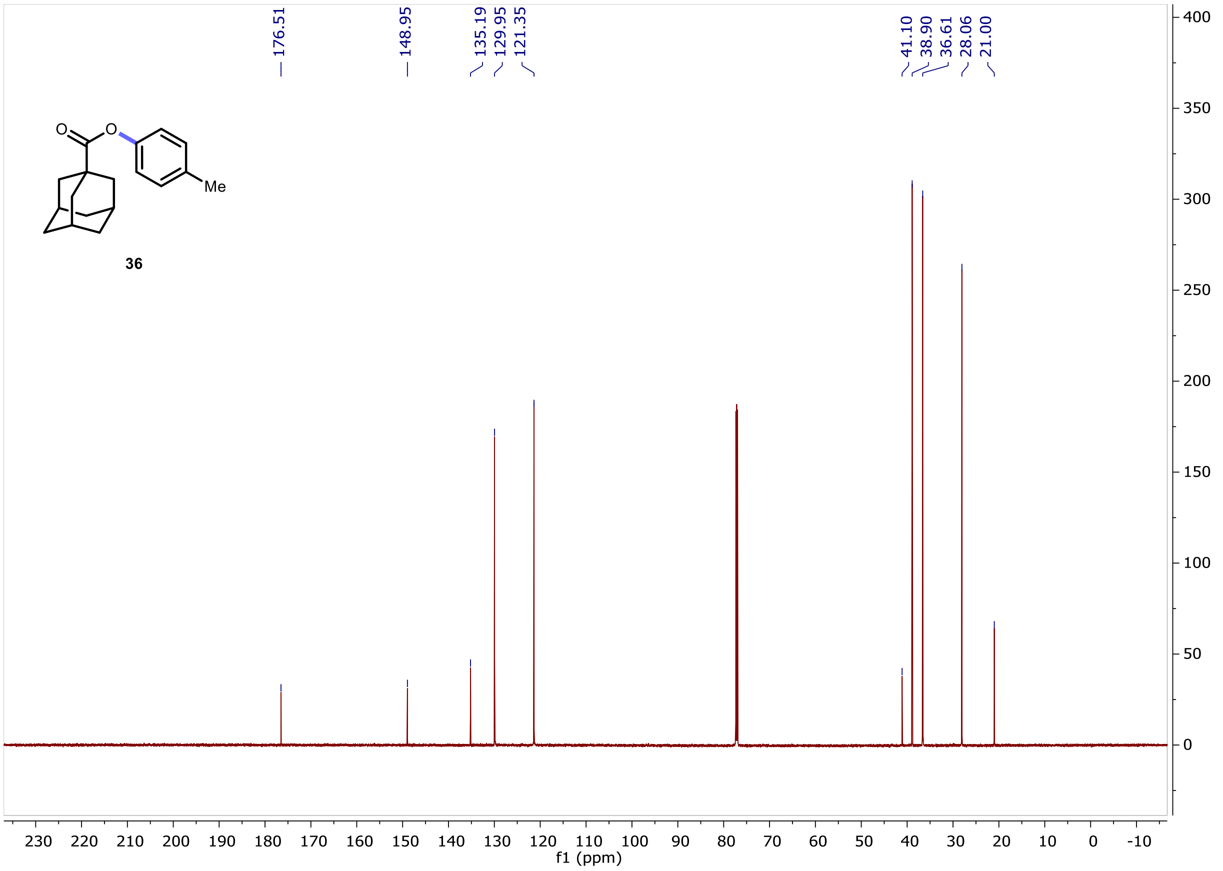
176.51
148.95
135.19
129.95
121.35
41.10
38.90
36.61
28.06
21.00
O
O
Me
36
400
350
300
250
200
150
100
50
0
230 220 210 200 190 180 170 160 150 140 130 120 110 100 90 80 70 60 50 40 30 20 10 0 -10
f1 (ppm)

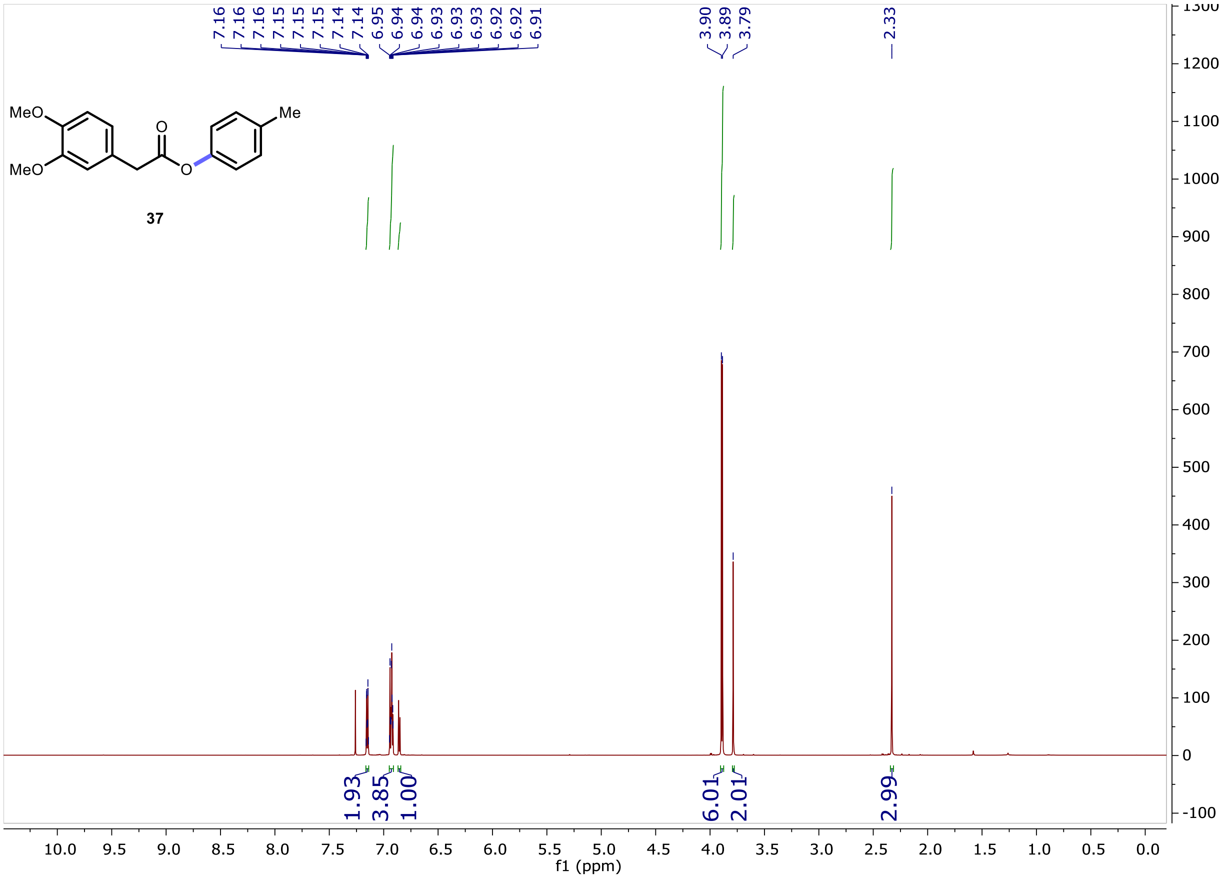
7.16
7.16
7.16
7.15
7.15
7.15
7.14
7.14
6.95
6.94
6.94
6.93
6.93
6.93
6.92
6.92
6.91
3.90
3.89
3.79
2.33
MeO
MeO
O
O
Me
37
1.93
3.85
1.00
6.01
2.01
2.99
f1 (ppm)

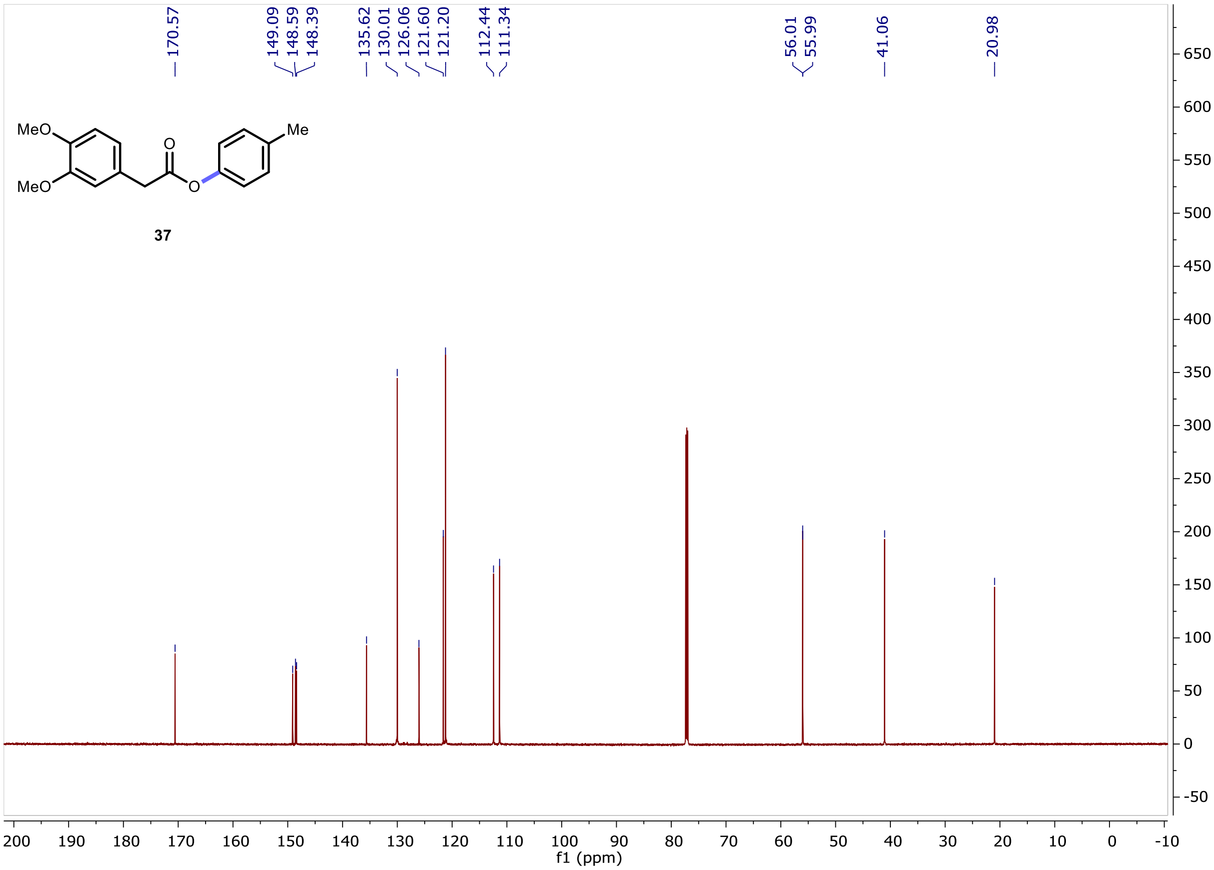
170.57
149.09
148.59
148.39
135.62
130.01
126.06
121.60
121.20
112.44
111.34
56.01
55.99
41.06
20.98
MeO
MeO
O
O
Me
37
f1 (ppm)

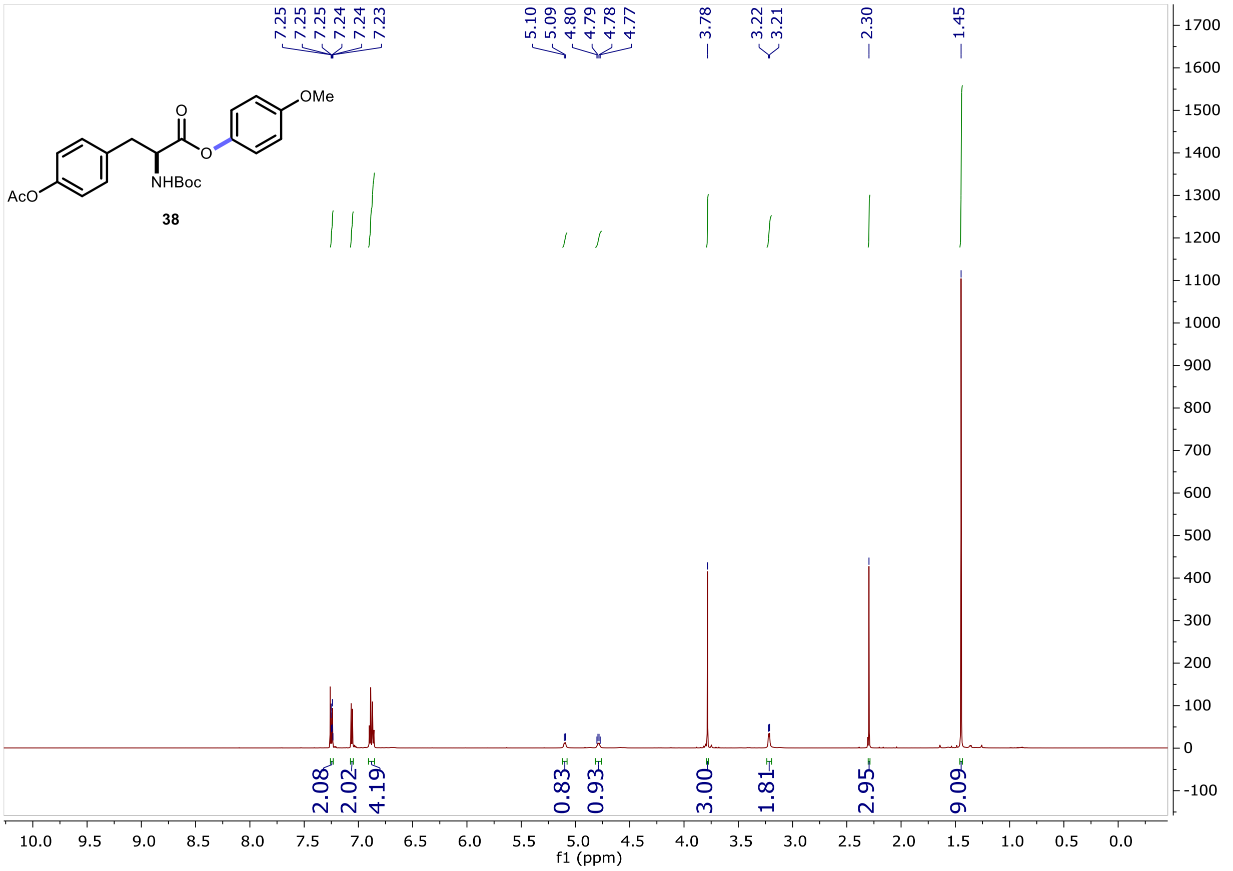

OMe
O
O
AcO
NHBoc
38
7.25
7.25
7.25
7.24
7.24
7.23
5.10
5.09
4.80
4.79
4.78
4.77
3.78
3.22
3.21
2.30
1.45
2.08
2.02
4.19
0.83
0.93
3.00
1.81
2.95
9.09
f1 (ppm)

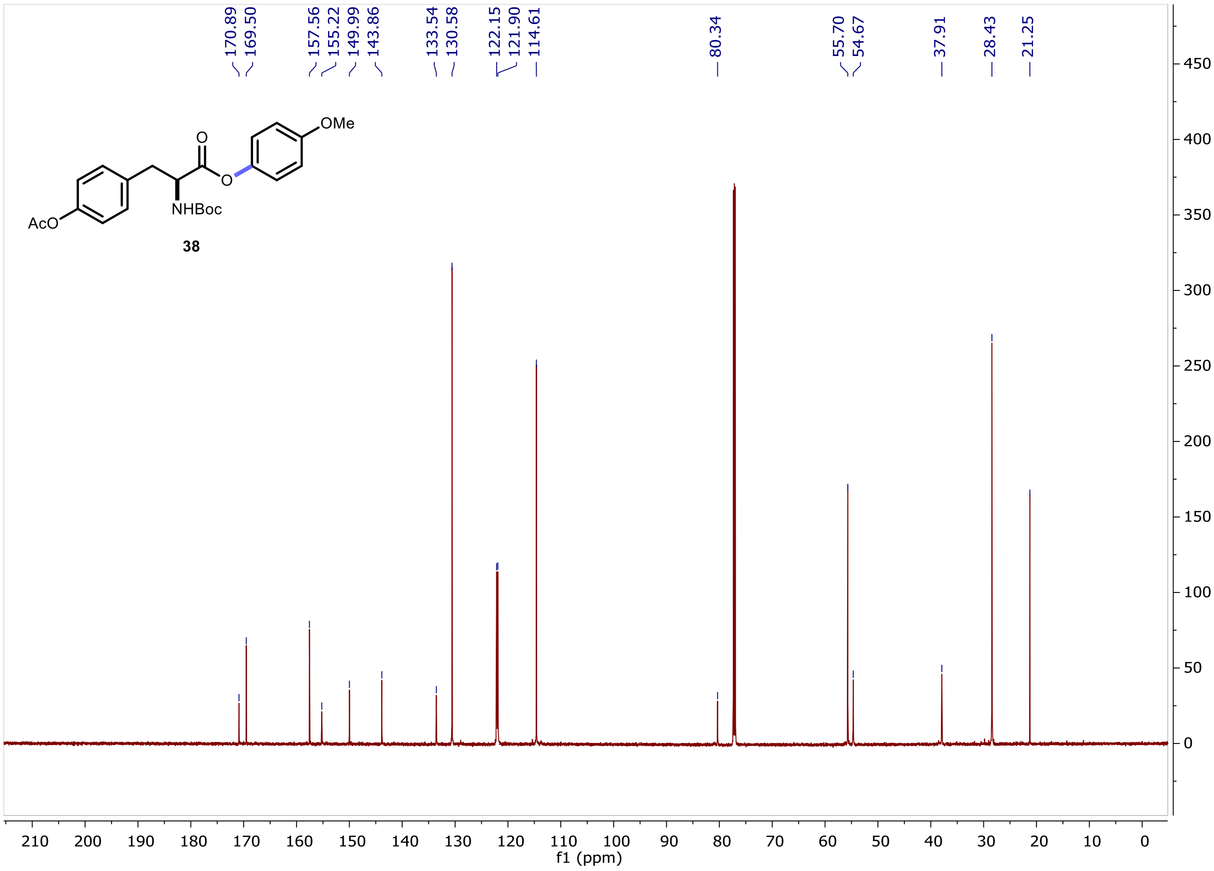
170.89
169.50
157.56
155.22
149.99
143.86
133.54
130.58
122.15
121.90
114.61
80.34
55.70
54.67
37.91
28.43
21.25
OMe
O
O
AcO
NHBoc
38
f1 (ppm)

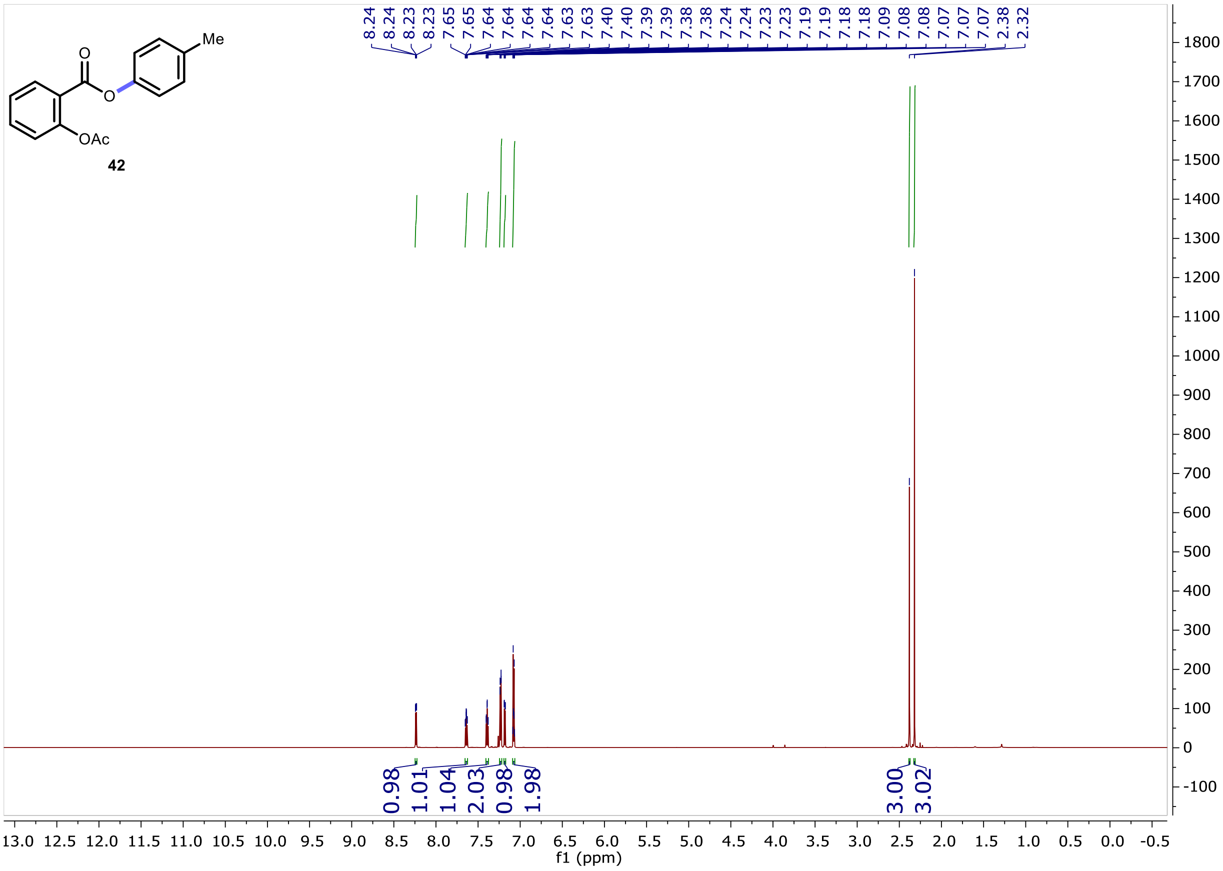
Me
O
O
OAc
42
8.24
8.24
8.23
8.23
7.65
7.65
7.64
7.64
7.64
7.64
7.63
7.63
7.40
7.40
7.39
7.39
7.38
7.38
7.24
7.24
7.23
7.23
7.19
7.19
7.18
7.18
7.09
7.08
7.08
7.07
7.07
7.07
2.38
2.32
0.98
1.01
1.04
2.03
0.98
1.98
3.00
3.02
f1 (ppm)

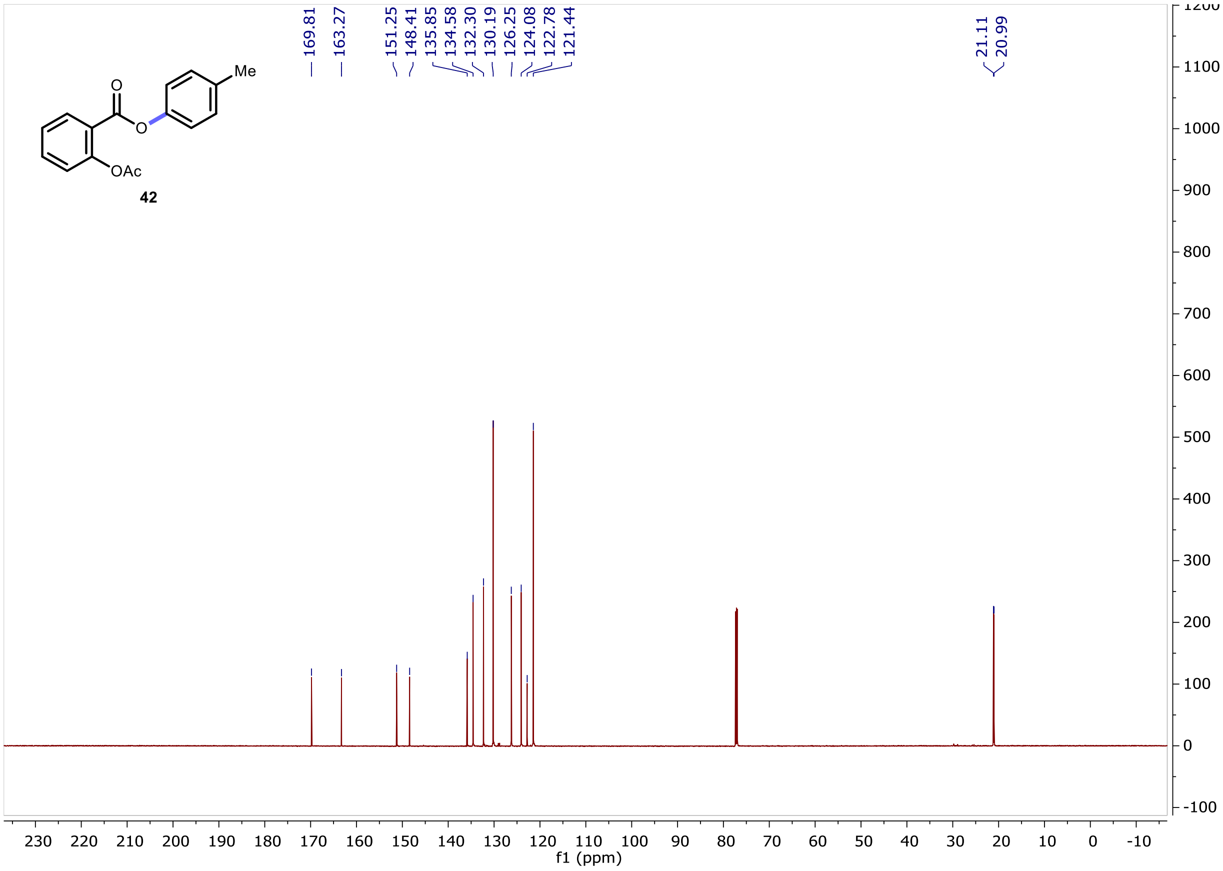

169.81
163.27
151.25
148.41
135.85
134.58
132.30
130.19
126.25
124.08
122.78
121.44
21.11
20.99
Me
O
O
OAc
42
f1 (ppm)

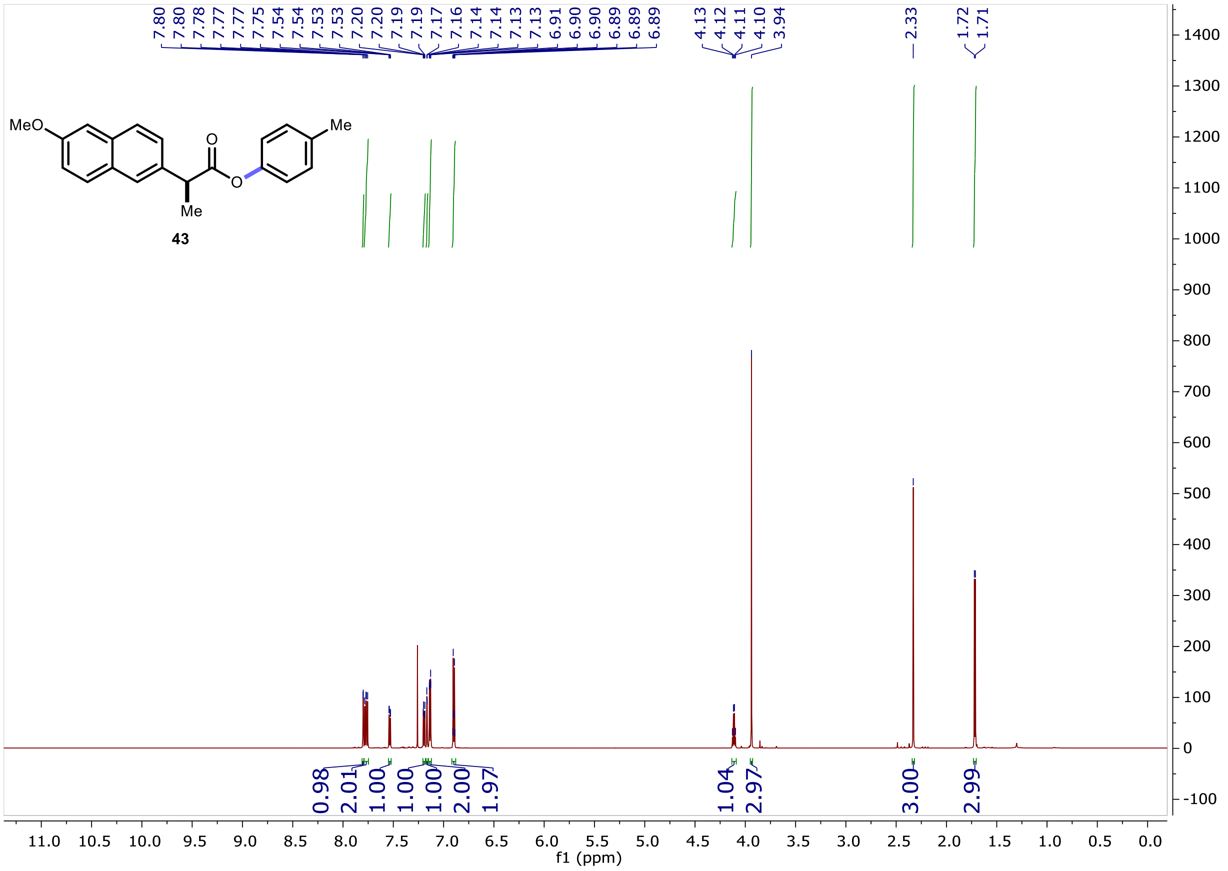
7.80
7.80
7.78
7.77
7.77
7.75
7.54
7.54
7.53
7.53
7.20
7.20
7.19
7.19
7.17
7.16
7.14
7.14
7.13
7.13
6.91
6.90
6.90
6.89
6.89
6.89
4.13
4.12
4.11
4.10
3.94
2.33
1.72
1.71
MeO
O
O
Me
Me
43
0.98
2.01
1.00
1.00
1.00
2.00
1.97
1.04
2.97
3.00
2.99
f1 (ppm)

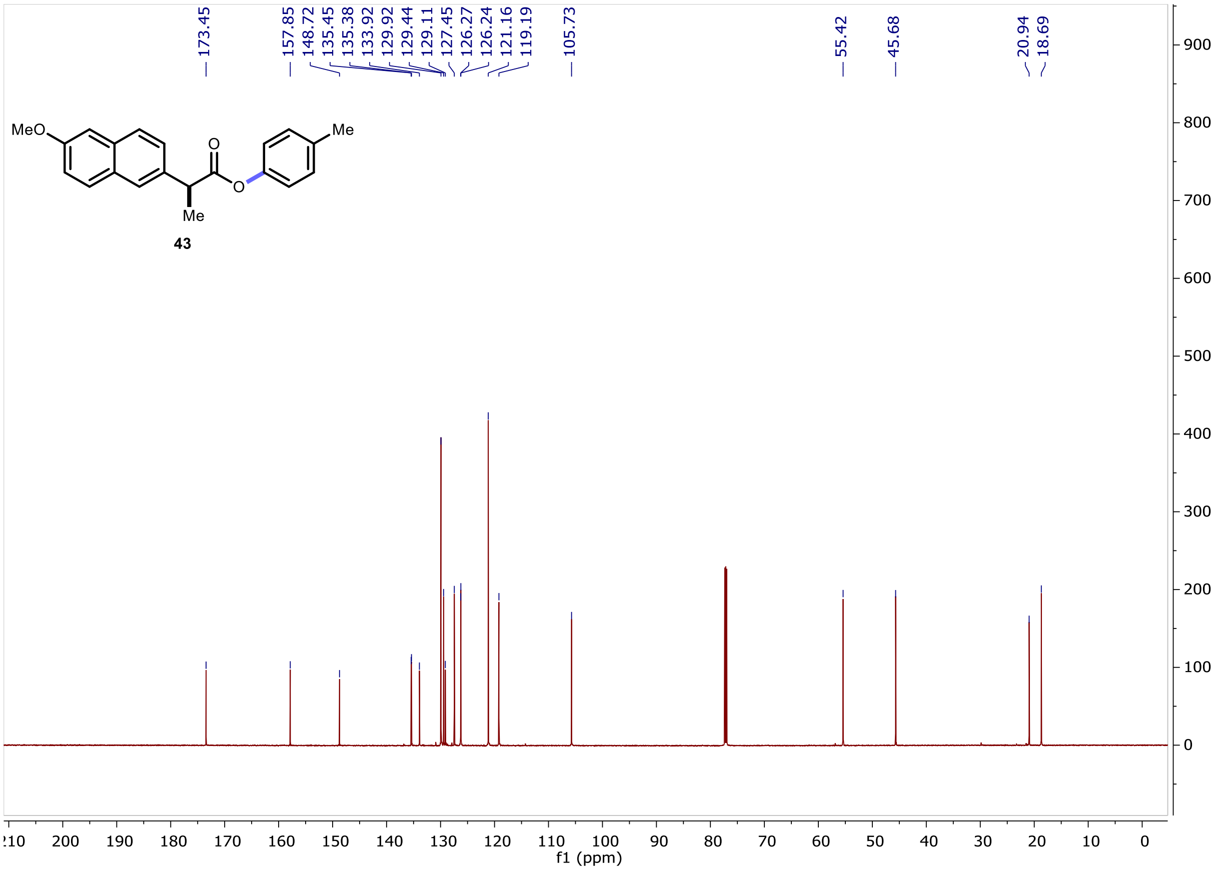
173.45
157.85
148.72
135.45
135.38
133.92
129.92
129.44
129.11
127.45
126.27
126.24
121.16
119.19
105.73
55.42
45.68
20.94
18.69
MeO
O
O
Me
Me
43
f1 (ppm)

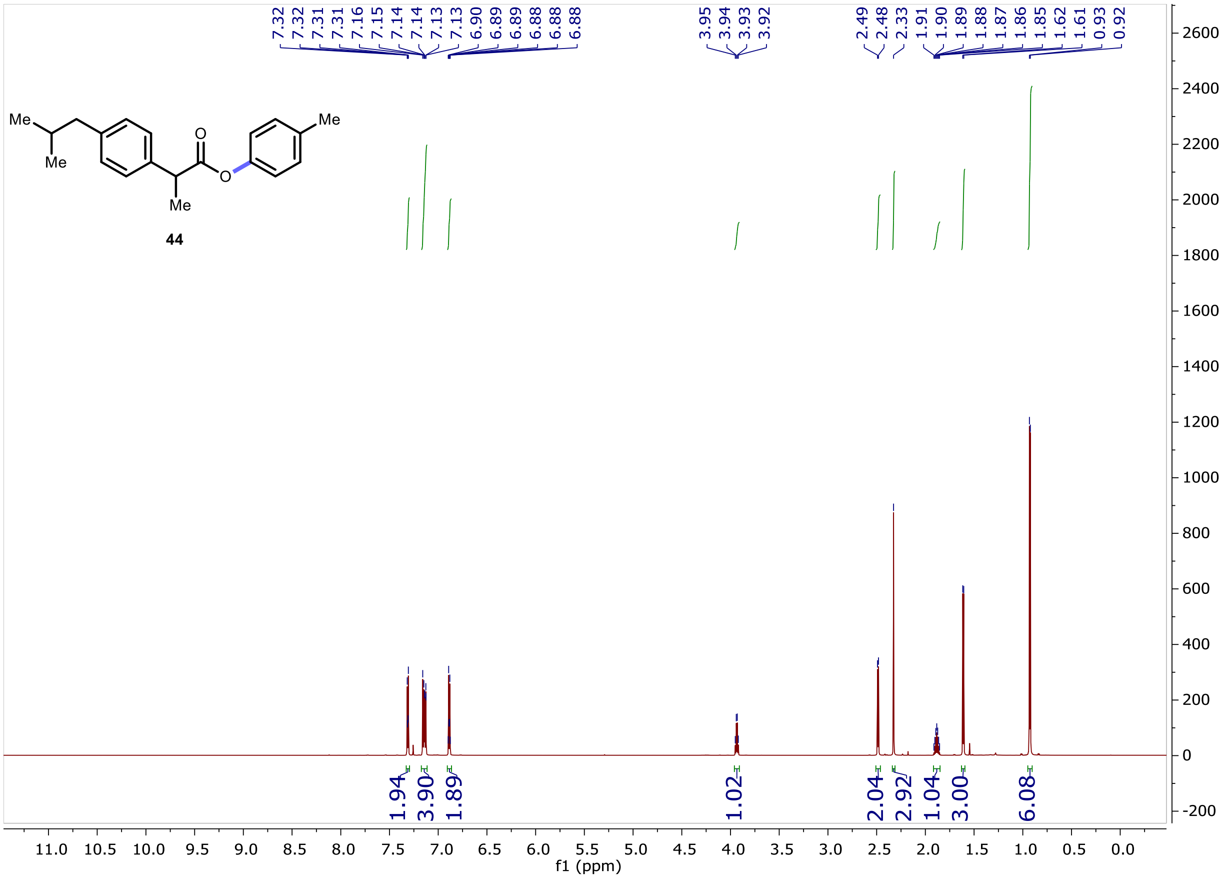

Me
Me
O
O
Me
Me
44
7.32
7.32
7.31
7.31
7.16
7.15
7.14
7.14
7.13
7.13
6.90
6.89
6.89
6.88
6.88
6.88
3.95
3.94
3.93
3.92
2.49
2.48
2.33
1.91
1.90
1.89
1.88
1.87
1.86
1.85
1.62
1.61
0.93
0.92
1.94
3.90
1.89
1.02
2.04
2.92
1.04
3.00
6.08
f1 (ppm)

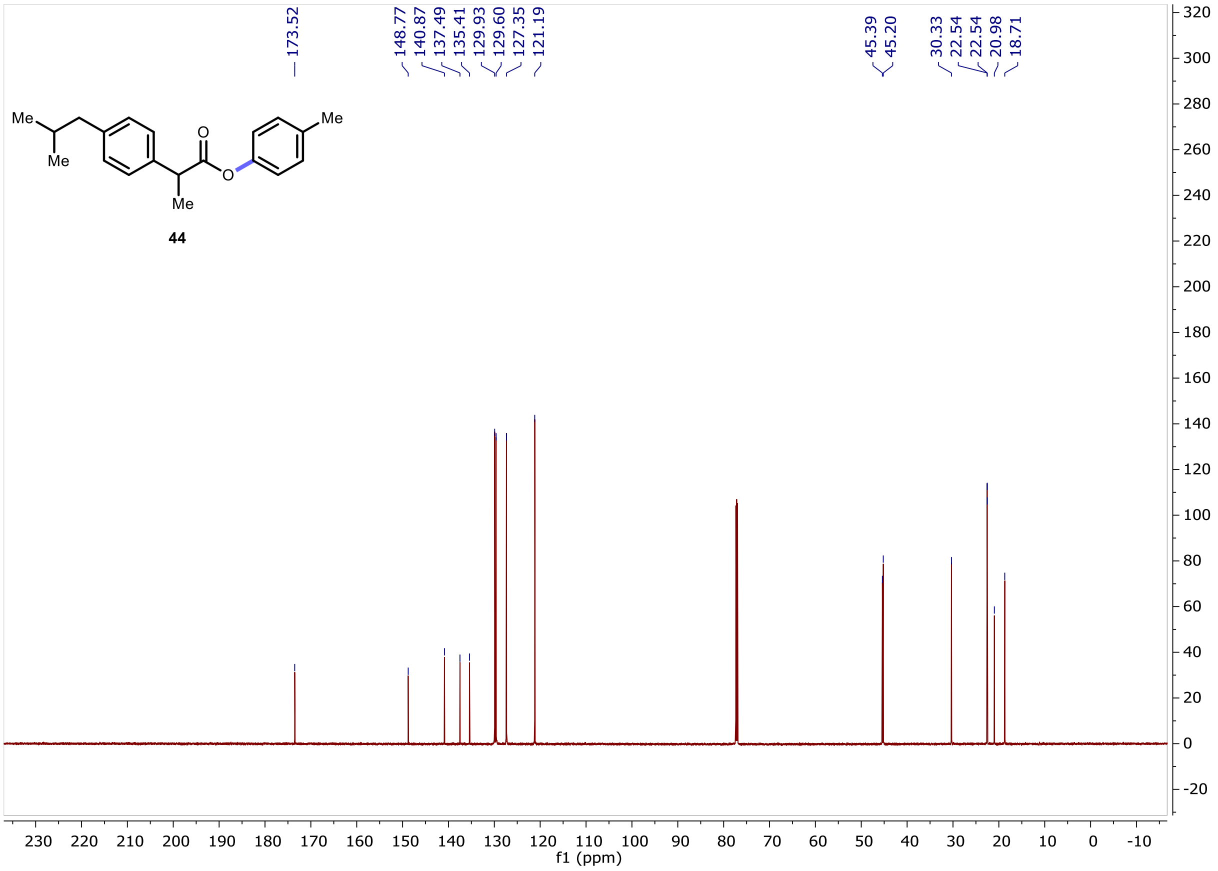
173.52
148.77
140.87
137.49
135.41
129.93
129.60
127.35
121.19
45.39
45.20
30.33
22.54
22.54
20.98
18.71
Me
Me
O
O
Me
Me
44
f1 (ppm)

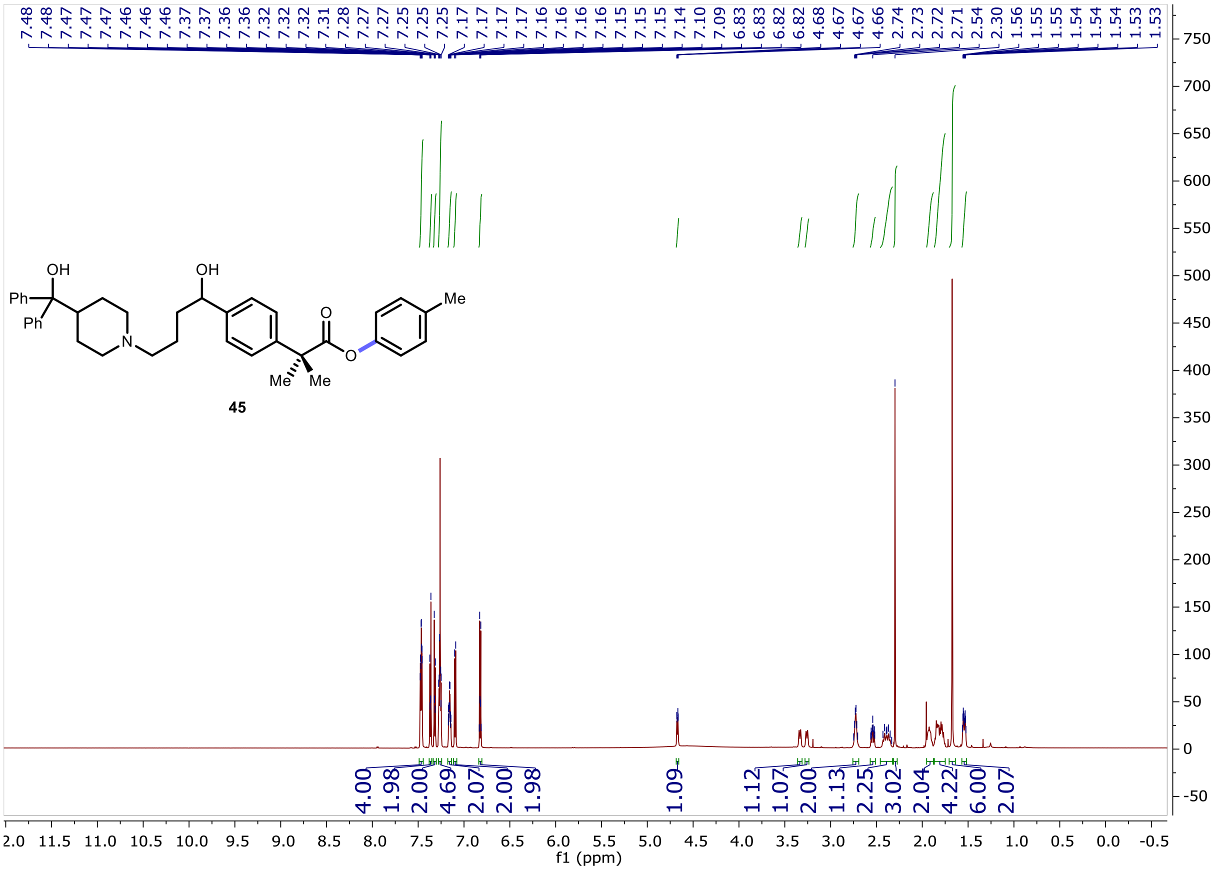

45

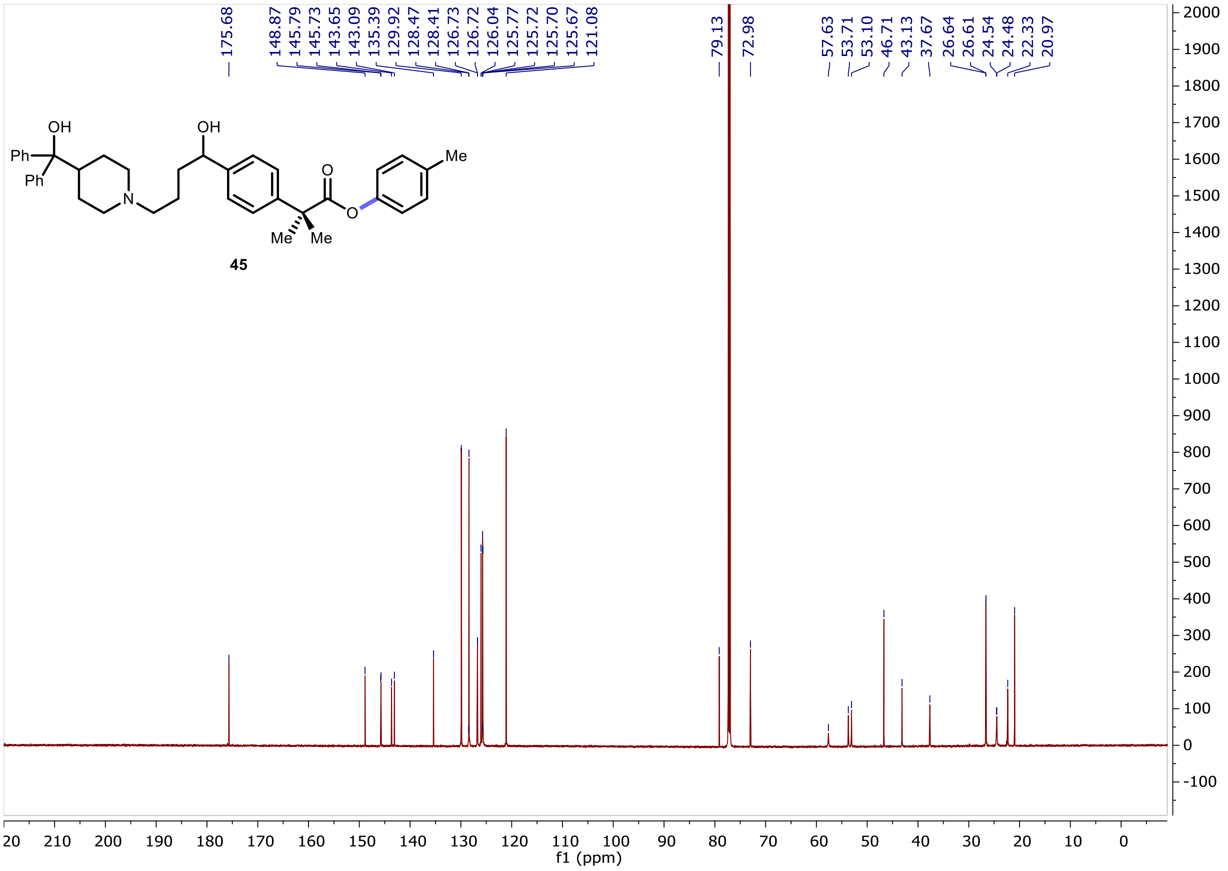

175.68
148.87
145.79
145.73
143.65
143.09
135.39
129.92
128.47
128.41
126.73
126.72
126.04
125.77
125.72
125.70
125.67
121.08
79.13
72.98
57.63
53.71
53.10
46.71
43.13
37.67
26.64
26.61
24.54
24.48
22.33
20.97
OH
OH
Ph
Ph
N
O
O
Me
Me
Me
45
f1 (ppm)

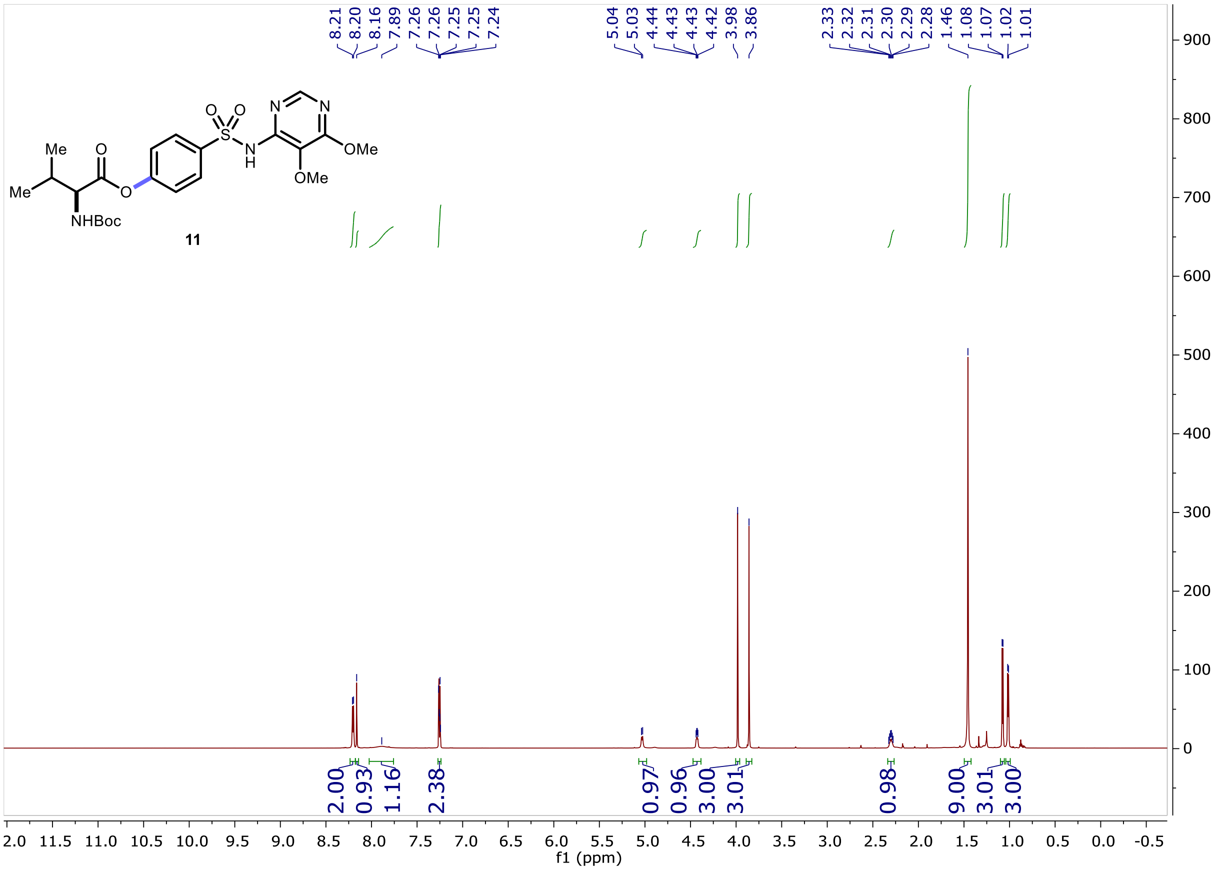

8.21
8.20
8.16
7.89
7.26
7.26
7.25
7.25
7.24
5.04
5.03
4.44
4.43
4.43
4.42
3.98
3.86
2.33
2.32
2.31
2.30
2.29
2.28
1.46
1.08
1.07
1.02
1.01
11
2.00
0.93
1.16
2.38
0.97
0.96
3.00
3.01
0.98
9.00
3.01
3.00
f1 (ppm)

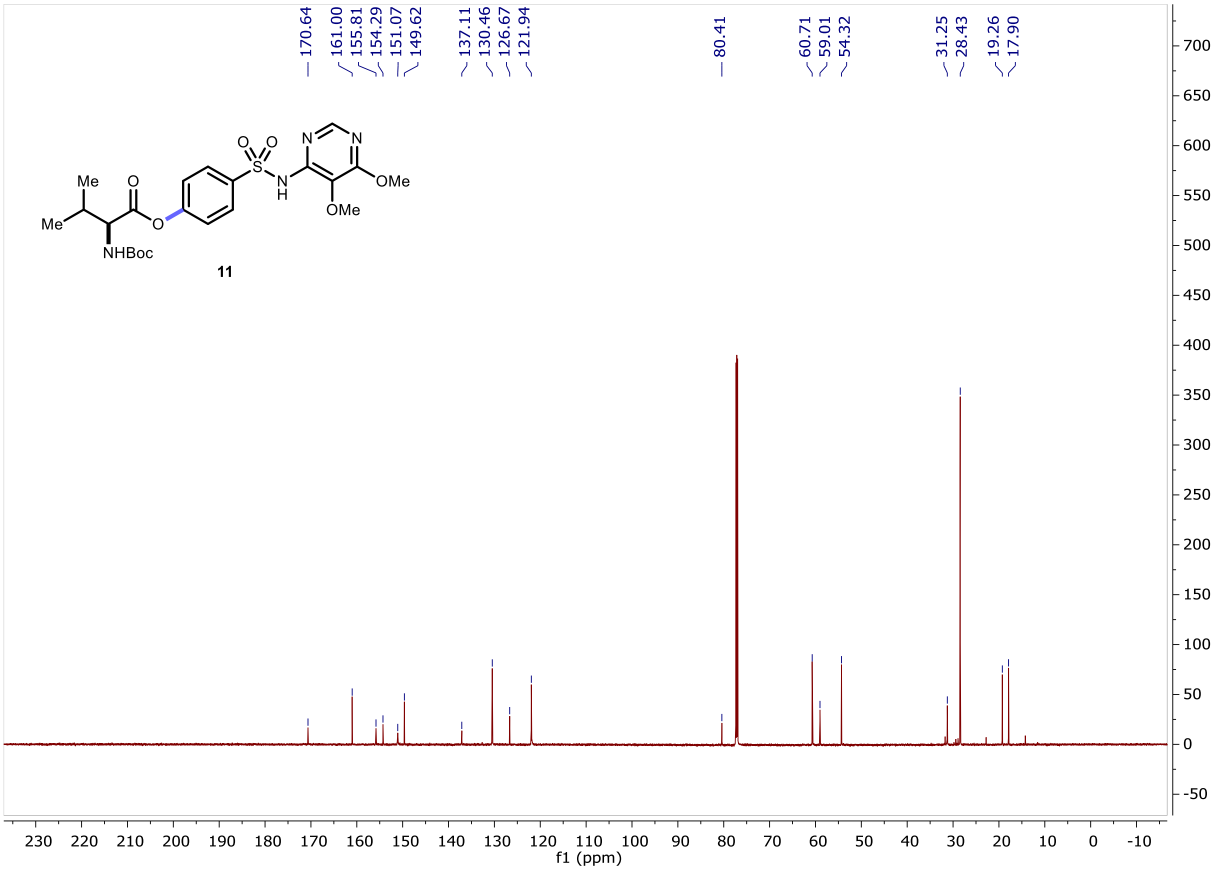

170.64
161.00
155.81
154.29
151.07
149.62
137.11
130.46
126.67
121.94
80.41
60.71
59.01
54.32
31.25
28.43
19.26
17.90
Me
O
Me
O
NHBoc
O
O
S
N
H
N
N
OMe
OMe
11
f1 (ppm)

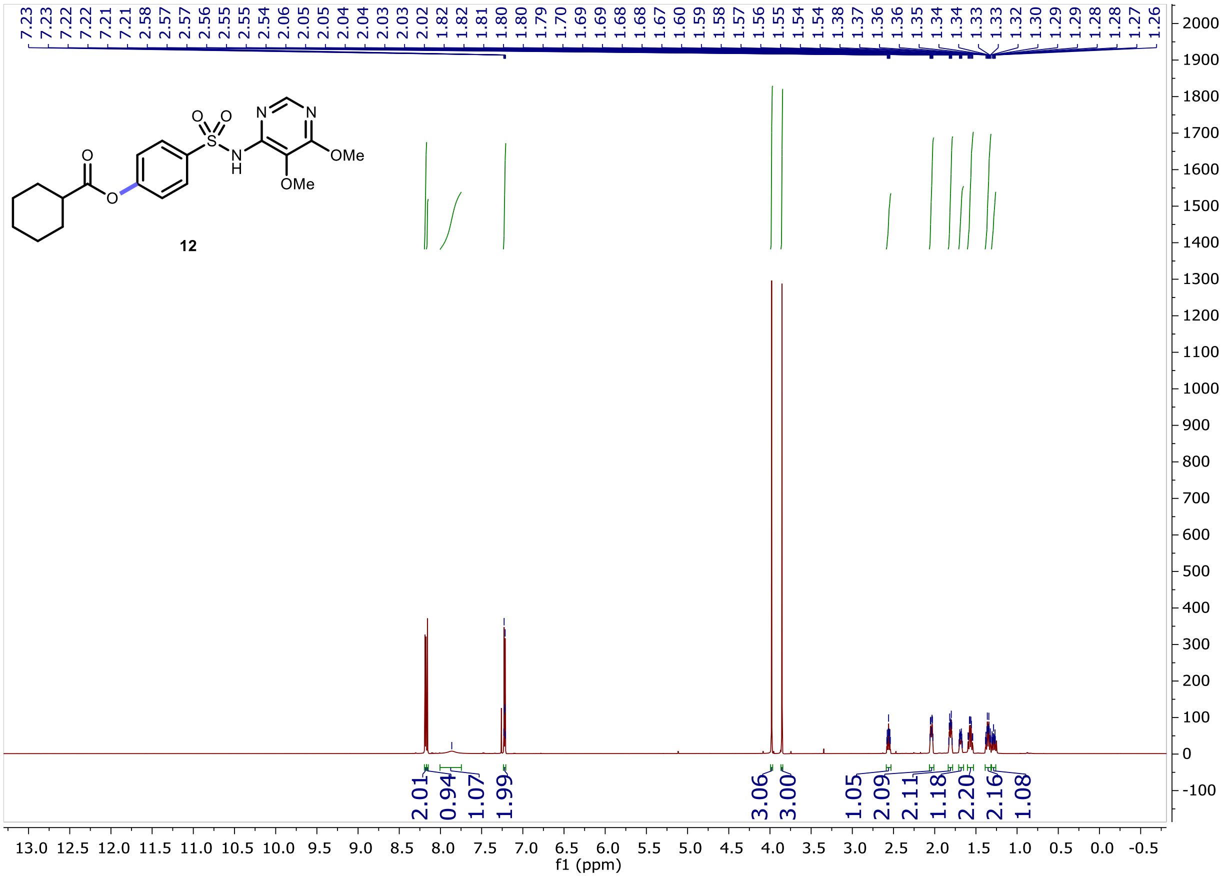

7.23
7.23
7.22
7.22
7.21
7.21
2.58
2.57
2.57
2.56
2.55
2.55
2.54
2.06
2.05
2.05
2.04
2.04
2.03
2.03
2.02
1.82
1.82
1.81
1.80
1.80
1.79
1.70
1.69
1.69
1.68
1.68
1.67
1.60
1.59
1.58
1.57
1.56
1.55
1.54
1.54
1.38
1.37
1.36
1.36
1.35
1.34
1.34
1.33
1.33
1.32
1.30
1.29
1.29
1.28
1.28
1.27
1.26
OMe
OMe
12
2.01
0.94
1.07
1.99
3.06
3.00
1.05
2.09
2.11
1.18
2.20
2.16
1.08
f1 (ppm)

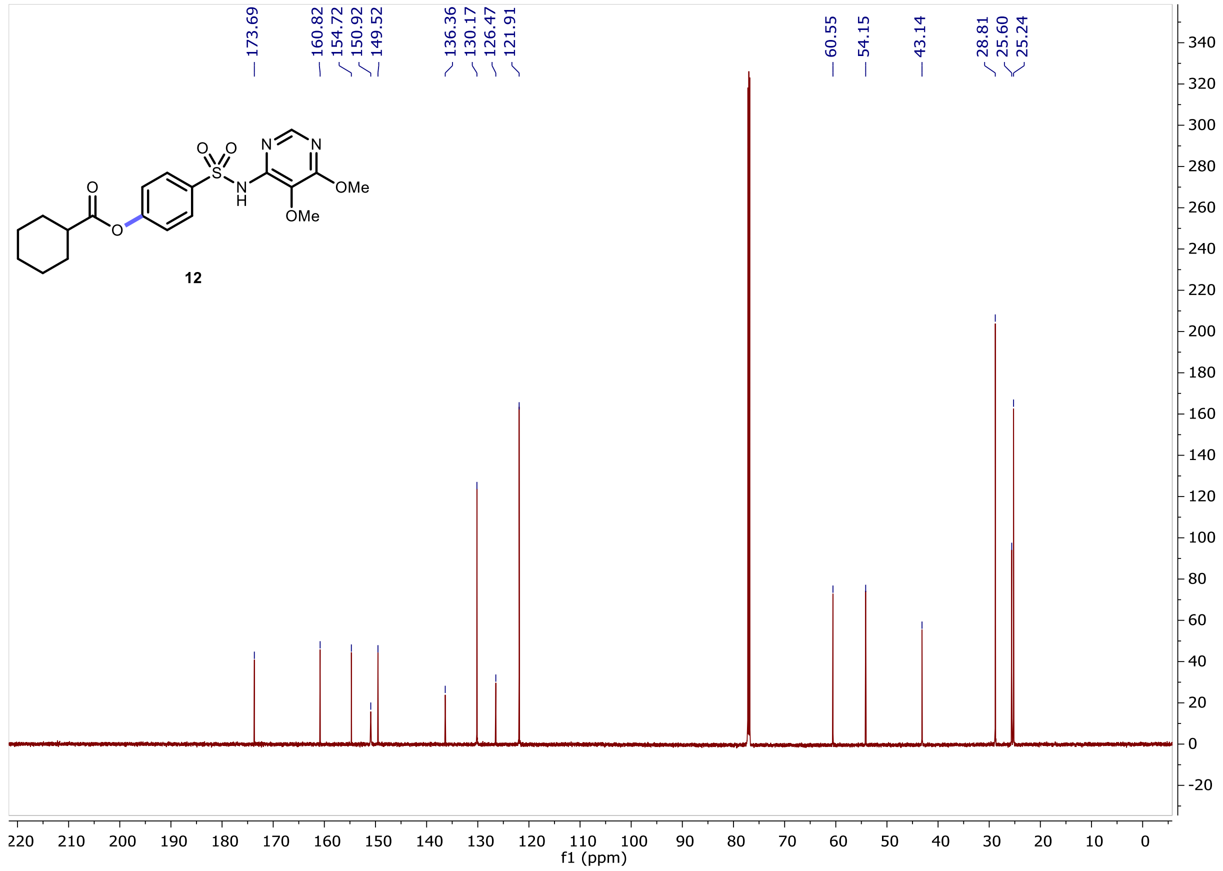
173.69
160.82
154.72
150.92
149.52
136.36
130.17
126.47
121.91
60.55
54.15
43.14
28.81
25.60
25.24
O
O
O
S
O
N
H
N
N
OMe
OMe
12
f1 (ppm)

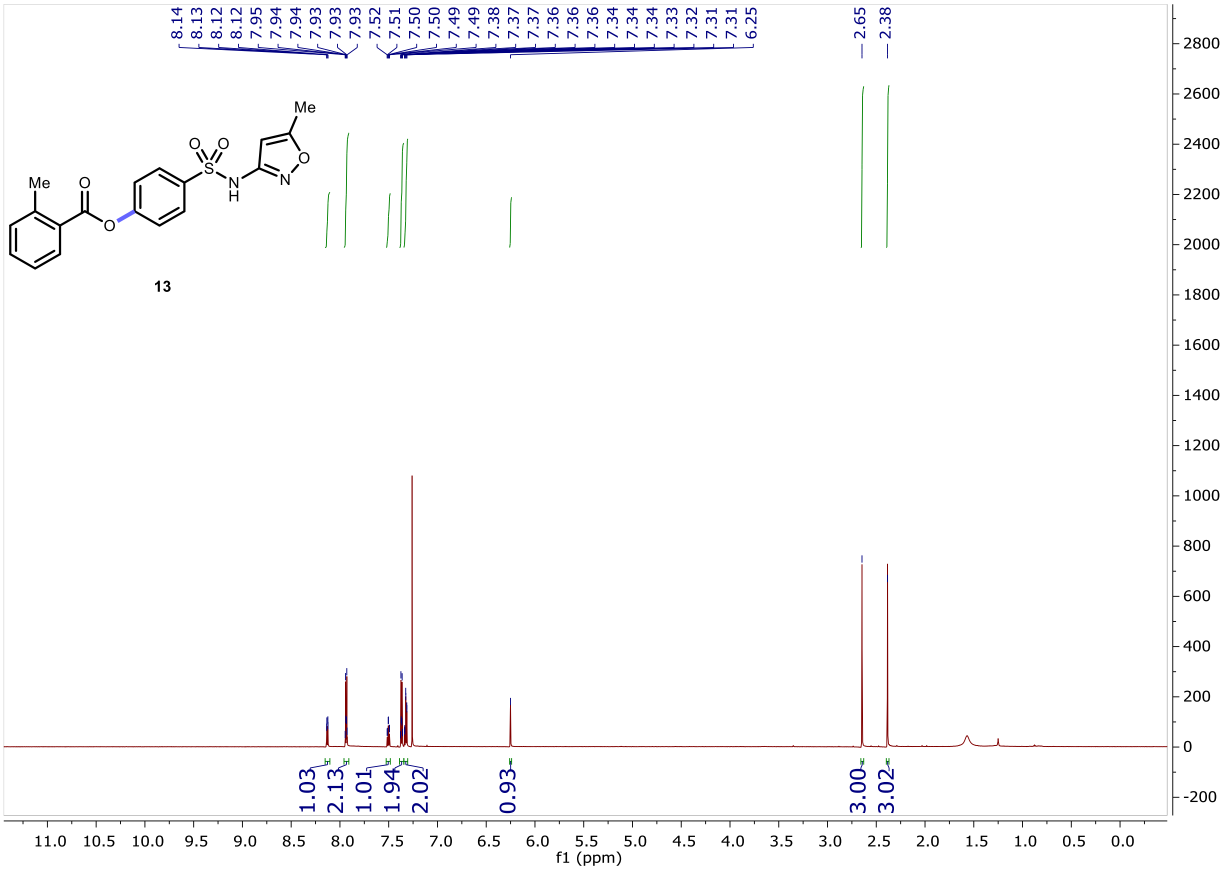
8.14
8.13
8.12
8.12
7.95
7.94
7.94
7.93
7.93
7.93
7.52
7.51
7.50
7.50
7.49
7.49
7.38
7.37
7.37
7.36
7.36
7.34
7.34
7.34
7.33
7.32
7.31
7.31
6.25
2.65
2.38
Me
O
O
O
S
N
H
Me
O
N
O
13
1.03
2.13
1.01
1.94
2.02
0.93
3.00
3.02
f1 (ppm)

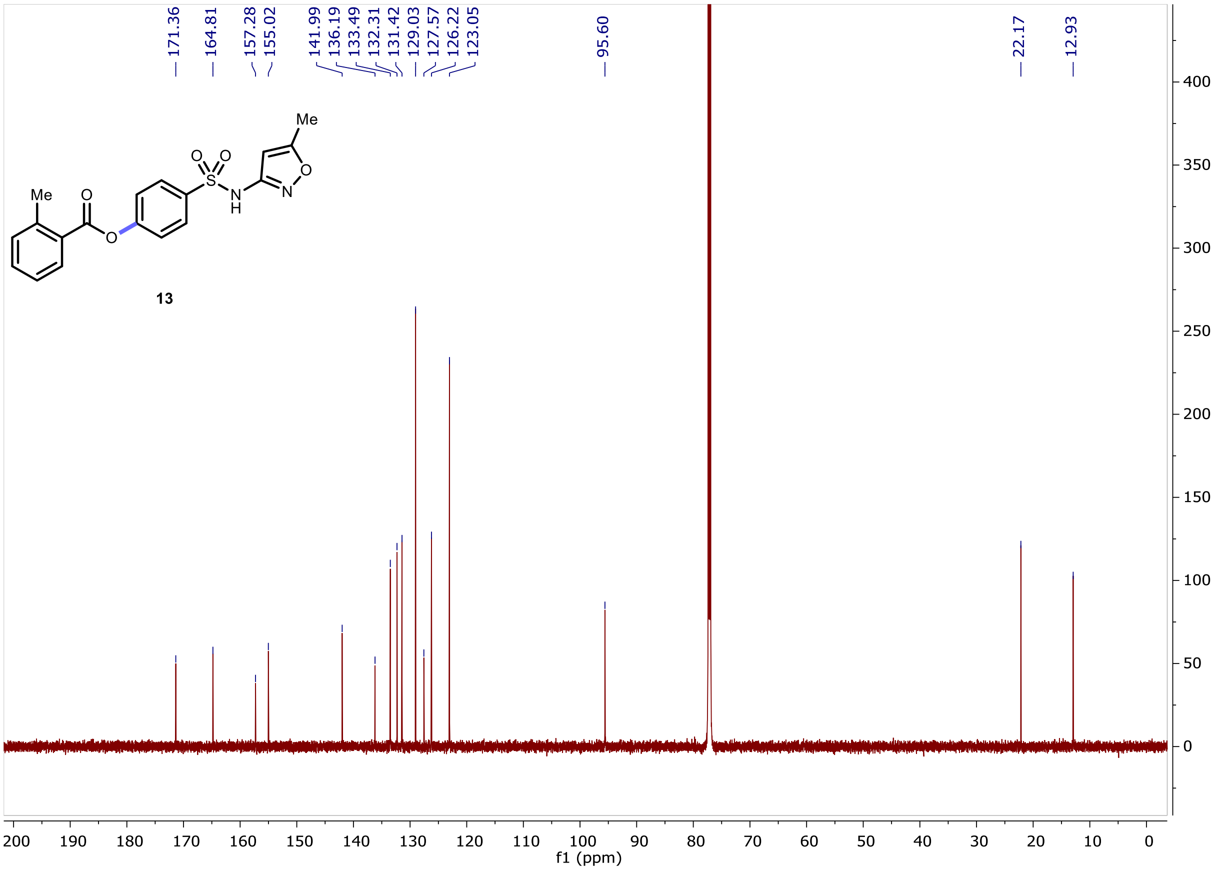

171.36
164.81
157.28
155.02
141.99
136.19
133.49
132.31
131.42
129.03
127.57
126.22
123.05
95.60
22.17
12.93
Me
O
O
S
N
H
Me
O
N
O
O
13
400
350
300
250
200
150
100
50
0
200
190
180
170
160
150
140
130
120
110
100
90
80
70
60
50
40
30
20
10
0
f1 (ppm)

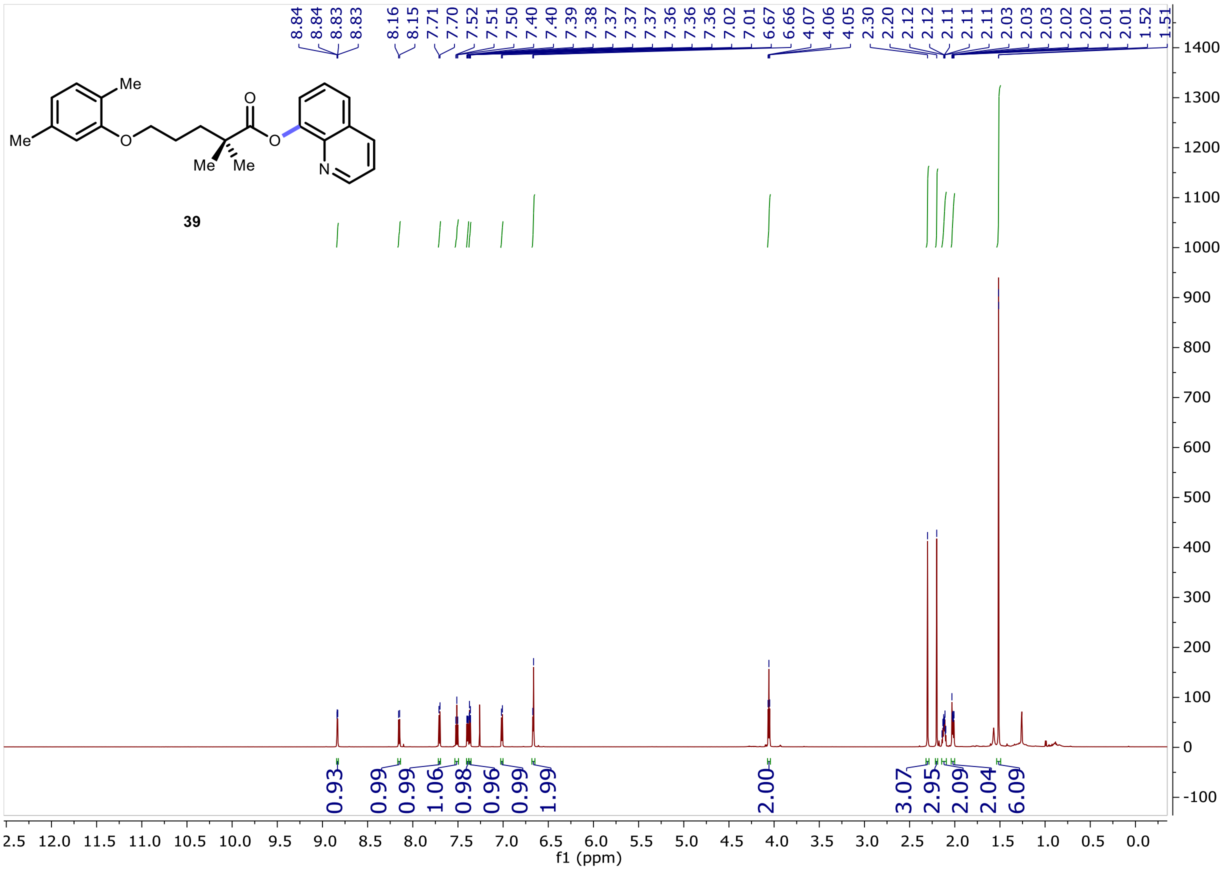
Me
Me
O
O
O
Me
Me
N
39
f1 (ppm)

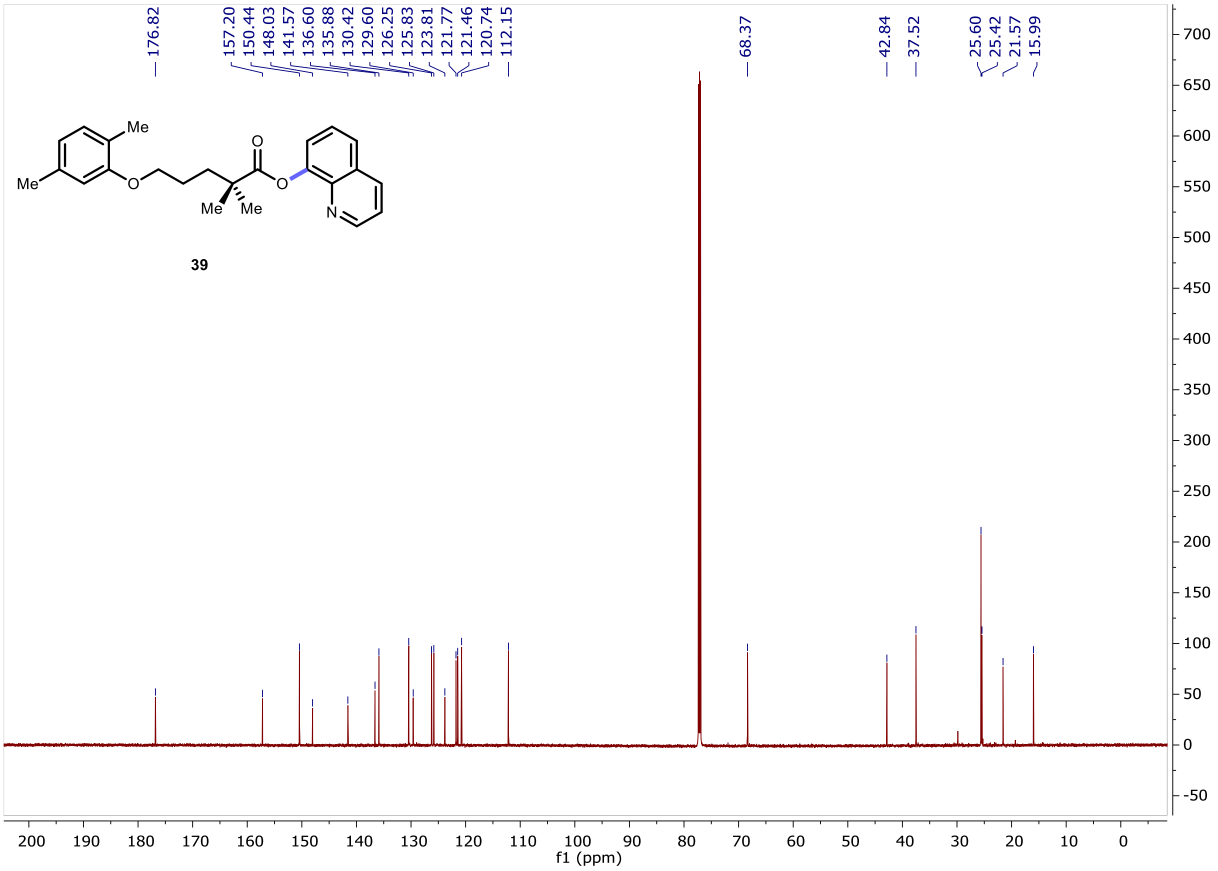

176.82
157.20
150.44
148.03
141.57
136.60
135.88
130.42
129.60
126.25
125.83
123.81
121.77
121.46
120.74
112.15
68.37
42.84
37.52
25.60
25.42
21.57
15.99
Me
O
O
O
Me
Me
Me
N
39
f1 (ppm)

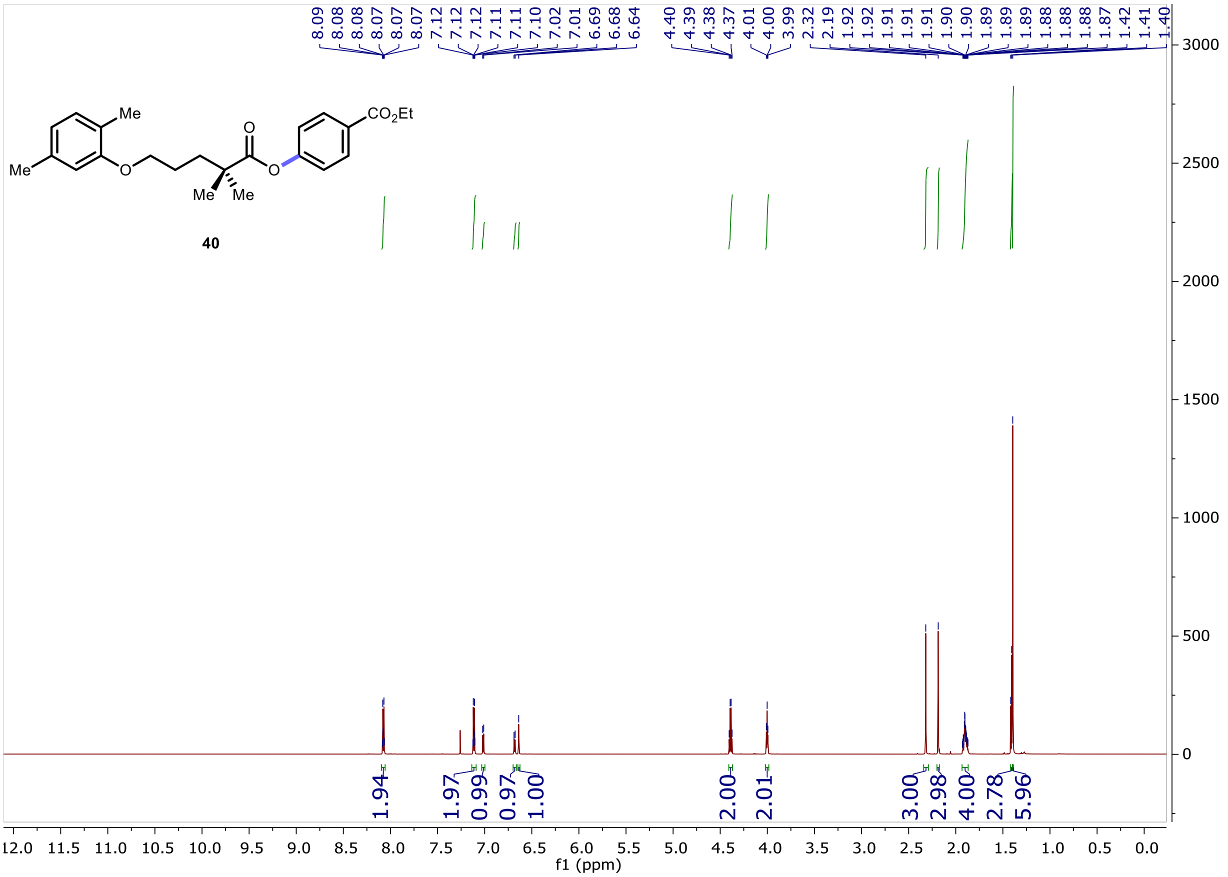
Me
Me
O
O
O
CO2Et
Me
Me
40
8.09
8.08
8.08
8.07
8.07
8.07
7.12
7.12
7.12
7.11
7.11
7.10
7.02
7.01
6.69
6.68
6.64
4.40
4.39
4.38
4.37
4.01
4.00
3.99
2.32
2.19
1.92
1.92
1.91
1.91
1.91
1.90
1.90
1.89
1.89
1.89
1.88
1.88
1.88
1.87
1.42
1.41
1.40
1.94
1.97
0.99
0.97
1.00
2.00
2.01
3.00
2.98
4.00
2.78
5.96
f1 (ppm)

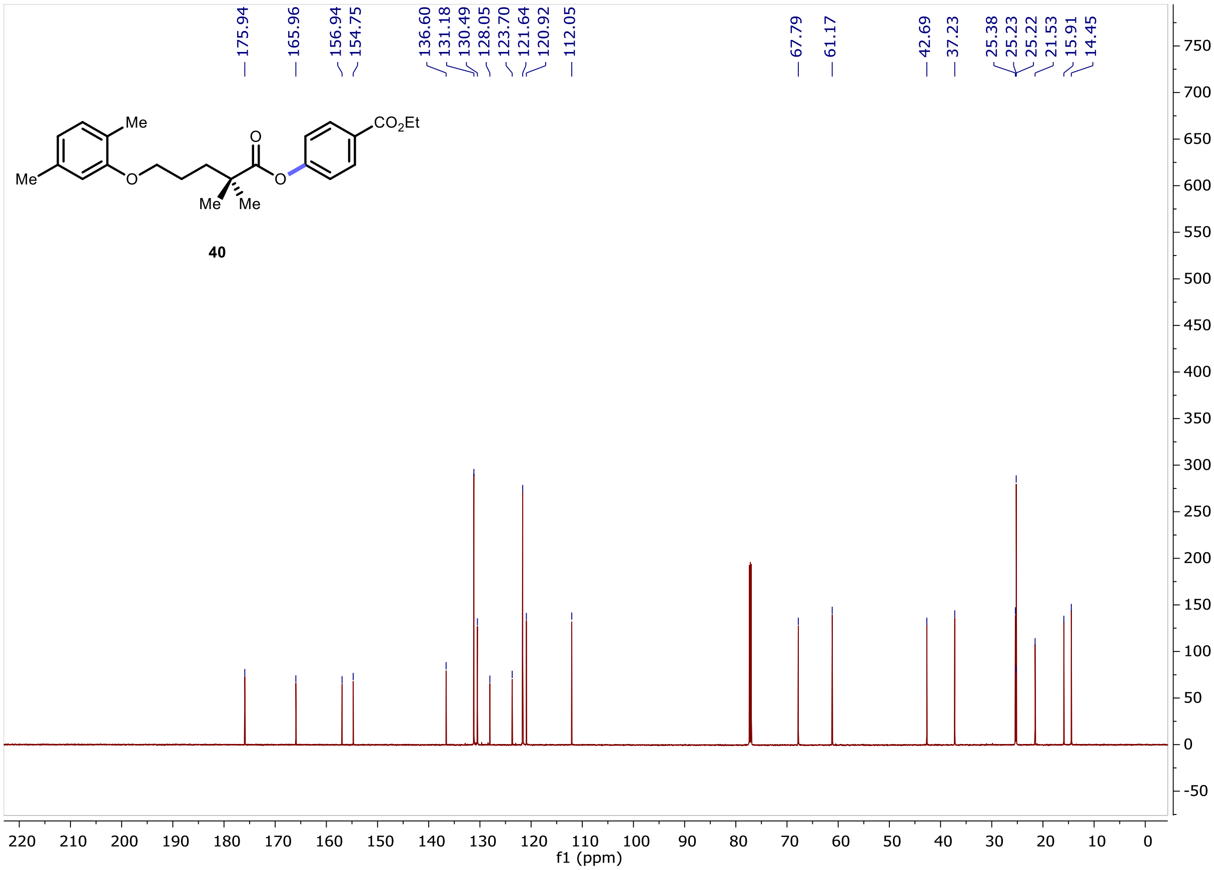
175.94
165.96
156.94
154.75
136.60
131.18
130.49
128.05
123.70
121.64
120.92
112.05
67.79
61.17
42.69
37.23
25.38
25.23
25.22
21.53
15.91
14.45
Me
Me
O
CO2Et
O
O
Me
Me
40
f1 (ppm)

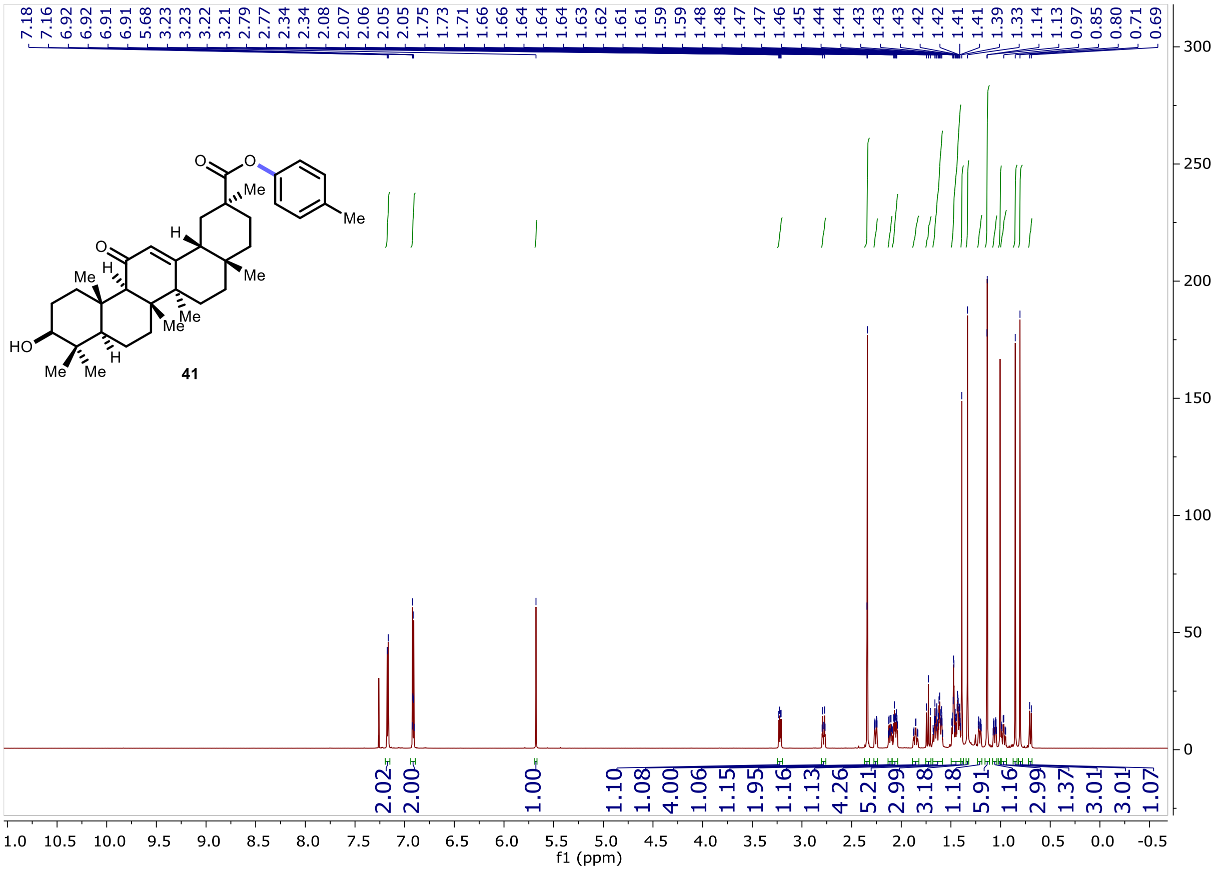
f1 (ppm)
Me
HO
H
41

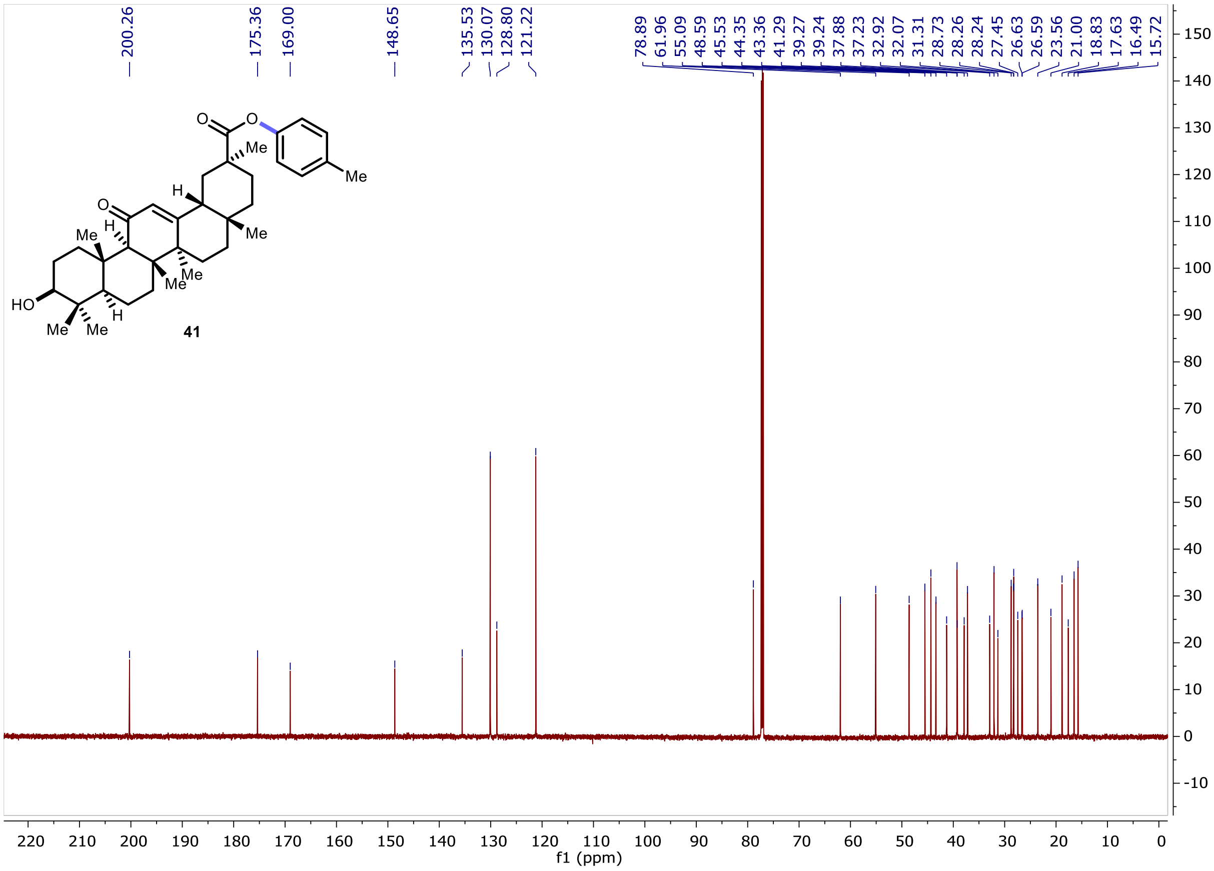
200.26
175.36
169.00
148.65
135.53
130.07
128.80
121.22
78.89
61.96
55.09
48.59
45.53
44.35
43.36
41.29
39.27
39.24
37.88
37.23
32.92
32.07
31.31
28.73
28.26
28.24
27.45
26.63
26.59
23.56
21.00
18.83
17.63
16.49
15.72
O
O
Me
Me
H
O
Me
H
Me
Me
Me
HO
H
Me
Me
41
f1 (ppm)

Supplementary References

1. Cernak, T.; Gesmundo, N. J.; Dykstra, K.; Yu, Y.; Wu, Z.; Shi, Z.-C.; Vachal, P.; Sperbeck, D.; He, S.; Murphy, B. A.; Sonatore, L.; Williams, S.; Madeira, M.; Verras, A.; Reiter, M.; Lee, C. H.; Cuff, J.; Sherer, E. C.; Kuethe, J.; Goble, S.; Perrotto, N.; Pinto, S.; Shen, D.-M.; Nargund, R.; Balkovec, J.; DeVita, R. J.; Dreher, S. D., Microscale High-Throughput Experimentation as an Enabling Technology in Drug Discovery: Application in the Discovery of (Piperidinyl)pyridinyl-1H-benzimidazole Diacylglycerol Acyltransferase 1 Inhibitors. *Journal of Medicinal Chemistry* **2017,** *60* (9), 3594-3605.
2. Mahjour, B.; Cernak, T., Phactor™ – a High Throughput Experimentation Management System. *ChemRxiv* **2020**.
3. Mahjour, B.; Shen, Y.; Liu, W.; Cernak, T., A map of the amine–carboxylic acid coupling system. *Nature* **2020,** *580* (7801), 71-75.
4. Buitrago Santanilla, A.; Regalado, E. L.; Pereira, T.; Shevlin, M.; Bateman, K.; Campeau, L.-C.; Schneeweis, J.; Berritt, S.; Shi, Z.-C.; Nantermet, P.; Liu, Y.; Helmy, R.; Welch, C. J.; Vachal, P.; Davies, I. W.; Cernak, T.; Dreher, S. D., Nanomole-scale high-throughput chemistry for the synthesis of complex molecules. *Science* **2015,** *347* (6217), 49.
5. Bayarmagnai, B.; Matheis, C.; Risto, E.; Goossen, L. J., One-Pot Sandmeyer Trifluoromethylation and Trifluoromethylthiolation. *Advanced Synthesis & Catalysis* **2014,** *356* (10), 2343-2348.
